# Activity-dependent adaptive deep brain stimulation improves gait in Parkinson’s disease

**DOI:** 10.1038/s41591-026-04432-4

**Published:** 2026-06-15

**Authors:** Stefano Scafa, Valeria de Seta, Ruijia Wang, Paula Sánchez López, Andrea Sánchez López, Camille Varescon, Icare Sakr, Nadia Bérard, Lea Bole-Feysot, Céline Deschenaux, Ian Enderli, Yohann Thenaisie, Morgane Burri, Frédéric Merlos, Vanessa Fleury, Benoit Wicki, Ettore Accolla, Andria Tziakouri, Cécile Hübsch, Mayte Castro Jiménez, Julien F. Bally, Alessandro Puiatti, Kyuhwa Lee, Henri Lorach, Antoine Collomb-Clerc, Jocelyne Bloch, Eduardo M. Moraud

**Affiliations:** 1 Department of Clinical Neurosciences, Lausanne University Hospital (CHUV) and University of Lausanne (UNIL), Lausanne, Switzerland; 2 NeuroRestore, Lausanne University Hospital (CHUV) and Ecole Polytechnique Fédérale de Lausanne (EPFL), Lausanne, Switzerland; 3 Institute of Digital Technologies for Personalized Healthcare (MeDiTech), University of Southern Switzerland (SUPSI), Viganello, Switzerland; 4 Neuro-X Institute, École Polytechnique Fédérale de Lausanne (EPFL), Geneva, Switzerland; 5 Department of Neurosurgery, Lausanne University Hospital (CHUV) and University of Lausanne (UNIL), Lausanne, Switzerland; 6 Department of Neurology, Hôpitaux Universitaires de Genève (HUG), Geneva, Switzerland; 7 Faculty of Medicine, University of Geneva, Geneva, Switzerland; 8 Department of Neurology, Hôpital du Valais, Sion, Switzerland; 9 Department of Neurology, Hôpital Fribourgeois, Fribourg, Switzerland; 10 Department of Neurology, Lausanne University Hospital (CHUV) and University of Lausanne (UNIL), Lausanne, Switzerland; 11 Wyss Center for Bio and Neuroengineering, Geneva, Switzerland

**Keywords:** Predictive markers, Parkinson's disease, Therapeutics

## Abstract

Parkinson’s disease leads to a spectrum of locomotor deficits that vary in severity with the nature of daily activities and the fluctuating physiology of patients. Many of these deficits remain inadequately addressed by existing deep brain stimulation therapies that rely on activity-agnostic parameters optimized for cardinal motor symptoms. By contrast, therapies embedding activity-specific parameters have the potential to better address the entire range of symptoms. Here we expose physiological principles that enable real-time decoding of ongoing locomotor activities across motor fluctuations from the neural dynamics of the subthalamic nucleus. This decoding steered activity-dependent adaptations of deep brain stimulation therapies that improved locomotor deficits while preserving efficacy for cardinal motor symptoms across activities of daily living. Our activity-dependent framework provides a blueprint for next-generation neuromodulation therapies that continuously select parameters optimized to the behavioral context and fluctuating physiology of each patient. ClinicalTrials.gov registration NCT06791902.

## Abstract (web summary)

Neural decoding algorithms that leverage physiological principles of locomotor encoding support activity-dependent deep brain stimulation therapies that improve locomotor deficits in people with Parkinson’s disease.

## Main

Parkinson’s disease (PD) leads to a spectrum of locomotor deficits, including heterogeneous gait abnormalities, balance disturbances and freezing of gait (FOG)^1,2^. Although some of these symptoms improve with dopamine replacement pharmacotherapy (L-DOPA) and deep brain stimulation (DBS) of the subthalamic nucleus (STN) or internal globus pallidus, many locomotor deficits remain refractory to these treatments^3,4^. In late-stage PD, up to 90% of individuals experience therapy-resistant locomotor impairments that markedly increase the risk of falls, lead to loss of independence and reduce quality of life^5,6^. Addressing these deficits remains a priority in the treatment of PD^7^.

Conventional DBS therapies use stimulation parameters that are primarily optimized to alleviate rigidity, bradykinesia and tremor^8,9^. These parameters are often suboptimal for alleviating locomotor deficits, and may even aggravate symptoms such as postural instability and FOG^3,10^. Clinical evidence indicates that when DBS parameters are instead tuned to target locomotor deficits, they can mitigate these symptoms^9,11,12^. These observations suggest that optimal DBS therapies must incorporate multiple parameter configurations tailored to both cardinal motor symptoms and locomotor deficits. Moreover, dopamine-related fluctuations and context-dependent physical and cognitive demands can exacerbate the severity of locomotor deficits across daily activities^13,14^. We therefore anticipate that DBS therapies must not only incorporate additional parameters targeting gait deficits, but also dynamically adapt these parameters in real time to ongoing activities and the fluctuating physiology of each individual.

Developing such therapies requires the identification of physiological principles that reveal patient-specific encoding of locomotor activities across motor fluctuations to enable activity-dependent DBS adaptations in real time. The STN is a central hub in the neuronal architecture that encodes motor function and dysfunction, both at rest and across mobility activities^15–20^. Previous work demonstrated that locomotor features and gait deficits can be decoded from local field potentials (LFPs) recorded via DBS electrodes implanted in the STN of people with PD^20–25^. Moreover, implantable neurostimulation platforms with sensing capabilities now enable chronic recordings of STN LFPs to deliver adaptive therapies throughout daily life^26–28^. Therefore, the STN is both a physiologically relevant and technologically accessible target to extract principles for steering adaptive therapies. The identification of principles encoding bradykinesia and rigidity^29–31^ has already enabled adaptive DBS (aDBS) strategies operating in closed-loop that outperform conventional DBS protocols for these symptoms^32–37^.

We reasoned that the STN also constitutes a natural and practical target for extracting physiological principles capable of steering activity-dependent therapies for gait and balance^38–41^. However, these principles remain to be defined. Here, we show that the distinct muscle activation demands underlying daily mobility activities such as sitting, standing, walking and obstacle avoidance are encoded in STN dynamics. Machine learning strategies informed by these principles enabled the implementation of activity-dependent DBS therapies, personalized to each individual, that ameliorated both locomotor deficits and cardinal motor symptoms in real time.

## Results

### Multimodal platform to dissect activity-dependent STN dynamics

We sought to uncover the physiological principles by which STN dynamics encode mobility activities across physiological and therapeutic fluctuations encountered in daily life. Uncovering these principles required mapping electrophysiological signals recorded from the STN onto precise behavioral measurements across a range of locomotor activities and therapeutic conditions, including L-DOPA and STN DBS administered alone or in combination.

**Fig. 1:**
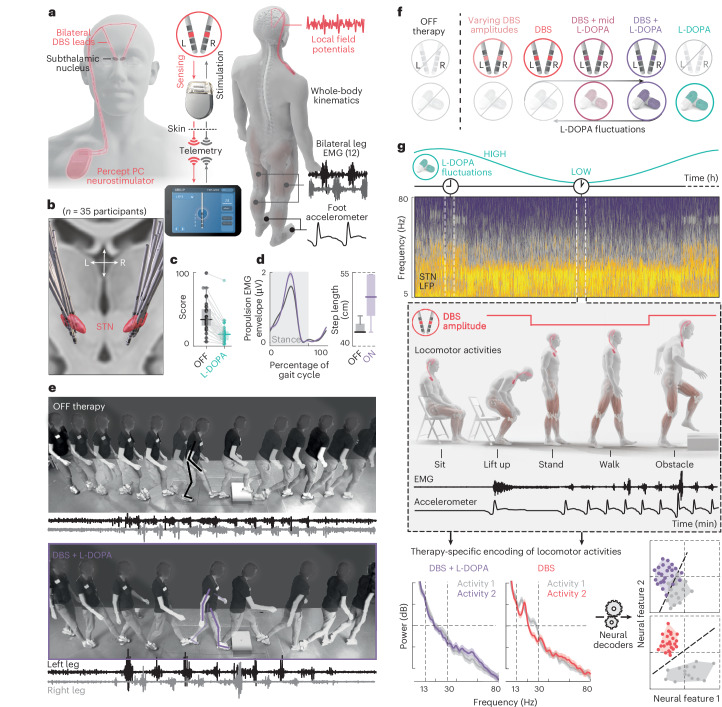
Experimental setup to dissect activity-dependent STN dynamics across therapeutic conditions. **a**, Wireless monitoring platform enabling continuous recording of STN LFPs, bilateral leg muscle activity and whole-body kinematics during unconstrained mobility in participants with PD who were implanted with a last-generation DBS neurostimulator with sensing capabilities. **b**, Anatomical reconstruction of lead placements across the 35 participants included in the study. **c**, Clinical profiles of the included participants (*n* = 35). Boxplots show pre-surgery clinical scores (MDS-UPDRS III) measured in the OFF-therapy condition (no L-DOPA for >12 h), and improvements in response to L-DOPA. **d**, Representative metrics quantifying gait improvements induced by the standard-of-care combination of STN DBS and L-DOPA (ON-therapy condition) versus the OFF-therapy condition after DBS implantation in 1 illustrative participant (*n* = 141 steps in OFF and *n* = 121 in ON, recorded across 12 back-and-forth 10-m walking bouts). Traces represent the mean ± s.e.m. of EMG envelopes from the lateral gastrocnemius muscle, normalized to gait-cycle duration. Boxplots show changes in step length for the same gait cycles. **e**, Chronophotography of an obstacle-avoidance sequence in the OFF-therapy condition (top) and under standard-of-care STN DBS and L-DOPA (bottom), aligned to knee extensor muscle activity (vastus medialis) for the right and left legs. **f**, Therapeutic conditions studied include distinct combinations of STN DBS and L-DOPA, designed to isolate their individual contributions, as well as their interaction over time. **g**, Experimental protocol to dissect the physiological and therapeutic components required to implement activity-dependent DBS therapies for real-world applications. The protocol involves mapping STN spectral dynamics to locomotor activities across L-DOPA fluctuations and changes in DBS amplitude. To capture these dynamics, assessments are performed at selected time windows throughout the L-DOPA cycle. Locomotor performance is evaluated under different DBS amplitudes. Neural data collected during these periods are used to train decoders that discriminate locomotor activities across varying dopaminergic and stimulation conditions, and identify the spectral features that most strongly distinguish between these activities. Dashed lines indicate the limits of the canonical beta band (13–30 Hz) in the power spectral density (PSD). L, left; R, right.

To enable this mapping, we established a wireless platform integrating real-time STN LFP recordings from an implantable pulse generator (IPG) with sensing capabilities^26^, together with high-resolution tracking of full-body kinematics and bilateral leg muscle activity across multiple muscle groups during unconstrained mobility (Fig. 1a and Supplementary Video 1).

We enrolled 35 individuals with advanced PD who exhibited motor fluctuations (Movement Disorder Society Unified Parkinson’s Disease Rating Scale Part III (MDS-UPDRS III) median score = 36 OFF L-DOPA, median score improvement with L-DOPA = 20) (Fig. 1b,c and Supplementary Table 1). Kinematic quantifications confirmed the prevalence of locomotor impairments in this cohort (Fig. 1d,e). All participants underwent bilateral implantation of DBS leads in the STN. Postoperative reconstructions confirmed accurate lead placement (Fig. 1b and Supplementary Fig. 1).

For each participant, we conducted electrophysiological assessments to define low-beta, high-beta and gamma frequency bands using parametric fitting methods^42^ (Supplementary Fig. 2). We further confirmed that the standard-of-care combination of L-DOPA and STN DBS led to improvements in gait quality (Fig. 1d,e).

We then dissected the physiological and therapeutic components that influence activity-dependent STN dynamics by isolating conditions encountered in daily life: (1) standard-of-care L-DOPA + STN DBS; (2) L-DOPA and STN DBS administered independently; (3) varying dosages of both therapies; and (4) the OFF-therapy condition as a baseline for comparison (Fig. 1f,g).

To address the complexity of interactions between experimental paradigms and therapeutic conditions, participants were divided into groups (labeled A–F), each assigned to specific protocols (Methods and Supplementary Table 2). A first cohort included 19 participants recorded in the OFF-therapy condition and under the standard-of-care combination of STN DBS and L-DOPA (group A). Among them, 9 participants (group B) were additionally assessed at multiple time-points to capture L-DOPA pharmacokinetics and 15 (group C) were assessed under STN DBS only. A second cohort included ten participants recorded under L-DOPA only (group D) and eight who were also assessed under varying STN DBS amplitudes (group E). Finally, four participants were recorded during dynamic toggling of DBS amplitude to evaluate the robustness of neural decoders (group F).

### STN dynamics encode locomotor activities in the absence of therapy

Daily mobility encompasses a diversity of locomotor activities, such as standing, walking, turning or navigating environmental constraints—each demanding distinct and often asymmetric activation of bilateral leg muscles. In PD, prior work showed that cortico-subthalamic oscillatory activity correlates with abnormal muscle activation during standing, as well as with muscle vigor during isolated leg movements and walking^21,43–45^. We thus hypothesized that the distinct muscle activation demands underlying daily locomotor activities must also be encoded in STN dynamics.

**Fig. 2:**
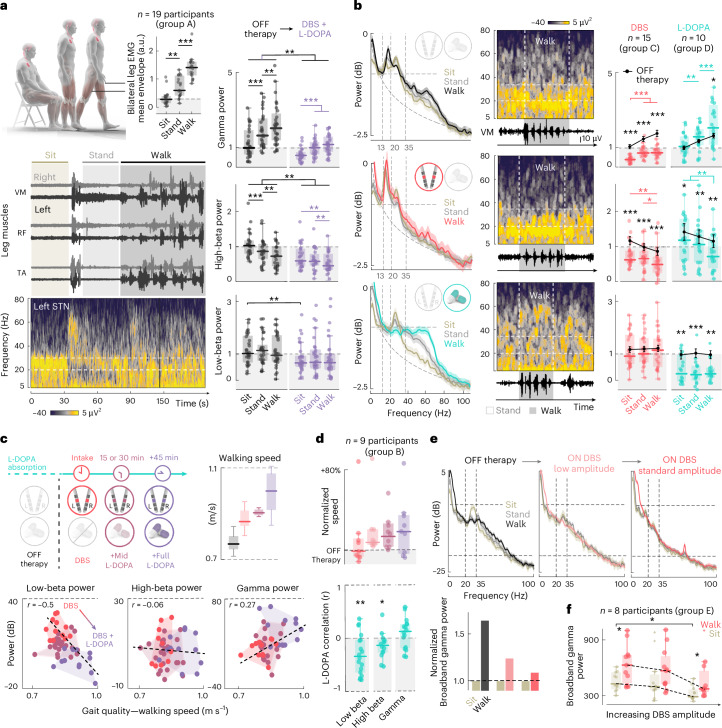
Therapy-dependent encoding of locomotor activities in STN dynamics. **a**, Illustrative example of raw EMG traces for three bilateral leg muscles (vastus medialis (VM), rectus femoris (RF) and tibialis anterior (TA)) aligned to the time-resolved spectrogram of STN LFP during transitions from sitting to standing to walking for one representative participant (PD15). Boxplots represent mean EMG activation (computed from bilateral vastus medialis envelopes), showing a progressive increase in motor demands across locomotor activities (*n* = 19 participants, group A; one-way analysis of variance (ANOVA) with Bonferroni correction: *P* = 1.8 × 10^−12^, *F*_2,54_ = 46.5, *η*^2^ = 0.63; *η*^2^ is the effect size in a one-way ANOVA). Boxplots show STN LFP band-power modulations across locomotor activities for the low-beta (mean frequency range 12.9–19.5 Hz), high-beta (21.6–31.6 Hz) and gamma (37.7–68.8 Hz) bands in the OFF-therapy condition (black) and under the standard-of-care combination of L-DOPA and STN DBS (purple). Power values are normalized to the group median at rest (sit) in the OFF-therapy condition. Dots represent mean power values for each hemisphere (*n* = 33 hemispheres from 19 participants, group A). Not all hemispheres exhibited all three power bands (Methods). Colored stars indicate significant differences between locomotor activities (sit, stand, walk) in the same therapeutic condition. Statistical significance was evaluated using two-way repeated-measures ANOVA for each frequency band, with Greenhouse–Geisser (GG)-corrected *P* values and Bonferroni-corrected post-hoc comparisons (Supplementary Table 4). Significant effects of therapy and locomotor activity were observed in the gamma (*P* = 0.001 and *P* = 1.9 × 10^−10^) and high-beta (*P* = 9.1 × 10^−5^ and *P* = 9.3 × 10^−7^) bands, with marked attenuation of movement-related modulations under L-DOPA + STN DBS. By contrast, low-beta power showed only a modest reduction under therapy (*P* = 0.044), with no significant modulation across locomotor states or interaction effects. **b**, Therapy-specific encoding. Left: illustrative example of PSD (Welch’s method, mean ± 95% confidence interval) of STN LFPs across locomotor activities in the OFF-therapy condition, under STN DBS only, and under L-DOPA only, shown for one participant (PD30). Middle: corresponding time-resolved spectrogram aligned to leg EMG activity (VM) during a sequence of interleaved walking and standing. Right: boxplots showing band-power modulations across locomotor activities under STN DBS only and L-DOPA only. Power values are normalized to the group median at rest (sit) in the OFF-therapy condition. Dots represent mean power values for each individual hemisphere included in the analysis (*n* = 27 hemispheres from the 15 participants in group B under STN DBS only; *n* = 17 hemispheres from the 10 participants in group C under L-DOPA only). Colored stars indicate significant differences between locomotor activities in the same therapeutic condition. Black stars above individual boxplots denote pairwise statistically significant differences relative to the OFF-therapy condition for the same subset of hemispheres (black dots). Statistical significance was evaluated using two-way repeated-measures ANOVA for each frequency band, with GG-corrected *P* values and Bonferroni-corrected post-hoc comparisons (Supplementary Table 5). Significant main and interaction effects were found in the gamma band (STN DBS *P* = 3.8 × 10^−5^ and *P* = 0.002; L-DOPA *P* = 0.020 and *P* = 0.002), whereas high-beta exhibited significant main effects (STN DBS *P* = 1.7 × 10^−4^ and *P* = 6.9 × 10^−5^; L-DOPA *P* = 0.0047 for condition) without interaction under either therapy. These results reflect distinct therapy-dependent modulation patterns: L-DOPA enhanced movement-related increases, whereas STN DBS globally suppressed gamma and beta activity. Low-beta power showed a significant effect only under L-DOPA (*P* = 0.001), remaining unmodulated across activities under STN DBS. **c**, Alterations with L-DOPA pharmacokinetics. Top: experimental protocol to assess the evolving interaction between STN DBS and L-DOPA over time, including gait evaluations at 15, 30 and 45 min following L-DOPA intake. Boxplots show key gait parameters across conditions for participant PD1, illustrating progressive improvements with therapies. Bottom: correlation analyses between average band power and gait quality (walking speed) for the low-beta, high-beta and gamma bands throughout L-DOPA absorption. The individual effects of L-DOPA absorption alongside STN DBS illustrate distinct behaviors over time. Each dot represents the mean band power per walking sequence (ten sequences per condition). Dotted lines represent linear correlations. **d**, Top: boxplots show cross-participant changes in step length for each therapeutic condition (*n* = 9 participants, group B), normalized to the OFF-therapy condition. Kruskal–Wallis test revealed a significant overall difference across L-DOPA absorption times (*H*(4) = 11.28, *P* = 0.0236), although post-hoc pairwise comparisons did not reach significance after correction (lowest *P* = 0.084, for DBS only versus DBS + full L-DOPA). Bottom: Spearman correlation coefficients (*r*) between gait quality (walking speed) and band power in each frequency band, measured throughout L-DOPA absorption. Analyses were performed across 18 hemispheres (same participants, group B). Significant correlations were observed in 9 (50%) hemispheres for the low-beta band, 4 (28%) for the high-beta band and 5 (31%) for the gamma band (*P* < 0.05, Spearman correlation). Group-level comparisons against zero confirmed a significant negative correlation for low-beta STN LFP modulation and gait quality (Wilcoxon signed-rank test *W* = 25, *P* = 0.0084), a weaker but significant effect for high-beta (*W* = 18, *P* = 0.0295) and no significant effect for gamma (*W* = 81, *P* = 0.50). **e**, Alterations with DBS amplitude. Top: representative examples of PSD (mean ± 95% confidence interval of the estimate) from 1 participant (PD24), showing activity-dependent modulations across 3 DBS amplitudes: the standard-of-care amplitude, half that amplitude and 0 mA. Broadband gamma modulation gradually decreases with increasing stimulation, and is replaced by a narrowband gamma peak that also encodes locomotor activities. Bottom: bar plots quantify gamma power modulation across all three conditions, normalized to the resting (sitting) state in the same participant. In **b**, **c** and **e**, dashed lines indicate the limits of the canonical beta band (13–30 Hz) in the PSD. **f**, Boxplots show cross-participant broadband gamma modulations across increasing DBS amplitudes (*n* = 11 hemispheres, group E). Two-way repeated-measures ANOVA revealed a significant main effect of DBS amplitude (*F*_2,18_ = 9.37, *P* = 0.0016), but no significant effect of locomotor state (*F*_1,9_ = 4.25, *P* = 0.069) or interaction between factors (*F*_2,18_ = 1.04, *P* = 0.375). Gamma power decreased with increasing stimulation amplitude, with a significant reduction between the lowest and highest amplitudes at rest (*P* = 0.012). In addition, gamma power was higher during walking than rest at low (*P* = 0.0013) and high (*P* = 0.014) amplitudes, but not at intermediate amplitudes (*P* = 0.49) (Supplementary Table 6). *P < 0.05, **P < 0.01, ***P < 0.001. a.u., arbitrary units.

**Extended Data Fig. 1:**
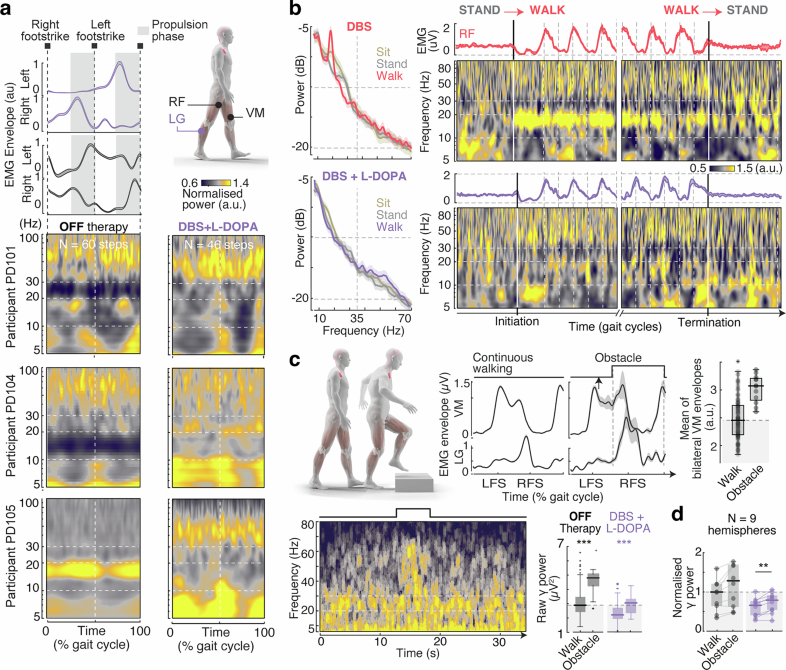
Modulations in activity-dependent STN dynamics are associated with leg muscle activation amplitudes. **(a)** Representative examples of averaged (mean ± s.e.m.) EMG envelopes for two leg muscles (rectus femoris RF and lateral gastrocnemius LG), interpolated between contralateral foot strikes (Right FS and Left FS), and aligned to averaged time-frequency scalograms of STN LFPs from three different participants (PD1, PD3, and PD4, top to bottom). Power is normalized to the standing state. Two therapeutic conditions are shown: OFF therapy (left column) and the standard-of-care combination of L-DOPA and STN DBS (right column). Patients exhibit gait-phase-specific power modulations. In the OFF-therapy condition, these modulations include bursts of low-beta synchrony during the propulsion phase (aligned with LG activation), which fade under standard-of-care therapy and are replaced by gamma-band modulations. However, marked inter-individual differences in gait encoding are evident. **(b)** (*Left*) Power spectral density (PSD, Welch’s method; mean ± 95% confidence interval of the estimate) of activity-dependent STN modulations for participant PD1, shown under two conditions: STN DBS only and the standard-of-care combination of L-DOPA and STN DBS. (*Right*) Corresponding averaged time–frequency scalogram, interpolated across repeated stand-walk-stand sequences (N = 10 repetitions, >10 gait cycles per sequence), aligned to the initiation and termination of walking and to EMG envelopes of the RF muscle. **(c)** Modulations during the obstacle-avoidance task. (*Left*) Averaged EMG envelope traces (mean ± s.e.m.) of two leg muscles (knee extensor vastus medialis VM and ankle extensor LG) normalized in time to the gait-cycle duration, shown for continuous walking and for walking while stepping over an obstacle. Obstacle avoidance elicits increased muscle activation to stabilize the stance leg and propel the body, while lifting the contralateral leg (LFS, left foot strike; RFS, right foot strike). Boxplots quantify the area under the curve (AUC) of bilateral VM EMG envelopes across the two tasks. (*Right*) Representative example of spectral modulations during a walking sequence that includes an obstacle-avoidance step (same as Fig. 1e) and corresponding boxplots of raw gamma power modulations for the same participant (N = 10 repetitions), showing that motor adaptations required to step over the obstacle are associated with increased gamma activity in the STN. **(d)** Cross-participant analysis of normalized gamma-band power (N = 9 hemispheres from the 5 participants who successfully completed the task). Two-way repeated-measures ANOVA revealed a significant main effect of task (*F*(1, 9) = 19.36, *p* = 0.0017). The main effect of therapy was not significant (*F*(1, 9) = 3.29, *p* = 0.10), and the task × therapy interaction only approached significance (*F*(1, 9) = 3.18, *p* = 0.11). Post-hoc comparisons showed that under the standard-of-care therapy, gamma power during obstacle avoidance was significantly higher than during walking (*p* = 0.0033), but only exhibited a trend under the OFF-therapy condition (p = 0.08).

To test this hypothesis, we mapped the relationship between STN LFP modulations and bilateral leg muscle activation during sitting, standing, walking and obstacle avoidance (Fig. 2 and Extended Data Fig. 1). We first conducted this mapping in the absence of therapy (group A, *n* = 19 participants). As expected, transitions from sitting to standing to walking elicited a graded increase in the level of bilateral leg muscle activation that reflected progressive adaptations in the range and complexity of leg movements (Fig. 2a and Extended Data Fig. 1a,c). This activity-dependent increase in muscle activation coincided with a progressive increase in gamma band power, accompanied by a gradual decrease in high-beta power (Fig. 2a, black boxplots). These reproducible patterns contrasted with heterogeneous modulations in low-beta power, which showed participant-specific increases, decreases or no consistent change across the 19 individuals (Fig. 2a and Extended Data Fig. 1a). Similar band-power modulations were observed during the phase of increased leg muscle activation required for obstacle avoidance (Extended Data Fig. 1c,d).

These results confirm that, in the absence of therapy, STN dynamics encode key locomotor activities of daily mobility through specific spectral modulations.

### Standard-of-care therapy alters activity-dependent STN dynamics

L-DOPA and STN DBS are routinely combined to manage the symptomatology of people with PD. This standard-of-care therapy modulates STN dynamics and may also alter locomotor encoding, potentially limiting the suitability of these signals for regulating gait therapies. Because our goal is to develop activity-dependent DBS protocols under the influence of both treatments, we next asked whether the delivery of STN DBS and L-DOPA with standard-of-care parameters alters activity-dependent STN dynamics.

**Extended Data Fig. 2:**
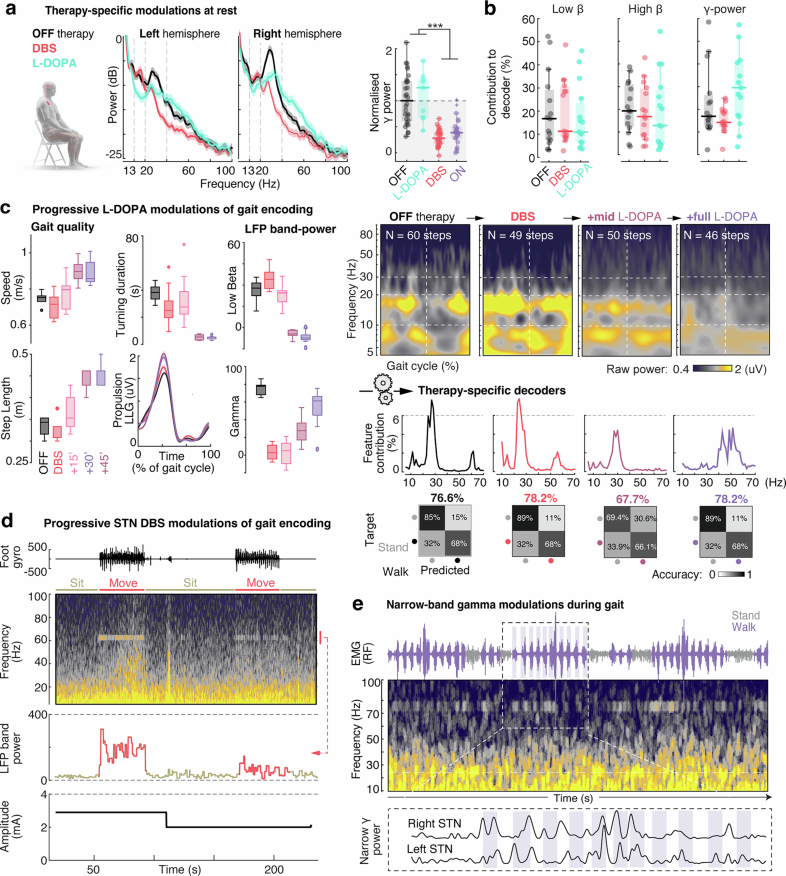
Progressive modulation of activity-dependent STN dynamics across therapeutic conditions and locomotor tasks. **(a)** Illustrative example of power spectral density (PSD) from the right and left STN (participant PD30, same as Fig. 2b), showing distinct therapy-specific modulations across frequency bands during rest. Boxplots quantify changes in gamma power (N = 20 hemispheres). One-way repeated-measures ANOVA revealed a significant main effect of therapeutic condition (*F*(3, 57) = 47.88, *p* = 1.4 × 10^−15^). Bonferroni-corrected post-hoc comparisons showed that the conditions with stimulation (DBS and DBS + L-DOPA) yielded significantly lower gamma power values than the non-stimulation conditions (OFF-therapy and L-DOPA alone; all *p* < 0.001). No significant differences were found between DBS and DBS + L-DOPA, or between OFF-therapy and L-DOPA alone (*p* > 0.5). **(b)** Therapy-specific contributions of each frequency band (low beta, high-beta, and gamma) to neural decoders predicting locomotor movement versus rest (N = 15 participants). Despite patient-specific idiosyncrasies, decoders trained in the OFF-therapy condition relied more heavily on beta activity compared with those trained under STN DBS or under L-DOPA. In contrast, decoders trained under L-DOPA relied more strongly on gamma activity, reflecting a therapy-specific shift in gait encoding as observed during the characterization of activity-dependent modulations within STN dynamics (Fig. 2b). **(c)** Progressive modulations in locomotor quality and gait encoding mediated by L-DOPA. (*Left*) Illustrative example of key gait quality parameters and band-power modulations for participant PD3 during an experimental protocol designed to assess the evolving interaction between STN DBS and L-DOPA over time. Gait evaluations were performed at 0, 15, 30, and 45 minutes following L-DOPA intake, illustrating progressive improvements in locomotor quality with therapy. Number of steps per condition for gait quality assessments: N = 96 (OFF), N = 196 (DBS), N = 148 (15 min), N = 115 (30 min), and N = 113 (45 min). Number of walking sequences used for LFP bandpower and turning duration assessments: 6 (5 turns), 12 (11 turns), and 10 (9 turns) for all other conditions. (*Right*) *Top*: Time-resolved spectral decomposition averaged across gait-cycles, interpolated between consecutive contralateral footstrikes (RFS = Right footstrike, LFS = Left footstrike), for the same participant and under the same therapeutic conditions shown in (Left). Horizontal dashed lines highlight the limits of low and high beta bands. *Bottom*: Contribution of spectral features to neural decoders distinguishing standing from walking, illustrating the gradual shift in gait-related encoding across conditions. Confusion matrices summarize classification performance under each condition. **(d)** Illustrative example of narrowband gamma modulation with movement and DBS amplitude (participant P3, DBS frequency: 125 Hz). (*Top*) Raw spectrograms reveal a narrowband gamma activity that emerges predominantly during movement and progressively diminishes as DBS amplitude is reduced over time (*bottom*). STN LFP band power in the 60–65 Hz range as computed in real time by the implanted DBS neurostimulator (*middle*). This narrowband gamma modulation was not observed across all participants. **(e)** Illustrative example of raw EMG trace for one leg muscle (Rectus Femoris RF) aligned to raw spectrogram of STN LFPs, showing modulations in narrow gamma band power (emerging at half the stimulation frequency, DBS frequency = 160 Hz) during a locomotor task involving interleaved periods of standing, walking, and obstacle avoidance under the standard-of-care therapy L-DOPA + DBS. The inset zooms into one walking sequence and displays bilateral traces of narrow gamma band power, which exhibit modulations time-locked to individual steps.

We first confirmed that the combination of L-DOPA and STN DBS improved mobility in our cohort of participants (group A, *n* = 19). Kinematic and electromyographic analyses revealed improvements in the quality and vigor of gait patterns across the studied locomotor activities (Fig. 1d and Extended Data Fig. 2c). We next examined concomitant changes in STN dynamics. Despite a pronounced reduction in baseline power levels at rest, the gradual modulations in gamma and high-beta power during transitions from sitting to standing to walking were preserved (Fig. 2a). However, the amplitude of these activity-dependent modulations was markedly less pronounced than in the absence of therapy (Fig. 2a, purple boxplots, and Extended Data Figs. 1a–c and 2c). Inspection of low-beta power confirmed the persistent absence of reproducible modulation patterns across participants (Fig. 2a and Extended Data Fig. 1a).

These results confirmed that STN encoding of locomotor activities is preserved under standard-of-care L-DOPA and STN DBS, but that the dynamic range of this encoding is markedly weakened.

### Therapy-specific alterations in activity-dependent STN dynamics

Although L-DOPA and STN DBS jointly contribute to improving locomotor deficits, clinical observations indicate that they exert distinct effects on gait and balance^46^. We reasoned that differences in locomotor function must reflect distinct therapy-specific alterations in the encoding of locomotor activities in STN dynamics, in line with prior observations during upper-limb movements^47,48^. Dissecting these alterations is essential to inform activity-dependent DBS adaptations in patients treated with both treatments.

We performed these assessments in the two cohorts recorded under STN DBS only (group C, *n* = 15 participants) and L-DOPA only (group D, *n* = 10 participants). Modulations in each cohort were quantified relative to their respective OFF-therapy baseline. We found that L-DOPA versus STN DBS altered activity-dependent STN dynamics in strikingly opposite directions. L-DOPA abolished low-beta power modulations across all studied locomotor activities, while concurrently amplifying activity-dependent modulations in high-beta and gamma power (turquoise boxplots and traces in Fig. 2b and Extended Data Fig. 2a,b). These changes altered the contribution of each frequency band to locomotor encoding. By contrast, STN DBS reduced power levels in the high-beta and gamma bands across all activities, thereby weakening their contribution to this encoding (red boxplots and traces in Fig. 2b and Extended Data Fig. 2a,b, and Supplementary Video 1).

These results emphasize that the opposing effects of L-DOPA and STN DBS on the encoding of locomotor activities must be incorporated into the design of algorithms that steer activity-dependent therapies.

### Impact of L-DOPA pharmacokinetics on activity-dependent STN dynamics

L-DOPA pharmacokinetics induce time-dependent motor fluctuations that reflect dynamic changes in brain dopamine levels. We anticipated that these fluctuations must also impose continuously evolving alterations in the encoding of locomotor activities in STN dynamics.

To dissect these alterations, we evaluated changes in STN dynamics during sitting, standing and walking every 15 min for 1 h following L-DOPA administration (group B, *n* = 9 participants). STN DBS remained activated throughout these evaluations. As expected, L-DOPA mediated progressive improvements in gait parameters over the studied period (Fig. 2c,d and Extended Data Fig. 2c). These behavioral improvements were associated with a gradual shift in the contribution of the spectral bands encoding locomotor activities. Concretely, L-DOPA mediated a progressive reduction in low-beta and high-beta power together with an increase in gamma power that correlated with gait quality (Fig. 2c,d). Time–frequency analyses revealed that the gradual reduction in low-beta power during L-DOPA absorption reflected weaker bursts of synchrony during the propulsion phase of walking^21^. By contrast, STN DBS variably increased or decreased the amplitude of these low-beta bursts (Extended Data Fig. 2c).

These findings expose pronounced time-dependent effects of L-DOPA on locomotor encoding, highlighting the need for activity-dependent therapies to incorporate temporal information to maintain stable control as STN dynamics continuously evolve.

### Impact of DBS amplitude on activity-dependent STN dynamics

Activity-dependent DBS strategies are designed to adjust stimulation parameters in real time based on ongoing locomotor activities. In clinical practice, stimulation amplitude is routinely adjusted as a first-line approach^9^, making it a natural control variable for implementing activity-dependent DBS. We therefore examined how amplitude adjustments alter the encoding of locomotor activities.

In the tested cohort (group E, *n* = 8 participants), increasing DBS amplitude led to progressive reductions in gamma power, both while sitting and during locomotor activities (Fig. 2e,f). In a subset of participants, this suppression was accompanied by the emergence of a narrow gamma band at half the stimulation frequency, which also exhibited significant activity-related modulation in participants (Extended Data Fig. 2d,e). The limited occurrence of this signature in our cohort, likely reflecting the postoperative timing of recordings, precluded group-level conclusions. Prior work in chronic individuals established this band as a stable signature of L-DOPA fluctuations, suggesting that movement-related encoding may also be robust and generalizable in chronic recordings.

These observations indicate that DBS amplitude reshapes the encoding of locomotor activities, creating a dependency between stimulation input and feedback signal that must be accounted for to ensure stable activity-dependent control.

### Therapy-specific decoders predict locomotor activities

We next asked whether these physiological principles support the development of machine learning strategies to guide activity-dependent DBS therapies. Given the opposing impact of L-DOPA versus STN DBS on activity-dependent STN dynamics, these therapies may hinder the stability of neural decoders across therapeutic conditions. To address this question, we tested whether a neural decoder trained to discriminate locomotor activities in the absence of therapy remains accurate under standard-of-care L-DOPA and STN DBS.

**Fig. 3:**
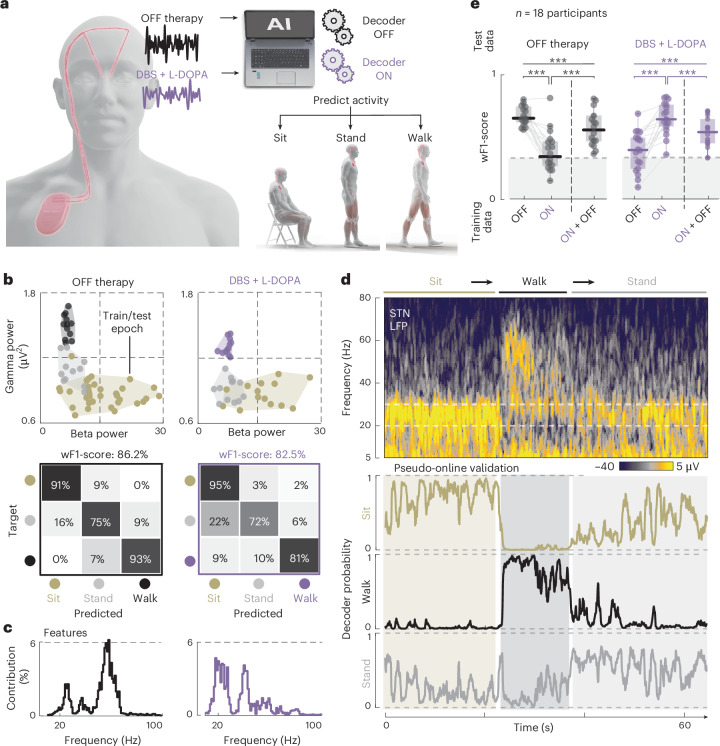
Robust neural decoding of locomotor activities across therapeutic conditions requires therapy-specific decoders. **a**, Computational setup to decode locomotor activities (sit, stand, walk) from STN LFPs acquired in the OFF-therapy condition and under the standard-of-care combination of STN DBS and L-DOPA (ON-therapy condition). Two decoders were trained independently and their performance was then compared across both conditions. **b**, Left: scatter plot of average band-power distributions for the beta and gamma bands illustrating the separability between locomotor states in the OFF-therapy condition (participant PD15). Each dot represents an epoch used for training or testing the decoder. The confusion matrix shows cross-validation performance for this therapy-specific decoder, confirming accurate classification of all 3 states (wF1-score = 86.2%). Right: the same representation under the standard-of-care L-DOPA + STN DBS condition, showing the shift in distributions for each class in the two-dimensional space, and cross-validation performance for a therapy-specific decoder trained in this condition (wF1-score = 82.5%). **c**, Spectral features contributing to each therapy-specific decoder confirm the distinct frequency bands that are most effective for decoding the three locomotor activities under each therapeutic condition (same participant as in **b**). **d**, Pseudo-online validation of real-time decoding. Illustrative example of decoding during a sit–stand–walk transition in the OFF-therapy condition. The raw STN spectrogram and pseudo-online probability traces are shown for a decoder trained and tested on data from the same therapeutic condition (same participant as in **b**). **e**, Boxplots show average decoding performance (wF1-score) during pseudo-online validation across 18 participants (group A; 1 participant had to be excluded due to insufficient task repetitions in the OFF-therapy condition for decoder training). Three decoders are compared: two trained on data from either the OFF-therapy condition (decoder OFF) or the standard-of-care combination of L-DOPA and STN DBS (decoder ON), and a third trained on data from both conditions together (decoder ON + OFF). Each decoder was then tested under each therapeutic condition. When testing in the OFF-therapy condition, repeated-measures ANOVA revealed a significant main effect of training condition (*F*_2,34_ = 35.28, *P* = 5.1 × 10^−9^, partial *η*^2^ = 0.97). Bonferroni-corrected post-hoc tests showed significant differences between all training conditions (*P* < 0.001). Similarly, when testing in the standard-of-care condition (L-DOPA + STN DBS), a significant main effect of training condition was observed (*F*_2,34_ = 32.21, *P* = 1.4 × 10^−8^, partial *η*^2^ = 0.96), with all pairwise comparisons remaining significant after correction (all *P* < 0.001).

**Extended Data Fig. 3:**
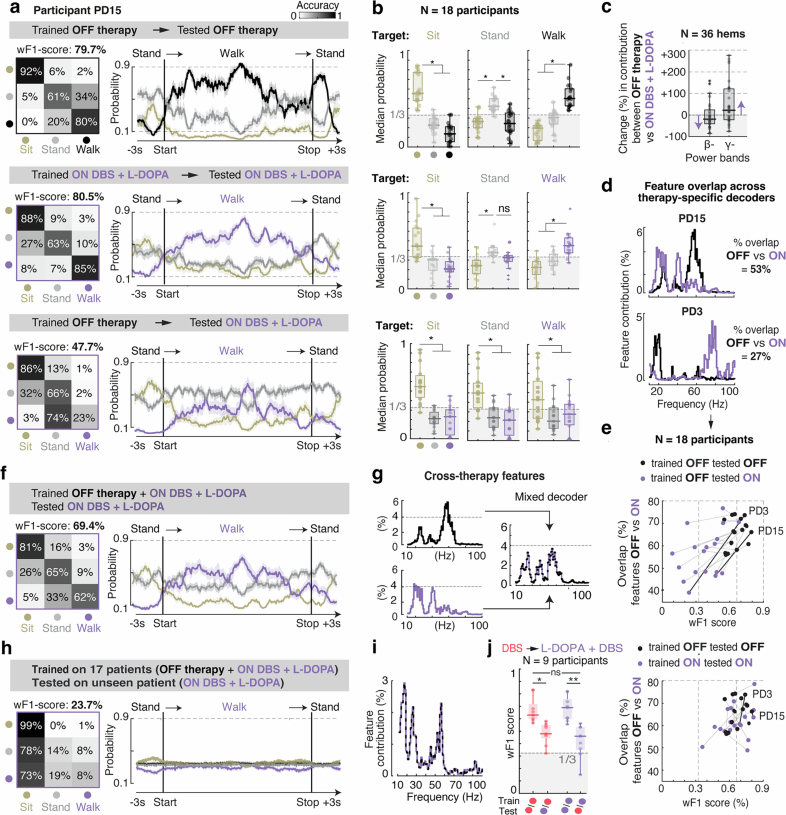
Participant- and therapy-specific neural decoders are required for optimal performance within therapeutic conditions. (**a**) (*Left*) Confusion matrices and wF1-scores pseudo-online performance for decoders trained and tested either within the same or across different therapeutic conditions. (*Right*) Corresponding average probability traces (mean ± s.e.m.) during pseudo-online prediction of the three activities (sit, stand, walk) during a full walking sequence (stand → walk → stand) in participant PD15. (**b**) Boxplots of mean probability values for each class during pseudo-online predictions across all 18 participants, shown for the same three conditions as in (**a**). Therapy-specific decoders accurately classify each activity, whereas cross-therapy decoding exhibits systematic bias toward incorrect classes. One-way ANOVA confirmed statistical significance. (**c**) Comparison of beta (13–35 Hz) and gamma (50–90 Hz) band contributions to therapy-specific decoders. Across all 36 hemispheres, decoders trained under the standard-of-care combination of STN-DBS + L-DOPA rely more heavily on gamma power and less on beta power than decoders trained in the OFF-therapy condition. This pattern is consistent with the modulation observed in these bands under standard-of-care therapy. However, individual feature contributions vary substantially across participants, reflecting the unique spectral encoding of locomotor states in each case. **(d)** The transferability of neural decoders across therapeutic conditions depends on the degree of feature overlap between conditions Two illustrative examples showing the overlap in spectral features between decoders trained in the OFF-therapy state and under the standard-of-care combination of STN-DBS + L-DOPA. **(e)** Correlation between the degree of feature overlap and decoding performance within and across therapeutic conditions. **(f)** Probability traces (mean ± s.e.m.) showing that training decoders on combined data from both therapeutic conditions yields variable performance, with a persistent bias toward incorrect classifications. **(g)** Contribution of spectral features to therapy-specific versus cross-therapy decoders in participant PD15. Decoders trained across both therapeutic conditions distribute weights more broadly across frequency bands, reducing spectral specificity and overall classification performance. **(h)** Illustrative example of average probability traces (mean ± s.e.m.) and confusion matrix for a cross-therapy decoder trained on data from 17 participants and tested on an unseen participant. Predictive accuracy displays chance-level performance. **(i)** Spectral feature contributions across participants reveal three dominant frequency bands (low-beta, high-beta, and gamma), consistent the spectral components commonly associated with movement encoding in patients with Parkinson’s disease. However, this generalized approach fails to capture participant-specific modulations critical for accurate decoding. **(j)** Cross-therapy performance of decoders trained and tested under STN DBS alone versus the standard-of-care combination of STN DBS and L-DOPA. Repeated-measures ANOVA revealed a significant main effect of training condition (F(3, 24) = 15.08, p = 1.0 × 10^−5^). Bonferroni-corrected post-hoc tests showed that decoders trained and tested within the same therapeutic condition performed significantly better than when trained in the opposite condition (train DBS–test DBS > train DBS–test DBS + L-DOPA, p = 0.044; train DBS + L-DOPA–test DBS + L-DOPA > train DBS–test DBS + L-DOPA, p = 0.010), whereas no difference was found between the two within-condition configurations (p = 1.0). wF1-scores: weighted F1-scores.

We developed a machine learning pipeline based on personalized Random Forest decoders^49^ trained to discriminate three classes of locomotor activities: sitting, standing and walking (Fig. 3a). In the absence of therapies, decoders classified these activities with good accuracy (Fig. 3b,c and Supplementary Fig. 3; average cross-validation weighted F1 score (wF1) for the 3 classes across *n* = 18 participants: 69%). To approximate real-time deployment, performance was further validated pseudo-online by replaying data chronologically (Fig. 3d,e, Extended Data Fig. 3a,b and Supplementary Video 2; average wF1-score across *n* = 18 participants: 66.5%). However, the accuracy of these decoders declined sharply when L-DOPA and STN DBS were administered (average pseudo-online wF1-score across *n* = 18 participants: 38.2%; Fig. 3e and Extended Data Fig. 3a,b). This decline reflected the expected shift in activity-dependent spectral features under the standard-of-care therapy, which led to overlaps between the spectral signatures of different classes (Fig. 3c and Extended Data Fig. 3c–e). To compensate for these overlaps, we trained separate decoders using STN LFPs collected under the standard-of-care combination of L-DOPA and STN DBS (Fig. 3b, Extended Data Fig. 3a,b,e and Supplementary Fig. 3). We also trained and tested decoders under STN DBS alone (Extended Data Fig. 3j). These therapy-specific decoders restored accurate classification of locomotor activities under their respective therapeutic condition (average cross-validation wF1-score across participants: 63.4% for combined L-DOPA and STN DBS and 64.9% for STN DBS alone), but failed to generalize across conditions (37.8% pseudo-online performance) (Fig. 3e and Extended Data Fig. 3a,b,j). Feature contribution analyses revealed that each decoder relied on distinct spectral bands (Fig. 3c and Extended Data Fig. 3g).

We finally asked whether a single decoder trained across all therapeutic conditions—without therapy and under standard-of-care therapy—generalizes across conditions. This unified decoder failed to classify locomotor activities accurately, because of overlapping activity-specific spectral features that a single decoder could not disentangle (Fig. 3e and Extended Data Fig. 3f). Similarly, training across participants failed to generalize to individual patients, underscoring the need for personalized decoders (Extended Data Fig. 3h,i).

These results demonstrate that locomotor activities can be predicted from STN dynamics with accuracy, but therapy-specific spectral shifts in activity-dependent encoding prevent a single decoder from generalizing across therapeutic conditions.

### Modular decoding preserves performance across therapeutic conditions

Therapy-specific shifts in activity-dependent STN dynamics that impact decoding performance are not static, but instead evolve over time with fluctuations in L-DOPA levels and changes in DBS parameters. We therefore anticipate that continuous decoding across changing therapeutic conditions requires combining multiple decoders, each selected according to the ongoing therapeutic state. We thus implemented a modular framework integrating two therapy-specific decoders (layer 1) and a classifier that dynamically switches between them (layer 2).

**Fig. 4:**
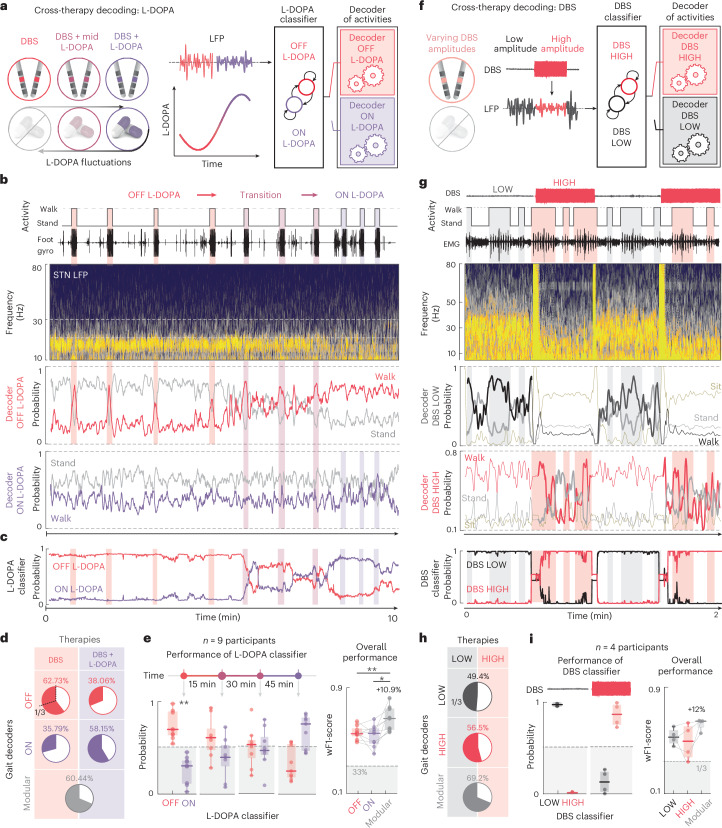
A modular decoding framework maintains prediction accuracy across therapeutic conditions. **a**, A modular decoding framework predicts locomotor activities across L-DOPA pharmacokinetics. Two therapy-specific decoders are trained independently, one under DBS alone (decoder OFF L-DOPA) and another under combined DBS + L-DOPA (decoder ON L-DOPA). An intermediate layer (L-DOPA classifier) selects the appropriate decoder based on L-DOPA-related features. Classifier hyperparameters are optimized to filter out fast, locomotor-related modulations while preserving slower, L-DOPA-induced changes in STN LFPs; this tuning is performed using a genetic algorithm that identifies the parameter configuration best suited to discriminate ON and OFF L-DOPA states (Extended Data Fig. 5). **b**, Representative example of raw STN LFP spectrogram (top) and pseudo-online probability traces for each therapy-specific decoder of locomotor activities (bottom) during 10 back-and-forth walking sequences over a 10-min task, initiated 15 min after L-DOPA intake (participant PD3). As L-DOPA is progressively absorbed, STN dynamics and decoder performance evolve. **c**, Raw probability traces during pseudo-online implementation of the L-DOPA classifier over the same recording. **d**, Average pseudo-online performance (wF1-score) for each therapy-specific decoder under DBS alone and under standard-of-care DBS + L-DOPA, highlighting the limited cross-condition generalizability of each decoder (decoder OFF L-DOPA mean wF1-score = 38.06%, decoder ON L-DOPA mean wF1-score = 35.79%). The modular framework markedly improves overall decoding accuracy across the two conditions. **e**, Left: boxplots show pseudo-online performance of the L-DOPA classifier at 15, 30 and 45 min post L-DOPA intake (*n* = 9 participants, group B), illustrating sensitivity to the gradual absorption of L-DOPA (Wilcoxon signed-rank test: DBS only *W* = 45, *P* = 0.0039; L-DOPA + 15 min *W* = 34, *P* = 0.203; L-DOPA + 30 min *W* = 25, *P* = 0.820; L-DOPA + 45 min *W* = 6, *P* = 0.0547). Right: the modular framework yielded higher decoding performance than either decoder alone. An improvement of +10.9% was observed in wF1-score relative to the best of the other 2 single-condition decoders. Repeated-measures ANOVA revealed a significant main effect of decoder (*F*_2,16_ = 12.29, *P* = 5.8 × 10^−4^, partial *η*^2^ = 0.61). Bonferroni-corrected post-hoc tests showed significant differences between the modular decoder and decoders trained in either OFF (*P* = 0.0025) or ON (*P* = 0.0232) conditions alone. No difference was observed between decoders trained OFF and ON (*P* = 0.87). **f**, A modular decoding framework predicts locomotor activities across two DBS amplitudes. Two decoders are trained independently: 1 at the standard-of-care stimulation amplitude (decoder DBS HIGH) and 1 at 0 mA (decoder DBS LOW). A DBS classifier selects the appropriate decoder based on stimulation-related features in STN LFPs. The hyperparameters of the classifier are optimized using a genetic algorithm to detect rapid stimulation-induced changes while ignoring slower, activity-related modulations. **g**, Illustrative example of DBS profile (top), knee extensor EMG aligned to raw STN LFP spectrogram (middle) and pseudo-online decoder probability traces (bottom) during repeated walking sequences (participant PD24). DBS amplitude was manually toggled between low and high values independently of task execution. **h**, Pie charts show average cross-validation performance (wF1-score) across the full trial. The modular framework improves decoding accuracy. **i**, Boxplots show pseudo-online classification accuracy and decoding performance (wF1-score and median probability) across the 4 participants tested (group F), illustrating a +12% improvement when using the modular decoder relative to the best decoder trained in a single condition. No statistical analysis was performed, given the limited sample size. *P < 0.05, **P < 0.01.

**Extended Data Fig. 4:**
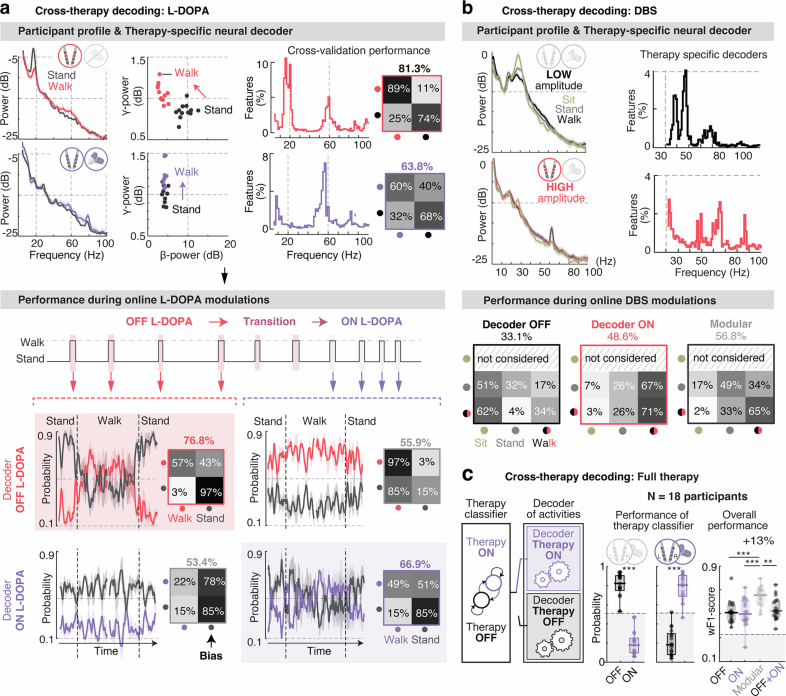
A modular decoding framework improves accuracy and generalization across therapeutic conditions. **(a) (***Top*) Changes in activity-dependent STN dynamics mediated by L-DOPA in participant PD3 (displayed in Fig. 4a-e). The power spectral density (Welch’s method; mean with 95% confidence interval) and epoch distributions in feature space are presented for two activities (stand and walk), alongside the predictive performance of therapy-specific decoders and the corresponding neural features identified for each therapeutic condition. (*Bottom***)** Decoder performance during online L-DOPA fluctuations, illustrating the progressive change in classification accuracy as L-DOPA is absorbed. **(b) (***Top*) PSD (mean with 95% confidence interval) illustrate changes in activity-dependent STN dynamics mediated by differences in DBS amplitude (0 mA vs. standard-of-care amplitude) in participant PD24 (shown in Fig. 4f-i), alongside the neural features identified for each therapeutic condition. (*Bottom*) Performance of each therapy-specific decoder during a recording in which DBS amplitude was manually toggled between the two settings, illustrating the progressive change in classification accuracy as L-DOPA is absorbed. **(c)** The modular decoding framework maintains prediction accuracy when generalizing across the most distinct therapeutic conditions (OFF therapy and standard-of-care STN-DBS plus L-DOPA). Significant differences were observed both during the OFF condition (Wilcoxon signed-rank test: z = 3.62, p = 2.9×10^−4^, W = 153) and during the standard-of-care ON condition (z = −3.57, p = 3.5×10^−4^, W = 1).

**Extended Data Fig. 5:**
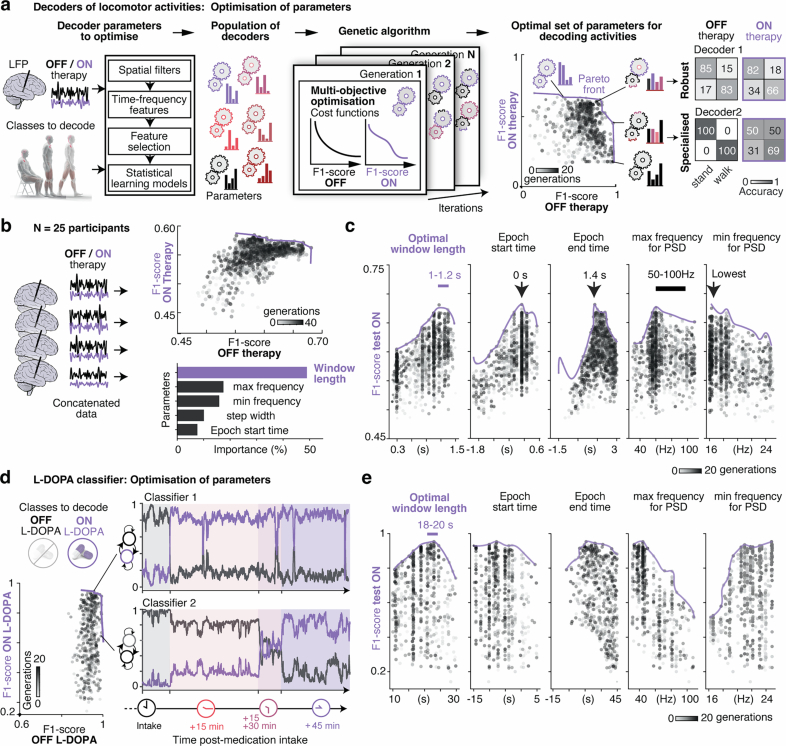
Genetic algorithm optimizes hyper-parameters of neural decoders and therapy classifiers. **(a)** Schematic of the multi-objective optimization pipeline using genetic algorithms to automate feature selection. An initial population of 50 decoders with different parameters is iteratively evolved through mutation, crossover, and selection over 20 generations to maximize model performance based on a predefined fitness criteria. The final output forms a Pareto front of non-dominated solutions balancing competing objectives, namely accuracy and robustness across therapeutic conditions. Confusion matrices illustrate the performance of two representative decoders along this Pareto front: Decoder 1 prioritized robustness across therapeutic conditions, whereas Decoder 2 was optimized for accuracy within one condition. **(b)** Application of the pipeline to identify and optimize key parameters for decoding locomotor activities (sit, stand, walk) across participants. Parameters optimized include: (i) the epoch window, (ii) the frequency range, and (iii) the window length used for spectral feature extraction. Feature importance analysis revealed window length as the most influential parameter, followed by the upper and lower frequency bounds. **(c)** Scatter plot of parameter values across generations for all assessed features. The optimal value maximizing decoding performance is indicated for each parameter. Window length values consistently converged in the range of 1.0–1.2 seconds. This duration matches the natural dynamics of different activities being decoded (gait cycles are in the range of ~1 s, and standing phases lasted a few seconds during our experiments) **(d)** Application of the same pipeline to identify and optimize L-DOPA therapy classifiers. Two representative classifiers illustrate optimized parameters for accuracy (Classifier 1) and robustness (Classifier 2). **(e)** Optimal window lengths for L-DOPA classifiers ranged between 18–20 seconds, consistent with the slower pharmacokinetics of L-DOPA absorption. The differential tuning of parameters for activity decoders versus therapy classifiers leverages the distinct temporal dynamics of locomotion and therapy-induced effects to disentangle their respective contributions from the same neural signal.

We first tested whether this modular framework maintains accurate decoding of locomotor activities despite L-DOPA fluctuations (Fig. 4a). We trained two decoders, one under standard-of-care L-DOPA + STN DBS, and one under STN DBS alone (Fig. 4b and Extended Data Fig. 4a). We then optimized a classifier to estimate the probability of being under the influence of L-DOPA (Fig. 4c). Because L-DOPA fluctuations unfold over slower timescales than locomotor-related modulations, we tuned the classifier’s hyperparameters to filter out fast movement-related dynamics, while preserving slower L-DOPA-induced changes. This tuning was guided by a genetic algorithm that identified the optimal hyperparameter configuration (Extended Data Fig. 5). We then assessed performance in the nine participants who performed walking tasks at multiple time-points post L-DOPA intake (group B). For all participants, the classifier correctly predicted medication states despite individual differences in absorption times (pseudo-online performance: 69.5% OFF L-DOPA, 75.4% ON L-DOPA; Fig. 4d,e and Extended Data Fig. 4a). In turn, the modular framework enabled accurate decoding of locomotor activities under each medication level, accommodating the progressive shift in spectral encoding caused by the absorption of L-DOPA (Fig. 4d,e and Supplementary Video 2).

We then asked whether this modular framework also maintains accurate decoding during adaptations of DBS amplitudes (Fig. 4f and Extended Data Fig. 4b). For this, we implemented two therapy-specific decoders to predict locomotor activities under high DBS amplitude (standard-of-care) or low DBS amplitude (0 mA), and trained the classifier to detect the amplitude of STN DBS (Fig. 4g). We then tested the efficacy in the four individuals undergoing dynamic amplitude toggling (group F). Despite expected alterations in activity-dependent STN dynamics with changing DBS amplitude, the modular framework maintained high decoding accuracy (Fig. 4h,i and Extended Data Fig. 4b).

**Extended Data Fig. 6:**
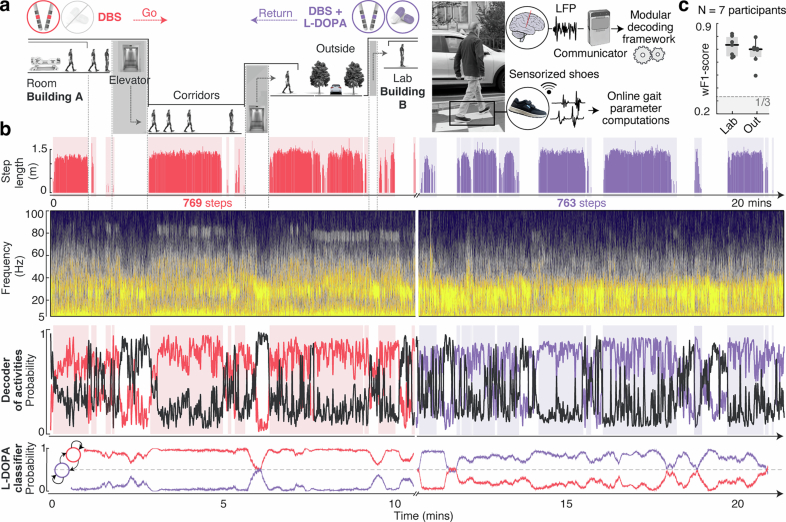
The modular decoding framework maintains prediction accuracy in out-of-laboratory settings. **(a)** Experimental setup to evaluate the real-world transferability of the modular decoding framework. Participants ambulated within hospital environments from building A to building B (~1 km), navigating crowded hallways, elevators, and outdoor environments. Locomotion was continuously tracked using sensorized shoes with embedded machine learning algorithms that detect walking events and compute gait parameters in real time, synchronized with STN LFP recordings. The outbound path was performed under STN DBS alone (medication withdrawn >12 h), and the return under the standard-of-care combination of STN DBS and L-DOPA (~90 minutes post-intake). **(b)** Illustrative example of decoding performance across real-world mobility (participant PD20). Top: Step length measured across >1,500 gait cycles during unconstrained walking. Middle: Corresponding raw STN LFP spectrogram Bottom: Pseudo-online computation of decoder probabilities from the selected activity decoder (black: stand; colored red/purple: walk), aligned to probabilities from the L-DOPA classifier (red: OFF L-DOPA; purple: ON L-DOPA). **(c)** Boxplot represents the performance (wF1-score) of activity decoders trained under controlled laboratory conditions and tested during real-world ambulation, across the 7 participants recorded. No significant difference was found between the two conditions (Wilcoxon signed-rank test, *W* = 19, *p* = 0.47). wF1-score: weighted F1-score.

We finally assessed the robustness of the modular decoding framework in out-of-laboratory settings (Extended Data Fig. 6). We monitored locomotor activities using sensorized shoes with embedded artificial intelligence algorithms that computed spatiotemporal gait parameters as participants ambulated across indoor and outdoor environments^50^ (Extended Data Fig. 6a). These ecological assessments involved frequent transitions between standing and walking in crowded corridors, elevators and while navigating obstacles. Despite these variable environmental conditions, the modular framework maintained accurate decoding of locomotor activities across therapeutic conditions (Extended Data Fig. 6b,c).

This decoding framework provides a blueprint to translate the principles of activity-dependent encoding into adaptive therapies that adjust stimulation to activity demands and fluctuating physiology.

### On-device implementation of activity-dependent DBS

We finally sought proof-of-concept for this decoding framework to steer activity-dependent DBS therapies targeting both cardinal motor symptoms and locomotor deficits.

The physiological principles of activity-dependent encoding in the STN showed that accurate discrimination of locomotor activities across therapeutic conditions requires the full spectrum of frequency bands, beyond the beta band typically accessible in commercial devices. Furthermore, targeting cardinal motor symptoms versus locomotor deficits with DBS necessitates versatile control of stimulation parameters.

**Fig. 5:**
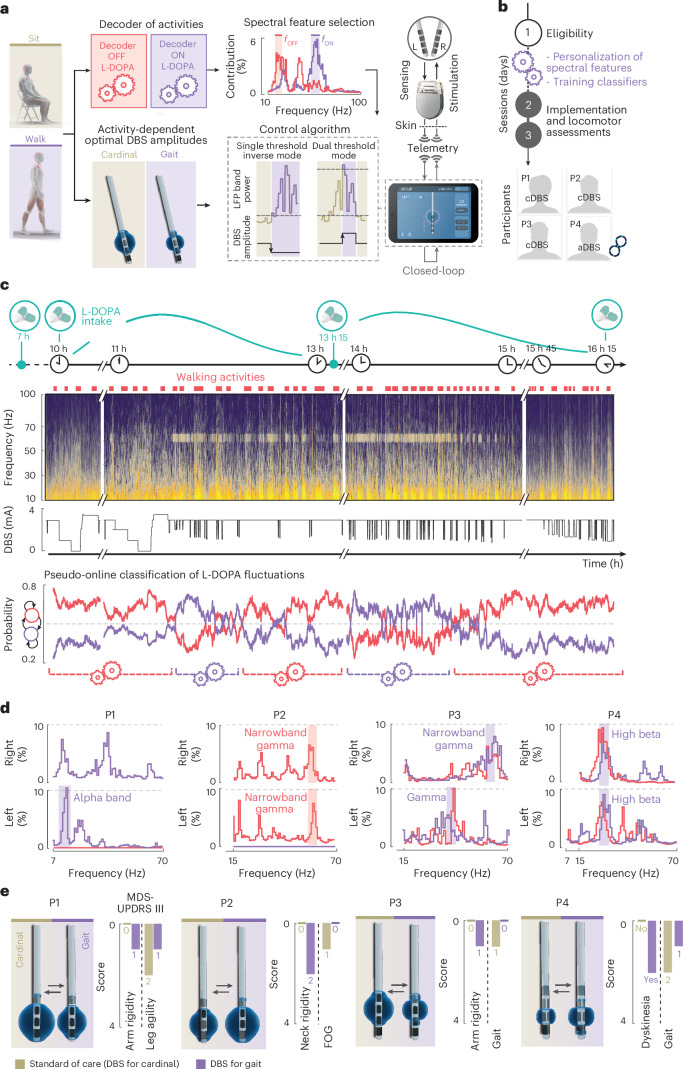
Implementation of activity-dependent DBS therapies targeting patient-specific locomotor deficits. **a**, On-device implementation. Experimental setup for implementing activity-dependent DBS protocols using an implantable DBS platform with investigational closed-loop capabilities. Therapy-specific decoders (OFF L-DOPA, ON L-DOPA) identify the most robust frequency band for discriminating locomotor activities (sit, walk) in each participant and condition. In parallel, optimal DBS amplitudes for alleviating the most bothersome locomotor deficits are determined individually, compared with standard-of-care DBS amplitudes, and used to guide algorithm selection. Activity-dependent protocols are implemented in a DBS neurostimulator with real-time control capabilities, including upregulation or downregulation of stimulation amplitude. *f*_OFF_, frequency in the OFF L-DOPA condition; *f*_ON_, frequency in the ON L-DOPA condition. **b**, Timeline of the feasibility clinical study involving three sessions. During the eligibility session, cardinal motor symptoms, locomotor deficits, impedance values and STN LFP signal quality were assessed through a battery of clinical tasks. Participants meeting inclusion criteria proceeded to two additional full-day sessions to evaluate the safety and preliminary efficacy of activity-dependent aDBS. Following this session, the modular decoding framework was implemented to identify individualized neural signatures of locomotor states across varying stimulation amplitudes and dopaminergic conditions. Four participants were enrolled in the study. Three participants (P1–P3) were treated with continuous DBS. One participant (P4) was treated with beta-based aDBS optimized over several clinical sessions. **c**, Day-long monitoring and decoder training (participant P3). Top: representative example of concatenated STN LFP recordings acquired during a full-day session (session 2) in participant P3, across evolving L-DOPA conditions and varying STN DBS amplitudes, both at rest and during walking activities. Middle: probability traces from the pseudo-online implementation of the L-DOPA classifier, used to guide the selection of spectral features and control parameters according to participant’s ongoing state. Estimated alternations between ON and OFF L-DOPA states fluctuated throughout the day, consistent with medication intake. Locomotor symptoms evolved in parallel. **d**, Therapy-specific decoders of locomotor activities identify the most discriminative spectral feature for each hemisphere in the 7–70 Hz range, compatible with implantable platform constraints for real-time control. The lower bound was increased to 15 Hz in cases in which low-frequency components raised concerns about walking-related artifacts. The optimal frequency band for each hemisphere (shaded red or purple) was selected from those suggested by the decoders, evaluated during online assessments, and iteratively refined based on real-time performance. When spectral features were similar across low- and high-L-DOPA conditions, the same frequency band was selected to enhance stability during real-time adaptations. In cases in which decoding performance from one hemisphere was insufficient, a unilateral control signal from the contralateral hemisphere was used to modulate DBS amplitudes bilaterally (Extended Data Table 1). Optimal spectral features differed across participants and hemispheres (P1, alpha; P2 and P3, broadband or narrowband gamma; P4, high-beta). Participant P2 exhibited severe gait deficits (FOG) only under low L-DOPA and did not require DBS adaptations under high L-DOPA. The selected frequency bands corresponded to the most informative spectral features in that therapeutic condition. P1 could not be assessed in the low L-DOPA condition due to severe fatigue and motor impairments that prevented completion of the required clinical evaluations. **e**, DBS-related changes in the most bothersome symptoms. Optimal DBS configurations for cardinal motor symptoms (standard-of-care, yellow) versus locomotor deficits (purple) in each participant, together with MDS-UPDRS III subscores for the most bothersome cardinal and locomotor items under each stimulation amplitude. Cardinal items comprised upper-limb, neck rigidity and dyskinesia, whereas locomotor items included gait, FOG and lower-limb agility.

To meet these requirements, we used a commercially available neurostimulation platform with investigational features for advanced sensing and control (Fig. 5). These features supported both upregulation and downregulation of stimulation amplitude, guided by continuous feedback of STN LFP signatures across the entire frequency spectrum (7.8–100 Hz) (Fig. 5a and Supplementary Fig. 4a,b). However, monitoring of neural activity was limited to a single manually defined frequency band per hemisphere, and DBS adaptations could only be triggered by threshold crossings^51^, which limited detection to two discrete states. We thus targeted the most distinct locomotor activities—sitting and walking.

To identify the most discriminative frequency band under these constraints, we applied the first layer of our modular decoding framework to compute the optimal spectral feature for classifying sitting versus walking in each hemisphere (Fig. 5a–d). Decoders were trained separately for high- and low-L-DOPA conditions, while pooling across DBS amplitudes to ensure stability once implemented for real-time control (Supplementary Fig. 4a,b). Therapy-specific decoders identified optimal spectral features that typically differed between medication states and across hemispheres (Fig. 5d). On-device algorithms were programmed using these features for real-time adaptations.

Because the full modular decoding framework could not be implemented directly in the device, we implemented the second layer (L-DOPA classifier) pseudo-online in an external computer to inform the optimal DBS parameters based on the ongoing state of the participant (Fig. 5c). This implementation leveraged the core advantages of our modular decoding framework to validate activity-dependent therapies within current implantable system constraints.

### Activity-dependent DBS therapies alleviate locomotor deficits

We finally conducted a feasibility clinical trial in four participants with advanced PD and severe motor fluctuations, whose gait impairments persisted despite optimized DBS settings (ClinicalTrials.gov registration NCT06791902; Fig. 5b and Supplementary Table 3).

**Extended Data Fig. 7:**
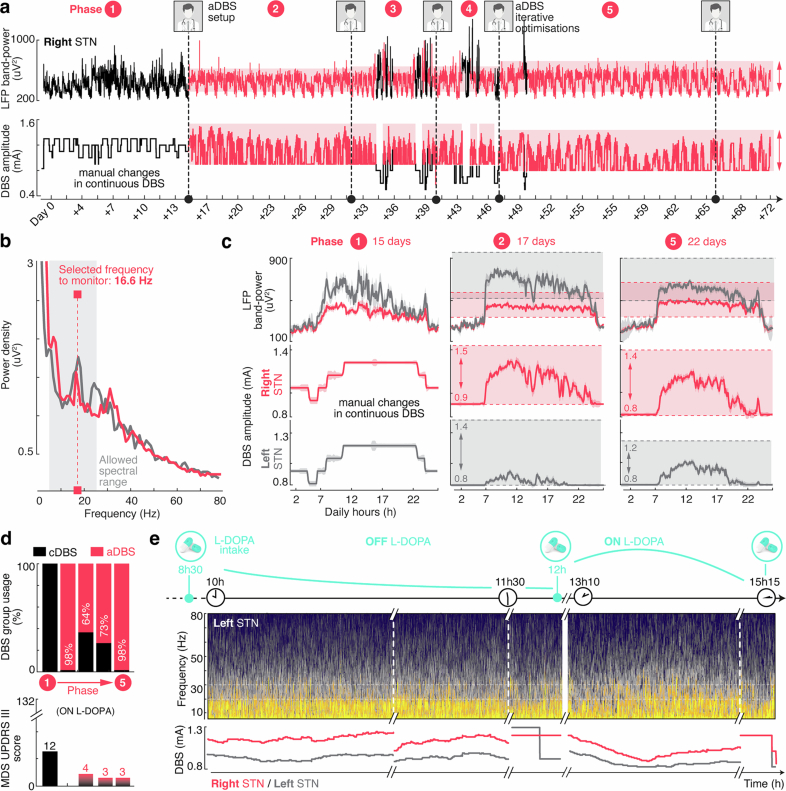
Longitudinal optimization and evaluation of beta-based adaptive DBS in participant P4. **(a)** Traces of longitudinal LFP band power (BrainSense™ Timeline) and DBS stimulation amplitudes across more than 10 weeks of follow-up in participant P4, encompassing setup, optimization, and assessment of beta-based adaptive DBS. Six in-clinic sessions were conducted, including an initial visit to identify participant-specific frequency bands, followed by five sessions dedicated to setup and iterative optimization of adaptive therapy parameters. Throughout the continuous DBS period (Phase 1), the participant regularly attempted to manually adjust stimulation to improve symptoms. Across the subsequent optimization sessions, threshold values and DBS amplitude limits were iteratively refined based on adaptation behavior and participant feedback. Optimal aDBS amplitude ranges were defined as follows: *Left Hemisphere*: minimum 0.8 mA, maximum 1.2 mA, pause amplitude 1 mA; *Right hemisphere*: minimum 0.9 mA, maximum 1.4 mA, pause amplitude 1.2 mA. **(b)** Power spectral density of right and left STN activity (BrainSense™ Survey) used to identify frequency bands for longitudinal monitoring. Low-beta activity (16.6 Hz) was identified as the most informative band within the allowable spectral range for adaptive therapy (7.8–30 Hz) in both hemispheres. **(c)** Average traces (median ± s.e.m.) of LFP band power for the right and left STN (red and gray, respectively), time-normalized to a one-day period, and aligned with DBS stimulation amplitudes. Traces are shown across three phases: prior to adaptive therapy (left, phase 1), immediately after aDBS setup (middle, phase 2), and after several optimization sessions (right, phase 5). Average band-power traces progressively flattened for both hemispheres, indicating reduced fluctuations, which were accompanied by participant-reported improvements in sleep, mobility, and overall well-being. **(d)** (*Top*) Bar plots illustrate the percentage of time spent in continuous (black) versus adaptive DBS (red) across successive temporal phases, from prior to aDBS setup (phase 1) to optimized adaptive therapy (phase 5), highlighting the gradual increase in aDBS usage by the participant. Interruptions of adaptive therapy during phases 2, 3, and 4 were primarily due to episodes of dyskinesia at specific times of the day, requiring reduction of stimulation amplitudes to levels not reached by aDBS. During phase 5, persistent gait-related difficulties, described by the participant as a sensation of heavy legs and increased effort during walking, prompted discontinuation of adaptive therapy and manual increases in stimulation amplitude during prolonged walking. (*Bottom*) MDS-UPDRS III scores assessed ON L-DOPA during each in-clinic optimization session. Assessment during session 2 was performed OFF L-DOPA (score: 19/132) but is not shown in the figure for clarity. **(e)** Illustrative example of aDBS behavior during a full-day in-lab assessment, showing DBS amplitude adaptations tracking L-DOPA pharmacokinetics and compensating for modulations in low-beta power. STN LFP spectrogram (BrainSense™ Streaming) is aligned with DBS stimulation amplitudes for the right and left STN over the time window from 10 h to 15 h.

Despite initial clinical benefits from DBS therapy, participants progressively experienced a worsening of cardinal motor symptoms over time, which was managed through iterative adjustments of stimulation parameters. However, these adjustments remained suboptimal for mitigating locomotor deficits and, in some cases, aggravated them. Three participants (P1–P3) were treated with continuous DBS (cDBS). One participant (P4) was treated with beta-based aDBS for 3 months and reported overall satisfaction with the therapy (Extended Data Fig. 7), although persistent gait-related complaints compelled him to override adaptive therapy and manually increase stimulation amplitudes during walking.

We thus investigated whether activity-dependent adjustments in DBS amplitude differentially improved locomotor deficits versus cardinal motor symptoms. For each participant, we characterized the most disabling gait impairments during the eligibility session using clinical gait assessments, MDS-UPDRS III scores, neurologist reports and patient interviews. We then identified optimized DBS amplitudes that mitigated these gait deficits relative to cardinal motor symptoms (Fig. 5e and Extended Data Table 1).

Participant P1 presented with pronounced upper-limb rigidity that was managed with high DBS amplitudes; however, these parameters interfered with right-leg function during walking. Reducing DBS amplitude mitigated these interferences. Participant P2 exhibited severe FOG when low in L-DOPA, especially when turning, passing through doors and avoiding obstacles. Increasing DBS amplitude mitigated these difficulties, but exacerbated neck rigidity and impaired speech. Participant P3 exhibited prominent fluctuations-related dystonic movements affecting the right leg that disrupted walking. Reducing DBS amplitude immediately alleviated these deficits, but worsened upper-limb rigidity. Participant P3 also experienced FOG when low in L-DOPA, which was exacerbated by high-amplitude DBS; during experimental sessions, FOG improved following transient reductions in DBS amplitudes. Participant P4 presented with right-leg weakness and reduced walking endurance, described as a sensation of heavy legs and increased effort during prolonged ambulation. These gait-related deficits improved with increased DBS amplitudes, at the expense of dyskinesia.

**Fig. 6:**
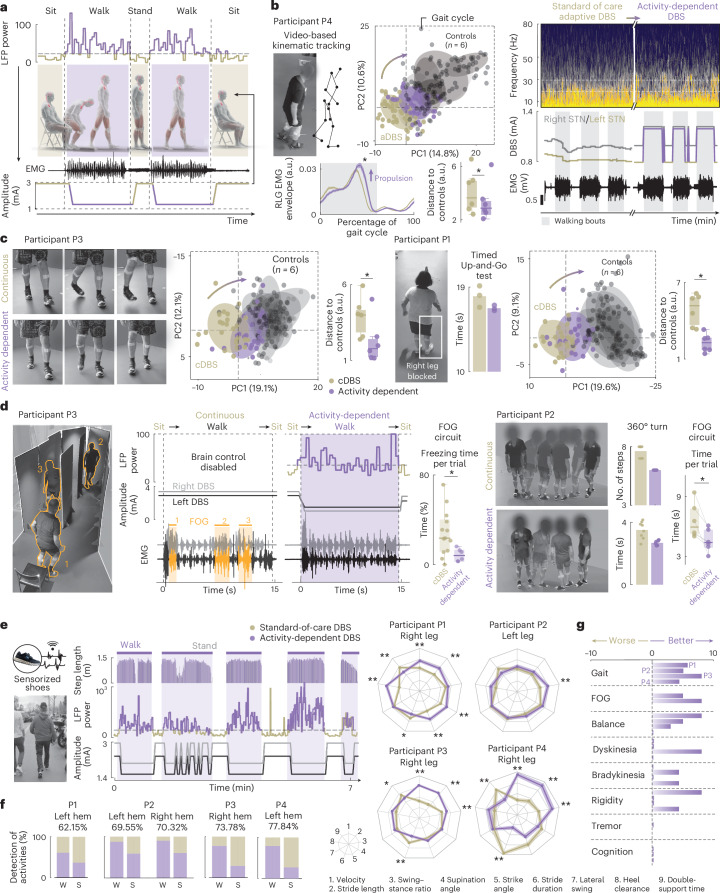
Patient-specific locomotor improvements with activity-dependent DBS. **a**, Illustrative example of activity-dependent DBS adaptations during a task alternating between sitting, standing and walking. LFP power in the selected spectral band is monitored in real time to trigger transitions between DBS amplitudes optimized for cardinal motor symptoms versus locomotor impairments. **b**, Improvements in gait performance mediated by activity-dependent DBS in participant P4, previously treated with beta-based aDBS for 3 months (Extended Data Fig. 7). P4 reported overall satisfaction with adaptive therapy, including improved sleep quality and mobility. However, he continued to experience right-leg weakness and reduced walking endurance, described as a sensation of heavy legs and increased effort during prolonged ambulation. Left: continuous video-based three-dimensional kinematic tracking (including leg, arm and trunk kinematics) parameterizes each gait cycle into a high-dimensional feature space. The two-dimensional PCA space explaining the largest variance (first two principal components (PCs); ~25%) illustrates changes in gait quality under standard-of-care DBS (aDBS; yellow) and activity-dependent DBS (purple), relative to healthy controls (*n* = 6), during a continuous walking task comprising series of back-and-forth 8-m walks with turns around a cone. Each dot represents one gait cycle. Boxplots quantify differences in distance between each DBS condition and controls (two-sided Wilcoxon signed-rank test, *P* = 0.031, *r* = 0.90). Traces (median ± s.e.m.) of EMG envelopes (lateral gastrocnemius, most affected leg) confirm increased propulsion underlying these changes. Feature contributions to each PC are detailed in Extended Data Fig. 8c. PC1 primarily reflects upper-limb features, reflecting improvements in arm swing, whereas PC2 captures lower-limb and stability-related features (heel, tibia and center-of-mass kinematics), corresponding to improvements in walking speed and gait stability. Right: illustrative example of spectrograms aligned to DBS amplitudes and leg EMG traces (vastus medialis) under aDBS and activity-dependent DBS during three task repetitions, highlighting distinct adaptation dynamics. Sustained full-day dynamics are shown in Extended Data Fig. 9. **c**, Improvements in gait patterns mediated by activity-dependent DBS in participants P3 (left) and P1 (right), both previously treated with continuous DBS. Representative screenshots illustrate the most debilitating locomotor deficits (P3, right-leg dystonia under high L-DOPA; P1, impaired gait fluidity due to excessive right-leg blockage and reduced flexion). PCA of gait patterns under standard-of-care DBS (cDBS, yellow) and activity-dependent DBS (purple) shows changes relative to healthy controls (*n* = 6). Features contributing to the PCs primarily reflected gait timing and trunk kinematics in P1, corresponding to improved symmetry and fluidity during walking, and trunk and leg movements in P3, consistent with reduced right-leg dystonia. Feature contributions are provided in Extended Data Fig. 8c. Boxplots quantify differences in distance between each DBS condition and controls (two-sided Wilcoxon signed-rank test: *P* = 0.031, *r* = 0.90, both for P1 and P3). Time to complete three repetitions of the Timed Up-and-Go task is reported for P1. **d**, Improvements in FOG mediated by activity-dependent DBS in participants P3 (left) and P2 (right). Left: chronophotography of a freezing-of-gait circuit task, which involves navigating a narrow 8-shaped corridor, in participant P3. In the low L-DOPA condition, multiple FOG episodes were observed, including during sit-to-stand transitions and at recurrent locations along the circuit. Activity-dependent reductions in DBS amplitude mitigated the occurrence of FOG and improved performance, as shown by representative EMG traces. Boxplot quantifies improvements across repeated trials (P2 *n* = 13 under cDBS and *n* = 6 under activity-dependent DBS, two-sided Wilcoxon rank-sum test, *W* = 155, *P* = 0.028). Right: chronophotography of a 360° turning task in participant P2 under standard-of-care DBS (cDBS) and activity-dependent DBS. Bar plots quantify reductions in FOG (mean and individual data points). Boxplots show reduced time to complete a dedicated FOG circuit with several freezing zones under activity-dependent DBS (1 of 6 exhibited FOG) versus cDBS (6 of 6 exhibited FOG) (two-sided Wilcoxon signed-rank test *W* = 21, *P* = 0.03, see also Extended Data Fig. 8e). **e**, Left: out-of-laboratory assessments confirmed sustained improvements in gait patterns (P3). Panels show step-length trajectories recorded using sensorized shoes, together with LFP band power used to trigger DBS amplitude adaptations. Temporal parameters of the adaptive algorithm (average duration, blanking period and onset delay) were tuned to maximize stability of adaptations (traces are shown in Supplementary Fig. 5). Right: radar plots show kinematic gait features across gait cycles (mean ± s.e.m. normalized to the 25% and 75% quantile of the combined distribution, *n* gait cycles >120 for all participants and conditions) from the most affected leg, illustrating improvements in gait quality under activity-dependent DBS. Stars indicate significant differences (two-sided unpaired Welch’s *t*-test with Bonferroni correction for multiple comparisons; see Supplementary Table 7 for full statistical results). In participant P2, out-of-laboratory testing confirmed preserved efficacy for reducing FOG (Extended Data Fig. 8e) and improving ambulation. **f**, Performance in detecting each activity during out-of-laboratory assessments using the on-device threshold-crossing implementation of activity-dependent DBS (duration of activity-dependent DBS usage: P1, 17 min; P2, 22 min; P3, 37 min; P4, 41 min). Bar plots show the proportion of correctly or incorrectly classified activities. Titles indicate wF1-score. **g**, Self-reported assessments during out-of-laboratory sessions confirmed perceived improvements in overall motor function, primarily driven by gait-related benefits without worsening of cardinal motor symptoms. *P < 0.05, **P < 0.01. RLG, right lateral gastrocnemius muscle; hem, hemisphere; S, stationary; W, walking.

**Extended Data Fig. 8:**
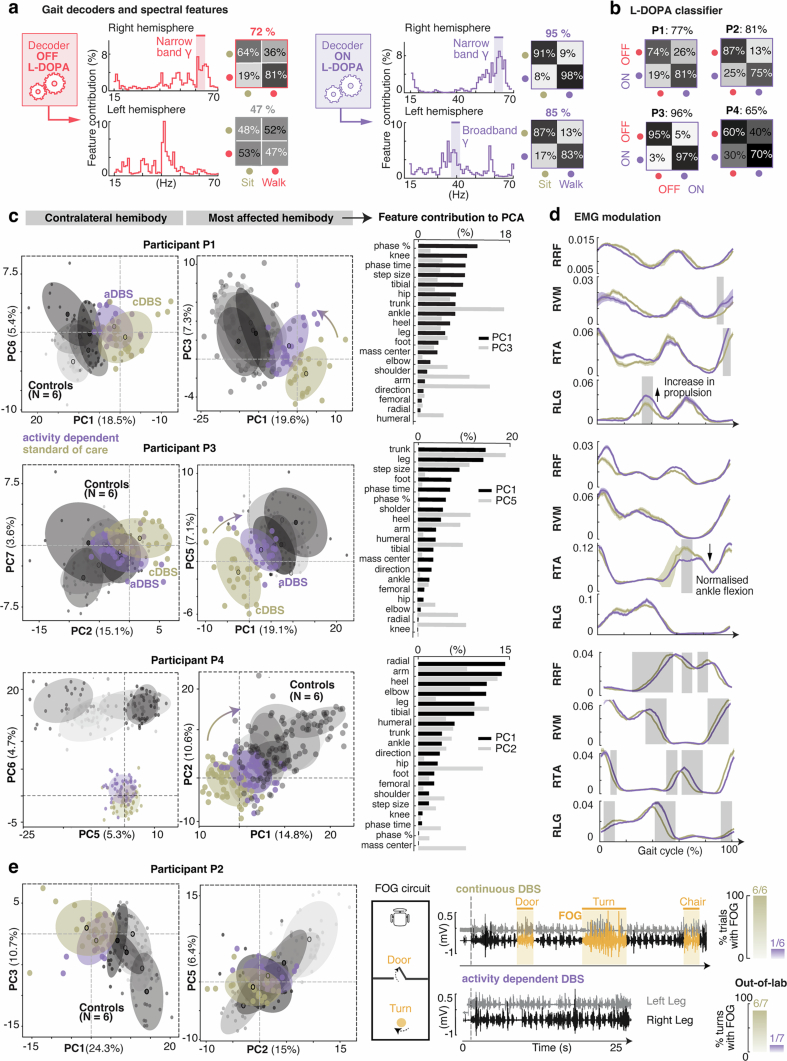
Participant-specific spectral features and improvements in most bothersome locomotor deficits with activity-dependent DBS. **(a)** Traces of spectral feature contributions to each gait decoder and corresponding confusion matrices illustrating predictive performance (participant P3). The optimal frequency band for each hemisphere (shaded red or purple) was selected as the most informative during online assessments and iteratively refined based on real-time decoding performance. When decoding performance from one hemisphere was insufficient (as indicated by the confusion matrices), a unilateral control signal was used to modulate DBS amplitudes bilaterally (see Extended Data Table 1). Broadband and narrow gamma bands were ultimately selected for the left and right hemispheres, respectively. **(b)** Cross-validation classification performance for the L-DOPA classifier in all four participants. **(c)** Gait modulations in participants with gait abnormalities. (*Left*) PCA-based kinematic analysis, computed over a comprehensive set of parameters derived from video-based tracking of whole-body walking patterns, demonstrates therapy-associated gait improvements across the three participants exhibiting gait abnormalities (P1: blocked right leg; P3: right leg dystonia; P4: leg weakness and limited endurance). The two principal components that best capture changes under activity-dependent DBS (purple) relative to standard-of-care DBS (P1 and P3: cDBS; P4: beta-based aDBS) are shown, selected based on the Fisher score (see Methods). Under activity-dependent DBS, gait patterns shift closer to those of healthy controls, indicating improved gait quality and reduced locomotor disturbances. (*Right*) Kinematic features contributing to the PC space of the most affected hemibody. Features are grouped into biomechanical ‘domains’ (for example, heel, ankle, knee, and hip joint, timing and proportion of gait phases). The relative contribution (%) of these features confirms predominant involvement of kinematic parameters related to each participant’s reported bothersome symptoms. **(d)** Traces of EMG envelopes (median ± s.e.m.) from multiple leg muscles recorded from the most affected hemibody, highlight DBS-mediated changes in muscle activation across the gait cycle. Significant time windows are represented by shaded gray areas (unpaired two-sided t-tests implemented in Statistical Parametric Mapping SPM1D, with Bonferroni correction for multiple comparisons). These modulations in leg muscle activity illustrate how improvements in whole-body gait patterns emerge from enhanced control of the most affected limb. **(e)** Gait modulations in a participant exhibiting predominant freezing of gait (P2). (*Left*) PCA analyses revealed minor differences in kinematic patterns between standard-of-care DBS and activity-dependent DBS in this participant, consistent with the absence of stepping abnormalities. (*Right*) A customized FOG circuit incorporating multiple freezing zones (sit-to-stand transitions, doorways, and turns) elicited freezing of gait in participant P2. Representative raw EMG traces from the tibialis anterior (TA) are shown for one repetition of the circuit performed under cDBS and under activity-dependent DBS. The timing and duration of FOG episodes were quantified from bilateral EMG activity patterns (statistics shown in Fig. 6d). Barplots quantify the number of freezing episodes during this task, and were confirmed in out-of-laboratory settings. cDBS: continuous DBS, aDBS: adaptive DBS.

**Extended Data Fig. 9:**
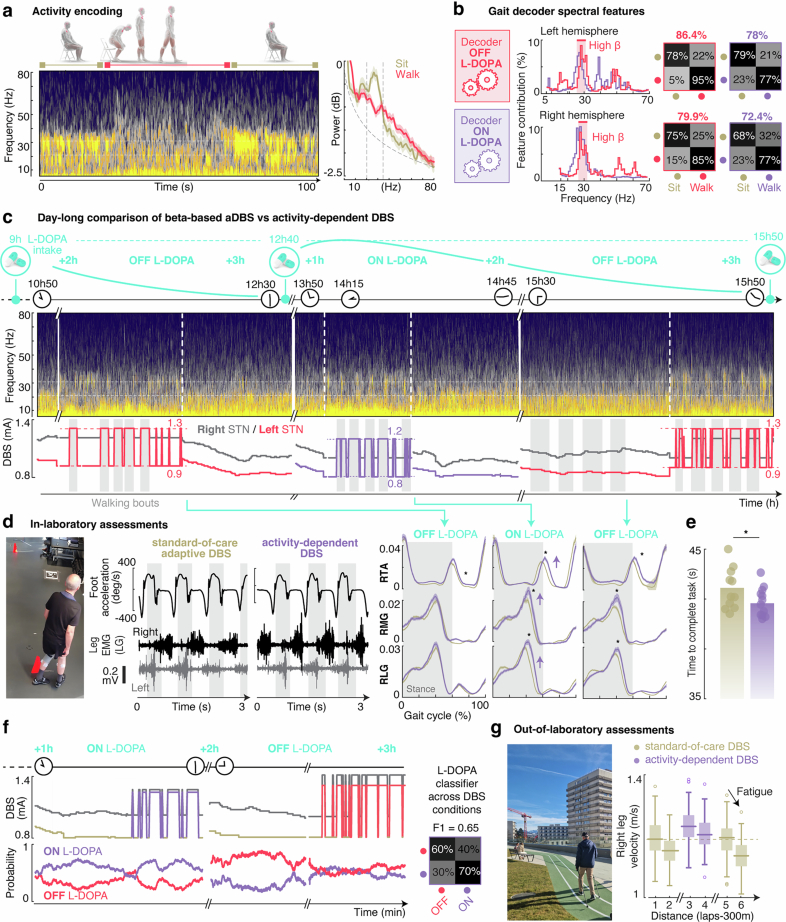
Comparison of beta-based adaptive DBS versus activity-dependent DBS (participant P4). **(a)** (*Left*) Illustrative example of LFP spectrogram during transitions between sitting, standing, and walking in participant P4. (*Right*) Power spectral density across these activities illustrates marked modulations in high-beta and gamma band power. **(b)** (*Left*) Spectral features contributing to gait decoders in the OFF- and ON-L-DOPA conditions (red and purple, respectively) confirm the relevance of high-beta and low-gamma bands for discriminating locomotor states in each hemisphere. (*Right*) Confusion matrices confirm accurate decoding of these states in each condition. **(c)** Day-long comparison of beta-based aDBS versus activity-dependent DBS across medication cycles. STN LFP spectrogram aligned with DBS stimulation amplitudes for the right and left STN over the time window from 10 h to 15 h, during which the participant performed repeated locomotor tasks involving alternations between sitting, walking, and turning around a cone. Activity-dependent DBS amplitudes were defined to optimally address gait deficits (heavy legs and increased effort during walking) under each medication condition. These amplitudes matched those reached by converging beta-based aDBS, which modulated stimulation in relation to L-DOPA pharmacokinetics within the clinically defined aDBS ranges (*Left Hemisphere*: minimum 0.8 mA, maximum 1.2 mA, pause amplitude 1 mA; *Right hemisphere*: minimum 0.9 mA, maximum 1.4 mA, pause amplitude 1.2 mA). However, the slower dynamics of beta-based aDBS were insufficient to accommodate the rapid, activity-dependent changes required during locomotion. **(d)** In-lab assessments of motor quality and performance. Illustrative examples of right-foot acceleration (most affected side) aligned to bilateral leg EMG traces (LG, lateral gastrocnemius) during three gait cycles under standard-of-care aDBS (left) and activity-dependent DBS (right). Average EMG envelopes (median ± s.e.m.) are shown for three distal ankle muscles involved in propulsion and foot clearance (MG, medial gastrocnemius; LG, lateral gastrocnemius; TA, tibialis anterior) under each DBS condition. Traces are shown at three time points over the course of the session, highlighting improvements in leg muscle activation across L-DOPA cycles. Stars indicate significant differences across the two conditions (p < 0.05, unpaired two-sided t-tests implemented in Statistical Parametric Mapping SPM1D, with Bonferroni correction for multiple comparisons). **(e)** Cross-session quantification of task completion time reveals consistent improvements in walking speed under activity-dependent DBS. Each dot represents one series of three consecutive back-and-forth 8-m walks with turns around a cone (N = 12 series per DBS condition; p = 0.0425, two-sided Wilcoxon signed-rank test W = 65). **(f)** Illustrative examples of pseudo-online implementation of the L-DOPA condition classifier under low- and high-L-DOPA states, across beta-based aDBS and activity-dependent DBS stimulation conditions. Transitions exhibited slight decreases in performance but maintained sufficient accuracy to guide therapy. **(g)** Out-of-laboratory assessments during continuous ambulation around a 300-m track. Three blocks were completed, each comprising two laps. Blocks were separated by 1-min rest periods and alternated between standard-of-care (beta-based) aDBS and activity-dependent DBS. Boxplots depict walking velocity of the most affected leg (number of gait cycles per lap: 123 lap1, 122 lap2, 123 lap3, 122 lap4, 125 lap5, 125 lap6). Across blocks, the second lap was consistently slower, reflecting progressive fatigue accumulation. Activity-dependent DBS increased walking speed during the first lap and attenuated the decline observed in the second lap, indicating improved endurance relative to standard-of-care aDBS. aDBS: adaptive DBS.

For each participant, we used our decoding pipeline to extract optimal spectral features. These features primarily reflected activity-dependent alpha synchronization in P1, gamma synchronization in P2 and P3, and high-beta desynchronization in P4 (Fig. 5d, Extended Data Figs. 8a and 9b, and Extended Data Table 1). We then implemented activity-dependent DBS protocols that adjusted stimulation amplitude in real time based on modulations of these features (Fig. 6a).

We evaluated the preliminary safety and efficacy of these protocols in well-controlled laboratory settings over two consecutive full-day sessions (Fig. 6a–d and Extended Data Fig. 8c–e). Participants and clinical evaluators were blinded to the stimulation condition in all applicable experiments (Methods). Activity-dependent DBS adaptations mitigated locomotor deficits and improved gait quality during walking, while preserving efficacy for cardinal motor symptoms during sitting and standing (Fig. 6b–d and Supplementary Video 3). Participant P1 showed improved leg agility (Fig. 6c and Extended Data Fig. 8c,d), P2 exhibited reduced FOG (Fig. 6d and Extended Data Fig. 8e), P3 displayed alleviation of leg dystonia and FOG (Fig. 6c,d and Extended Data Fig. 8c,d), and P4 showed increased step length, arm swing and walking endurance, along with reduced camptocormia (Fig. 6b and Extended Data Fig. 9). Gait improvements were quantified using principal component analysis (PCA)-based kinematic and EMG analyses, whereas improvements in FOG were assessed through freezing duration metrics. No adverse effects were reported.

We finally validated the robustness of activity-dependent DBS in out-of-laboratory conditions (Fig. 6e,f and Supplementary Fig. 5). Kinematic assessments from wearable sensors, together with questionnaires capturing participants’ subjective experience, confirmed the superiority of activity-dependent DBS over standard-of-care protocols (Fig. 6e,g).

## Discussion

We exposed physiological principles by which the STN encodes locomotor activities across motor fluctuations in people with PD. This understanding guided the development of a modular decoding framework capable of predicting locomotor activities in real time from STN LFPs, regardless of L-DOPA fluctuations and changes in DBS parameters. We leveraged this framework to implement personalized, activity-dependent DBS protocols on an investigational neurostimulation platform with closed-loop capabilities. In a proof-of-concept clinical study in four individuals with refractory gait impairments, activity-dependent DBS improved locomotor deficits in real-world settings.

Conventional DBS therapies are primarily optimized to alleviate rigidity, bradykinesia or tremor. However, locomotor deficits respond to DBS differently from cardinal motor symptoms, a distinction long recognized in clinical practice. This discrepancy is often addressed manually through parameter reprogramming when gait deteriorates, to accommodate the physiological demands of standing or walking. Optimal management of all symptoms thus requires distinct DBS configurations tailored to each set of impairments. Switching between these configurations must operate at fast, behavior-linked dynamics. Recently commercialized aDBS therapies relying on beta biomarkers provide increased personalization in response to medication-related fluctuations, but operate over slow timescales and remain largely agnostic to behavioral context.

Because locomotor deficits also vary with the motor demands of daily activities and fluctuating physiology, effective management is contingent on real-time detection of mobility requirements across pharmacokinetic cycles. Our analyses in a cohort of 35 individuals with PD revealed that the distinct muscle activation demands underlying many of these activities are encoded in STN dynamics. These findings confirm that the STN encodes movement vigor during whole-body locomotion, consistent with prior reports on upper-limb movements^52,53^ and isolated leg muscle activation^21^, and extend this principle across therapeutic conditions. Indeed, we found that locomotor encoding was retained under both L-DOPA and STN DBS, despite profound and opposing therapy-specific alterations that primarily affected low-beta and gamma frequency bands^47,48,54^. However, these alterations degraded neural decoder performance across therapeutic conditions.

We addressed this challenge with a modular framework composed of therapy-specific decoders operating in parallel. This modular architecture decomposed a complex, multi-objective control problem into smaller, therapy-specific sub-problems, which achieved reliable prediction of locomotor activities in real time even in the presence of L-DOPA fluctuations and DBS adjustments. To disentangle neural modulations associated with locomotor activities from therapy-induced alterations in the same STN LFP signals, each module was tuned to operate over a distinct timescale, corresponding to the temporal dynamics of either locomotion, L-DOPA fluctuations or DBS-induced changes.

This modular architecture is well suited for medical applications that impose strict technical and clinical constraints^55,56^. Real-time algorithms embedded in implantable devices must be computationally efficient, easy to tune across individuals and trainable with limited amounts of data. Ideally, decoder parameters should also remain interpretable by healthcare professionals, empowering them to personalize therapies independently. Unlike large models requiring high computational resources and typically designed for offline rather than embedded operation^22,57^, our therapy-specific decoders relied only on a small number of free parameters and could be trained in minutes using a few gait sequences. All other hyperparameters were optimized offline using a genetic algorithm to ensure accuracy and transferability across patients and therapies. This modular design also permits the seamless integration of additional inputs as new modules, such as electrocorticography signals^34,58,59^ or data from wearable sensors^60^, thereby maximizing flexibility and scalability for multi-therapy control.

We leveraged the modular decoding framework to guide the implementation of activity-dependent DBS strategies targeting both locomotor deficits and cardinal motor symptoms in real time. We obtained proof of efficacy of these strategies in a clinical study involving four participants with persistent gait impairments. The identification of patient-specific spectral features that capture ongoing activities despite the confounding effects of L-DOPA and STN DBS was essential for delivering robust adaptations in stimulation parameters^61^. Our results showed that this identification required exploiting the rich spectral encoding of locomotor activities under each therapy, which spans multiple—and often overlapping—frequency bands across the entire LFP spectrum. This included frequencies beyond the beta band typically accessible in commercial devices^62^, exhibiting activity-related synchronization or desynchronization patterns.

We implemented these strategies on a commercially available neurostimulation platform with investigational features that not only allowed us to leverage the full spectral content of STN LFPs for advanced monitoring and control, but also enabled band-specific upregulation or downregulation of stimulation amplitude at fast update rates. These functionalities were pivotal to accommodate the diverse demands of locomotor impairments. Even though the four participants exhibited heterogeneous clinical profiles, activity-dependent adaptations of DBS parameters ameliorated the full spectrum of deficits. These results demonstrate the clinical feasibility of activity-dependent DBS therapies to address gait impairments, including deficits not adequately targeted by current state-of-the-art aDBS approaches.

Prior reports on aDBS identified gait deficits as one of the common unresolved impairments in patients who discontinue adaptive therapy^36,63^. Moreover, growing evidence from aDBS studies highlights the limitations of beta-band biomarkers^36,63^, questioning the broad applicability of this approach to all individuals. In line with these observations, participant P3 was tested under beta-based aDBS but lacked a prominent beta-band biomarker, resulting in substantial feedback variability and inconsistent stimulation adaptations despite iterative optimization^64^. After a few weeks of optimized therapy, P3 did not perceive overall benefit and elected to return to continuous DBS. In this context, our activity-dependent DBS framework provides a path toward adaptive gait therapy in patients who fail beta-based aDBS.

However, our implementation has limitations. Reliance on a single frequency band rendered control sensitive to mis-parameterization and biomarker instability, which limited robustness for long-term use in real-world conditions. Our modular decoding framework identified the spectral features best suited to operate under these constraints, which was critical to maintain control stability during our assessments. However, expanding feedback to more frequency bands or incorporating additional inputs beyond STN LFPs, such as electrocorticography or wearables, could enhance controllability and stability^35^. Moreover, in this study, decoders remained fixed once trained. Retraining will become necessary in longitudinal settings. Future implementations should incorporate online learning-based updating in our modular framework to better accommodate long-term changes.

An additional limitation is that adaptive control was restricted to stimulation amplitude. While these adaptations mitigated gait impairments in our four participants, future platforms may benefit from real-time adjustment of stimulation frequency and contact configuration. This would enable activity-dependent delivery of low-frequency stimulation via ventral contacts, guided by tractography, to better target gait-related pathways^65,66^. Finally, addressing the full spectrum of impairments will require the incorporation of complementary decoding and stimulation strategies focused on upper-limb symptoms^35,67^.

Our modular decoding framework offers a blueprint for next-generation neuromodulation therapies that dynamically adapt stimulation parameters to the behavioral context, mobility demands and fluctuating physiology of each individual with PD^68,69^. Realizing the full potential of activity-dependent adaptive DBS will require dedicated hardware and large-scale clinical trials to demonstrate long-term safety and efficacy in real-life settings^70^.

## Methods

### Study design

The objective of this study was to uncover the physiological principles by which the STN encodes daily mobility activities across motor fluctuations in people with PD, and to leverage this understanding to develop activity-dependent DBS therapies capable of alleviating both cardinal motor symptoms and locomotor deficits in real time.

The study was conducted in two parts. First, we characterized how locomotor activities are represented in STN dynamics and how these neural patterns are altered by L-DOPA and STN DBS. Assessments in 35 participants informed the development of neural decoders to guide the implementation of adaptive DBS strategies. Second, we conducted a feasibility clinical trial (AdapGAIT, ClinicalTrials.gov registration NCT06791902) using an investigational neurostimulation platform (Medtronic) to evaluate the safety and preliminary efficacy of activity-dependent DBS in four individuals with PD.

### Characterization of physiological principles and development of neural decoders (part 1)

Forty-one participants with PD were recruited. All participants were implanted with bilateral directional DBS leads (Sensight, Medtronic) and a Percept PC neurostimulator (Medtronic) implanted in the right chest. Recordings were performed within seven days following DBS implantation using the sensing capabilities of the neurostimulator. OFF-medication recordings were obtained after withdrawal from dopaminergic medication for at least 12 h. For ON conditions, stimulation amplitudes and medication dosages were maintained at clinically optimized levels as determined by the treating neurologist (Supplementary Table 1).

#### Participant inclusion and exclusion

Two participants were unable to complete the required motor tasks and were not recorded. Among the 39 participants recorded, 3 were excluded because of severe ECG-related artifacts affecting all LFP channels, and 1 because of the activation of a cycling stimulation mode that prevented consistent comparison across therapeutic conditions. A total of 35 participants were retained for analysis.

#### Participation to motor tasks

Not all 35 participants participated in all motor tasks. Participants were grouped based on the therapeutic conditions and motor tasks in which they were assessed (Supplementary Table 2). The first cohort included 21 participants who performed continuous walking tasks under two conditions: OFF therapy and the standard-of-care combination of STN DBS and L-DOPA. In this cohort, the first ten participants were additionally assessed under STN DBS alone and during progressive pharmacokinetic phases of L-DOPA absorption (0, 15, 30 and 45 min after medication intake). Two participants in this cohort did not require L-DOPA medication. The second cohort included 14 participants to characterize gait encoding under L-DOPA alone and varying DBS amplitudes. The first 11 participants performed walking tasks in two conditions: OFF therapy and L-DOPA only. Among these, eight participants also completed recordings under different DBS amplitudes. One participant in this cohort ended up not requiring L-DOPA medication at the time of assessments.

As a result, the number of participants retained for each condition was as follows: 19 were analyzed during walking tasks under standard therapeutic conditions (OFF-therapy and the standard-of-care combination of STN DBS and L-DOPA; group A); 9 were analyzed during walking tasks tracking the time-dependent effects of L-DOPA pharmacokinetics (0, 15, 30 and 45 min post-intake; group B); 15 and 10 participants were analyzed during walking tasks under STN DBS only and L-DOPA only, respectively (groups C and D); and 8 participants were analyzed under varying DBS amplitudes (group E). In addition, four participants performed walking tasks during manual toggling of DBS amplitude to evaluate the online robustness of neural decoders (group F).

No significant differences were observed in MDS-UPDRS III scores across subgroups, either in the OFF- or ON-therapy conditions (one-way ANOVA with Bonferroni post-hoc correction, *P* > 0.05). Mean scores were as follows: group A, OFF: 40 and ON: 14; group B, OFF: 33 and ON: 13; group C, OFF: 40 and ON: 18; group D, OFF: 38 and ON: 15; group E, OFF: 42 and ON: 16; and group F, OFF: 31 and ON: 9.

#### Ethics

All experiments were approved by the Ethical Committee of the Canton de Vaud, Switzerland (ref PB_2017-00064). Informed consent was obtained before each recording. The nature and possible consequences of the study were systematically explained to all participants. No financial compensation was provided.

### Feasibility clinical trial to assess activity-dependent adaptive DBS (part 2)

Eight individuals with advanced PD were screened for inclusion. All had been implanted with bilateral STN DBS leads (Sensight, Medtronic) and a Percept neurostimulator at least three months before screening and exhibited persistent gait impairments despite optimized standard therapy.

#### Inclusion and exclusion criteria

Participants were required to exhibit clinically relevant gait deficits, be cognitively able to comply with study procedures, and present neural signals suitable for decoding (that is, exhibiting movement-related modulations not dominated by artifacts). Additional criteria included tolerance to changes in DBS amplitude and provision of informed consent. Exclusion criteria included major psychiatric or neurocognitive disorders, planned changes in dopaminergic therapy, substance abuse, pregnancy or recent participation in another investigational study.

Of the eight screened participants, three were excluded due to insufficient gait impairment and one due to cognitive impairment. Four participants were enrolled in the study. Demographic and clinical data are detailed in Supplementary Table 3. At the time of enrollment, DBS parameters and levodopa regimens had been optimized by the participant’s treating neurologist to target the most disabling motor symptoms consistent with akinetic–rigid profiles. All participants provided informed consent to participate in the study, including consent for the reuse of images or materials derived from the study, and received compensation for their participation.

#### Clinical characteristics and baseline therapy

Three participants (P1–P3) were treated with continuous DBS, with a median stimulation amplitude of 2.9 mA across hemispheres. One participant (P4) had been treated with beta-based adaptive DBS for approximately three months, with parameters optimized across multiple clinical sessions (Extended Data Fig. 7). In its final configuration, stimulation amplitudes for P4 varied in relation to L-DOPA pharmacokinetics in clinically defined ranges (left hemisphere: minimum 0.8 mA, maximum 1.2 mA, pause amplitude 1.0 mA; right hemisphere: minimum 0.9 mA, maximum 1.4 mA, pause amplitude 1.2 mA). Although P4 reported overall satisfaction with aDBS, he reported persistent gait-related complaints.

#### Regulatory approval

The study was approved by the Swiss Ethics Committee (BASEC 2024-D0066, protocol number 10001378, approved on 21 November 2024) and the Swiss regulatory agency (SNCTP 000006185, protocol number 102731544, approved on 23 December 2024) and conducted in accordance with the Declaration of Helsinki. The study is registered on ClinicalTrials.gov (NCT06791902).

#### Study objectives

The primary objective was to assess safety and preliminary efficacy, evaluated through the occurrence of adverse events related to study procedures. Secondary objectives included assessment of gait performance using standardized clinical tests (Timed Up-and-Go, 10-m walk test), as well as quantitative kinematic and EMG analyses. FOG was evaluated using validated paradigms, including a dedicated freezing-of-gait circuit and 360° turning test.

No adverse events were observed during the study.

#### Healthy controls

To provide a reference for physiological gait, kinematic data were acquired from six healthy controls (three male, three female) under a separate approved protocol (ref. 2017-02112). No financial compensation was provided to these participants.

### DBS electrode localization

Lead localization was performed using the Lead-DBS MATLAB toolbox^71^, based on preoperative T1- and T2-weighted magnetic resonance imaging and postoperative computed tomography scans. Postoperative computed tomography images were linearly co-registered to preoperative magnetic resonance imaging using Advanced Normalization Tools^72^ with visual inspection and manual correction when necessary. Brain shift correction was applied as implemented in Lead-DBS.

All preoperative volumes were normalized to ICBM 2009b NLIN asymmetric space^73^ using Advanced Normalization Tools SyN diffeomorphic mapping^72^ (preset: low variance default with subcortical refinement). DBS contacts were automatically reconstructed using the PaCER method^74^ and individually verified.

Of all recorded hemispheres, seven were excluded from analysis based on anatomical reconstructions, which raised concerns that the selected contacts were not fully located in the motor subregion of the STN (Supplementary Fig. 1). This anatomical uncertainty was corroborated by the absence of any detectable signal modulation across the entire spectrum of STN LFPs or under different therapeutic conditions throughout the experiments.

### LFP recordings

LFPs were recorded using the sensing capabilities of the Percept neurostimulator in BrainSense Streaming mode (sampling rate 250 Hz), which permits recording from a single bipolar contact pair per hemisphere. For each participant, the contact pair adjacent to the clinically active stimulation electrode was selected. When stimulation was delivered through contacts incompatible with sensing (contacts 0 or 3), the nearest available contact pair was used.

Synchronization with external systems was achieved by delivering brief DBS bursts (using standard stimulation parameters) at the start and end of each recording. These pulses generated identifiable artifacts in a chest EMG sensor placed near the implantable pulse generator, enabling precise alignment across modalities.

### Definition of LFP power frequency bands

The identification of power frequency bands was personalized for each patient. Low-beta, high-beta, and gamma bands were identified using an unbiased algorithm that fits the PSD of each patient as a combination of an aperiodic (1/*f*) and a mix of Gaussian components^42^. The algorithm iteratively optimized the fit and found the most appropriate number of Gaussian components and their parameters (mean and s.d.) to model the original spectrum with a given accuracy.

Because frequency bands may differ or shift between rest and movement, and specific bands may only emerge during movement (such as broadband gamma), we run the fitting algorithm on the PSD computed on a complete set of recordings, including both rest and movement periods. Identified Gaussian components in the range of 10–20 Hz were labeled as ‘low-beta,’ those in the range of 20–35 Hz were labeled as ‘high-beta’, and those above 35 Hz as gamma.

Across 35 participants (63 hemispheres), a low-beta band was identified in 54 hemispheres (mean range 12.9–19.5 Hz), a high-beta band in 58 (21.6–31.6 Hz) and a gamma band in 56 (37.7–68.8 Hz) (Supplementary Fig. 2). Subsequent analyses of activity-dependent power modulations were restricted to hemispheres in which the corresponding band was detected.

### Removal of ECG-related artifacts from LFP

For participants whose LFP recordings exhibited ECG-related artifacts (*n* = 10), a denoising algorithm based on singular value decomposition (SVD)^75^ was applied to isolate and recover the underlying neural signal.

Artifact timing was identified from the EMG sensor over the chest used for synchronization. The EMG signal was *z*-scored, and R-peaks were detected using the findpeaks function (MATLAB R2024a) with a minimum amplitude of 2.5 s.d. above baseline and a minimum inter-peak interval of 500 ms. Detected R-peaks were projected onto the synchronized LFP signal to isolate QRS complexes (the QRS complex corresponds to ventricular depolarization and consists of the Q, R and S deflections, with the R peak typically used as a robust temporal marker for heartbeat detection).

For each peak, a 400-ms window (±200 ms) was extracted and decomposed using SVD. The ECG artifact was reconstructed from the two components with the largest eigenvalues and subtracted from the original LFP signal. A final offset correction was applied by minimizing the squared error between the raw signal and the reconstructed artifact.

When ECG peaks were not detectable in the chest EMG signal, they were identified directly from the LFP signal, and the same denoising procedure was applied.

### Locomotor tasks

#### Walking tasks

Patients were instructed to stand for 3 s before initiating a sustained bout of walking (in a straight line) at their comfortable speed. When arriving at the end of the bout, patients were instructed to stop and stand for 3 s, before doing a U-turn and starting another walking bout.

#### Obstacle task

Patients were asked to walk at their natural speed in a straight line (without any marks on the floor). An obstacle (height 10.5 cm, length 39.5 cm) was placed on their path. They were requested to stride over it and continue walking normally until the end of the bout.

### Biomechanical recordings during locomotor tasks

#### Kinematics (in-laboratory)

High-fidelity three-dimensional joint kinematics (foot, ankle, knee and hip) were recorded using an optoelectronic motion capture system (Vicon). Recordings were complemented with bilateral triaxial inertial measurement units (IMUs; Delsys) attached to the shoes, capturing gyroscope signals from both feet (148 Hz).

#### Kinematics (out-of-laboratory)

Walking activity was recorded using sensorized shoes (NUSHU, Magnes) equipped with insole IMUs. Embedded algorithms automatically detected gait events and computed per-cycle parameters including step length, step height and maximal foot acceleration.

#### Electromyographic signals

EMG signals were recorded using wireless EMG sensors operating at 1.5 kHz (Delsys). Sensors were placed bilaterally, on agonist and antagonist muscles of the ankle joint (tibialis anterior, medial gastrocnemius, lateral gastrocnemius), knee joint (vastus medialis, semitendinosus) and hip joint (rectus femoris). An additional EMG sensor was placed on the chest for synchronization purposes. All recordings were synchronized using dedicated trigger signals.

### Identification of gait events during walking tasks and definition of locomotor states

#### Bilateral gait events

We computed periods of walking and turning, along with toe-off and heel-strike events, automatically from gyroscope signals from the right and left feet using a graphical tool (open-access: http://github.com/matteonocilli/GAIT_tool)^76^.

#### Locomotor states

Using the aforementioned gait events, we categorized each time-point of the recordings into five discrete locomotor states, labeled as ‘sitting’, ‘standing’, ‘continuous walking’ and ‘turning’. This state-machine description of gait was further used for developing gait-state decoders and computing state-related analyses of power.

### Analyses of gait quality during locomotor tasks

A walking sequence was defined as a bout between consecutive stops or turns, and a gait cycle as the interval between successive foot strikes of the same leg.

#### Gait parameters

Walking speed was computed as distance divided by duration for each sequence (m s^−1^), and step length as distance divided by the number of steps (m). Turning duration was defined as the time required to complete each turn.

#### Muscle activation

EMG envelopes were obtained by bandpass filtering raw signals (20–500 Hz, fourth-order zero-lag Butterworth), full-wave rectification and low-pass filtering (7 Hz). Muscle activation was quantified as the area under the envelope.

#### Therapy-related changes

For each participant, median values of gait parameters were computed across therapeutic conditions.

### Spectral and power analyses during walking experiments

#### Spectrograms

Time–frequency representations of LFP signals were computed using multitaper decomposition (Chronux; http://chronux.org/) over 5–125 Hz (3 tapers, 1-s window, 50-ms overlap).

#### Average scalograms

Scalograms were computed for full walking sequences (from 2 s before first heel-off to 2 s after last foot strike) and per gait cycle (between consecutive foot strikes) using continuous wavelet decomposition (Morlet wavelets, MATLAB). Power was normalized to standing periods. Walking sequences were pooled by leading foot, time-normalized (5,000 points for standing phases, 1,500 between foot strikes), and averaged. Gait cycles were pooled, interpolated and averaged.

#### Band power across locomotor states

For each state, PSDs were computed from concatenated LFP signals using Welch’s method (1 Hz resolution, 50% overlap). Band power was defined as the area under the curve within band limits after removal of the aperiodic (1/*f*) component, and normalized by bandwidth to enable cross-patient comparisons.

#### Correlation between band power and gait quality

Band power and gait metrics (for example, walking speed) were computed per walking sequence and related using Spearman correlation (*n* = 10 repetitions).

#### Gait-cycle power comparisons

Band-specific power was computed from bandpass-filtered LFP signals (fourth-order Butterworth; band limits defined by spectral fitting) and normalized to standing periods.

To compare band-power changes between ‘normal’ steps and ‘obstacle-avoidance’ steps, we computed the median power for each repetition of each state (*n* = 20 sequences).

Electrophysiological analyses were performed at the hemisphere level. This distinction is standard in the field, as Parkinson’s symptoms are typically lateralized and right–left differences often translate into distinct electrophysiological biomarkers. Indeed, we observed clear within-patient asymmetries between hemispheres. These differences are further influenced by variations in electrode placement. By contrast, behavioral assessments (including quantification of kinematic and muscle activity patterns) were computed per patient.

### Identification of artifacted LFP channels during gait

Detection of gait-related artifacts is challenging because of temporal alignment with physiological signals and spectral spread beyond low frequencies.

To identify LFP channels corrupted by gait-related artifacts, we followed the same process as previously described^26^. Rather than aiming to identify artifacts in the time-domain, we reasoned that corrupted channels would exhibit important differences in the aperiodic (1/*f*) component of the PSD between rest and walking. This component captures the overall baseline power across the spectrum and should not change importantly over consecutive trials.

For each patient, we applied the fitting algorithm^42^ to the PSD of two separate recordings, one at rest (sitting) and one during walking (from the same session). We then compared their aperiodic (1/*f*) component. We computed the root mean square error in the range of interest (10–90 Hz) and ensured that walking did not induce an increase in 1/*f* power >50% compared to that sitting (difference 1.76 dB). All channels that exceeded that value were considered as corrupted in the region of interest and discarded for further analyses or decoding purposes. Visual inspection of the spectrogram confirmed that channels labeled as ‘artifacted’ exhibited periodic low-frequency spikes that corrupted the spectrogram and spread to higher frequencies. Retained channels were also verified by visual inspection of their spectrogram.

This conservative approach may discard channels that still contain usable information (for example, at higher frequencies), but ensures robustness of subsequent analyses. Overall, 2 of 63 hemispheres were excluded because of movement-related artifacts that biased decoding.

### Neural decoding framework

We used a custom-built Python software (https://github.com/dbdq/neurodecode) for real-time decoding of locomotor activities from raw STN LFPs. The decoder was based on Random Forest algorithms and implemented using the Python scikit-learn library (hyperparameters: 1,000 trees, 5 maximum depth and balanced subsampling). Testing performance was computed using Monte Carlo cross-validation over eight repetitions (20% of data for testing, 80% for training) in the epoch windows.

Subsequently, decoding performances were validated in a pseudo-online manner by replaying the data in chronological order, mimicking the data reading from the temporary buffer of the implantable pulse generator used in the online LFP acquisition. Computations run on a Windows 10 at ~15 Hz (one prediction every ~60 ms) as defined by computer performance. Probability traces for each class were then smoothed (alpha = 0.1) to prevent abrupt noisy peaks or artifacts. Quantification of performance is reported as the average wF1-score per class. Feature importance vectors (contribution of each frequency band to the overall prediction) were calculated for each channel from the trained model, and normalized (sum of all contributions equal to 1).

### Genetic algorithms to optimize decoding parameters across therapeutic conditions

Decoder performance depends critically on the selection of spatial, spectral and temporal features from raw neural signals. To avoid bias introduced by manual feature selection and improve robustness to shifts in neural distributions, we implemented a genetic algorithm-based framework to automate feature engineering (Extended Data Fig. 5).

We used the Nondominated Sorting Genetic Algorithm II, which optimizes candidate decoders using a multi-objective strategy and identifies a Pareto front of nondominated solutions that balance competing objectives. Candidate decoders were parameterized by feature sets (‘genes’) and evolved over successive generations through mutation, crossover and selection. Selection was based on nondominance ranking and diversity, enabling efficient exploration of the feature space while maintaining high-performing solutions.

This framework was first applied to develop decoders of locomotor states (sit, stand, walk) across participants, and subsequently to classifiers of L-DOPA and DBS conditions. The algorithm was run for ≥20 generations with a population of 50 decoders, optimizing: (1) epoch window; (2) frequency range; and (3) temporal window length for feature extraction. Window length emerged as the dominant parameter, followed by frequency bounds. Optimal values were 1–1.2 s for locomotor decoding and 18–20 s for L-DOPA classification, consistent with the underlying temporal dynamics of gait cycles (~1 s) and pharmacokinetics (minutes). These parameters were used in subsequent analyses.

We note that cross-patient hyperparameter computation was performed using data from all individuals, rather than with a leave-one-patient-out procedure. As a result, we cannot exclude leakage of patient-specific idiosyncrasies. However, because our objective was to optimize hyperparameters for the current patient pool rather than for unseen patients, this limitation is acceptable. The genetic algorithm was configured to maximize expected performance in the cohort. Allowing each fold’s training set to include data from all participants increased the signal-to-noise ratio and reduced the undue influence of outliers. Future work may extend this approach to pooled optimization strategies that ensure patient-level generalization and derive values that translate to new participants.

### Decoding algorithms of locomotor activities

Decoders were trained to classify ‘sit’, ‘stand’ and ‘walk’ activities using LFP signals from contacts not contaminated by movement-related artifacts. Analyses were restricted to frequencies >10 Hz (10–100 Hz for all patients) to retain physiological signals. Epoch duration was set to 1.5 s relative to class onset. ‘Standing’ epochs spanned from ≥2 s before the first step to after the last step; for ‘walk’, the first half of each walking sequence was used; ‘sit’ corresponded to seated rest.

To avoid data leakage arising from autocorrelation in brain signal, we defined the training and testing dataset in three steps: (1) bouts associated with each locomotor activity (sitting, standing, walking) were defined from EMG and kinematic recordings; and (2) in each bout, we extracted nonoverlapping epochs (0–1.5 s duration). For walking bouts (10-m sequences, lasting ~10 s), two epochs were extracted, one after the first gait cycle and one before the last two gait cycles. For standing bouts (~5 s each), one epoch was extracted immediately before the onset of walking, and a second epoch was extracted after walking, prior to turning. For sitting, the same number of epochs as for standing and walking were extracted from a continuous 60-s period, while maximizing temporal separation. To further avoid data leakage, (3) we strictly separated training and test sets by eliminating any temporal overlap of feature sets. Each experimental recording (each lasting ~10 min, including >20 walking bouts interleaved with standing and sitting phases) was split into two halves, one for training and one for testing.

Decoder performance was first assessed using cross-validation, and subsequently validated in a pseudo-online manner by replaying the data in chronological order. Training and test sets were strictly separated. For classifiers of L-DOPA or STN DBS condition, performance metrics and illustrative traces were derived directly from raw outputs. For decoders of locomotor activities, probability traces were smoothed to reduce abrupt outliers and better approximate real-time operation. Smoothing was implemented in MATLAB using the movmean function in a strictly causal manner (20 samples, ~500 ms delay in our pseudo-online replay), without access to future data, thereby reflecting performance achievable in real-world applications.

#### Balancing of classes

Class imbalance was mitigated through automatic per-tree weighting, ensuring fair representation of all classes regardless of their original sample proportions. Specifically, the Random Forest classifier (implemented in scikit-learn: RandomForestClassifier, sklearn.ensemble) was configured with class_weight = ‘balanced_subsample’, which adjusts class weights during training to compensate for unequal sample sizes. For each decision tree, class weights are recalculated from the bootstrap sample rather than from the overall dataset, thereby penalizing errors on minority classes more strongly. This dynamic weighting prevents the majority class from dominating predictions and improves recall and overall performance for minority classes.

Reporting of cross-validation performance (Fig. 3, Extended Data Figs. 3 and 4, and Supplementary Fig. 3) includes sample-based confusion matrices across tasks and wF1-score values. Quantification of pseudo-online decoding performance included: (1) median ± s.e.m. probability traces for each class (interpolated in time between the beginning and ending of each state); (2) median probability values of each class across the entire duration of each locomotor state; and (3) wF1-score values to account for class imbalance across locomotor states.

### Classification algorithms of L-DOPA condition or DBS amplitude

Classifiers distinguishing ON versus OFF L-DOPA states, and high versus low DBS amplitudes were derived from the modular decoding framework, with hyperparameters optimized to capture therapy-induced modulations in STN LFPs while minimizing sensitivity to locomotor-related activity. The genetic algorithm identified hyperparameter values that maximized discrimination between L-DOPA and DBS effects, which evolve over distinct timescales (minutes versus near-instantaneous). The most influential parameter was the temporal window length used to compute LFP power: optimal values exceeded 20 s for L-DOPA classification and converged to ~3.8 s for DBS amplitude classification.

### Activity-dependent DBS feasibility study (design)

The feasibility study comprised three full-day experimental sessions (Fig. 5b).

#### Session 1 (eligibility)

Participants were evaluated for cardinal motor symptoms and locomotor deficits using clinical gait assessments, MDS-UPDRS III scores, neurologist evaluations and patient interviews. Clinical tests included the Timed-Up-and-Go (TUG), 10-m walk, 360° turning test, and a freezing-of-gait circuit. Electrode impedance and STN LFP signal quality (artifacts, movement- and therapy-related modulations) were assessed under low and high L-DOPA levels, along with tolerance to DBS amplitude changes.

Participants meeting inclusion criteria proceeded to sessions 2 and 3 (‘Study design’ section). We applied our modular decoding framework to extract individualized neural signatures of locomotor states across varying stimulation intensities and dopaminergic states from the acquired STN LFPs. In parallel, we identified the stimulation amplitudes that optimally alleviated each participant’s most bothersome locomotor deficits under each therapeutic condition.

#### Sessions 2 and 3 (therapy setup and assessment of efficacy)

Conducted on consecutive days, these sessions involved the setup, optimization and testing of activity-dependent DBS based on personalized neural biomarkers. Optimization was performed during repeated back-and-forth walking across medication cycles to refine closed-loop robustness. Acute and sustained effects of activity-dependent DBS were evaluated relative to standard-of-care stimulation (continuous DBS for P1–P3; beta-based aDBS for P4) during in-laboratory and out-of-laboratory gait assessments. Tasks were tailored to each participant’s predominant deficits: repeated walking bouts for gait abnormalities (P1, rigidity; P3, dystonia; P4, weakness) and freezing-of-gait circuits for participants with FOG.

### Investigational DBS platform

The Medtronic aDBS system comprised a CE-marked PC neurostimulator (model B35200) with Sensight DBS leads (model B33015), connected to a Clinician Mobile Platform (model CT900) running an investigational programmer application (A610) enabling research-only sensing and control.

The platform provided continuous real-time monitoring of STN LFP power in a user-defined frequency band (5 Hz bandwidth around a selected frequency of interest in 7.8–100 Hz). Band power was computed using a fast Fourier transform (256-sample window), quantified as the area under the curve in the selected band, and averaged across overlapping windows (80% overlap)^77,78^.

Band-power modulations were used as a control signal to trigger corrections in DBS parameters (upregulation or downregulation of stimulation amplitude) based on one or two thresholds. Three adaptive modes were available^77^:

1. Dual-threshold mode: amplitude increased above the upper threshold, decreased below the lower threshold, and remained unchanged in range,
2. Single-threshold mode: stimulation amplitude is increased when LFP power exceeds the threshold and returned to the lower set amplitude when it goes below the threshold. This mode was not used in the this study, because it requires calibration at 0 mA stimulation, posing challenges during motor tasks for most of the participants,
3. Single-threshold inverse mode: stimulation amplitude is decreased when LFP power exceeds the threshold, and returned to the higher amplitude when it goes below the threshold.

This investigational platform allowed access to features not available in the CE-marked clinical version: (1) use of LFP gamma band power (30–100 Hz) for control of stimulation, in addition to alpha (7–10 Hz) and beta (10–30 Hz) bands; and (2) selection of the single-threshold inverse aDBS mode.

### Identification of participants’ most bothersome gait deficits

Each participant’s most disabling gait impairments were identified during session 1 based on clinical reports, subjective participant interviews, in-laboratory clinical assessments of gait performance by a movement disorders specialist, and quantitative movement analyses performed under both low and high dopaminergic states (Supplementary Table 3). Similar to experiments to characterize the physiological principles of activity-dependent encoding (‘Part 1’), we assessed gait deficits using synchronized whole-body kinematics and bilateral EMG recordings across locomotor tasks. Gait quality was further quantified using a camera-based motion tracking system (OpenCV^79^ and RTMPose^80^) that extracted metrics of posture, balance and inter-limb coordination (Extended Data Fig. 8).

### Identification of optimal DBS amplitudes to address gait deficits

We first verified the range of tolerated stimulation amplitudes for each hemisphere. Tolerability to dynamic changes in amplitude in that range was further assessed using the cycling stimulation mode, in which the implantable neurostimulator was configured to toggle stimulation ON and OFF at two predefined rates (1 s and 50 ms). Participants then performed a series of motor tasks customized to expose their gait deficits, for example walking back-and-forth and ambulation in a FOG circuit, under the following stimulation conditions: (1) the standard-of-care amplitude; (2) the maximal amplitude and minimal amplitudes in the therapeutic window; and (3) 0 mA (when tolerated). Based on motor performance across these conditions, a movement disorders specialist identified the stimulation amplitude that most effectively alleviated each participant’s predominant gait impairment.

In participant P4, we additionally verified that the stimulation amplitudes optimal for cardinal motor symptoms under activity-dependent DBS matched those reached by beta-based aDBS, in both low and high L-DOPA conditions (Extended Data Fig. 9).

### Identification of personalized neural signatures

LFP, EMG and kinematic data were analyzed offline to: (1) assess neural signal quality, including the presence of artifacts such as ECG contamination or movement-induced noise that could interfere with aDBS implementation, and verify acceptable electrode impedance; and (2) evaluate the consistency of LFP modulations associated with locomotor activity across two stimulation amplitudes optimized for different motor symptoms (cardinal and gait-related). No artifacts preventing activity-dependent DBS were detected in the enrolled participants.

To identify the personalized neural signatures, we applied our decoding framework to LFP recordings acquired during motor tasks performed at the two stimulation amplitudes. The training dataset for the decoding algorithm consisted of 30-s periods of rest (sitting) and walking back-and forth. For participants unable to perform sustained walking due to fatigue, continuous leg movements were used as a simplified task. Periods of movement and rest were segmented using EMG and kinematic signals. The tasks were repeated in both high and low dopaminergic states. For participant P1, the low dopaminergic condition was not analyzed due to motor impairment preventing completion of the necessary clinical evaluations.

Spectral features were extracted in the 7–70 Hz range, as allowed by DBS platform constraints for real-time control, which limit the upper bound of the monitored frequency to 235 Hz minus the stimulation frequency (165 Hz for P1) to mitigate stimulation artifacts. The lower bound was increased to 15 Hz in cases in which low-frequency components raised concerns about walking-related artifacts. Decoding analyses were conducted separately for each dopaminergic state using the therapy-specific decoders of our modular decoding framework. For each condition, features were extracted unilaterally, and decoders were trained independently for the left and right hemispheres. The feature importance and the single hemisphere decoding performances informed by the decoding framework allowed us to identify the most informative spectral features per hemisphere to discriminate between locomotor and resting states across different dopaminergic states and stimulation amplitudes.

We finally confirmed visually that movement-related modulations in the identified optimal frequencies corresponded to a synchronization or a desynchronization relative to the resting state (Extended Data Table 1).

### Configuration of activity-dependent adaptive DBS therapy

Hemisphere- and therapy-specific neural features were used to configure activity-dependent DBS for each participant. Supplementary Fig. 4 shows the workflow followed to configure the adaptive therapy.

The optimal frequency of interest for each hemisphere was selected from among the spectral features suggested by the decoders. When multiple candidates were identified, features were ranked by contribution, evaluated online and selected based on real-time reliability. When similar features were observed across dopaminergic conditions, a common frequency of interest was used to enhance stability. In cases of asymmetric decoding performance, a unilateral control signal was used to drive bilateral stimulation.

In three participants (P2, P3 and P4), we found cross-session variations in the stability of the most informative features due to day-to-day variations. The hemisphere and frequency bands used for DBS adaptations were tuned based on the previous ranking to maximize control robustness across these sessions.

The choice of aDBS mode was dictated by both the need to either upregulate or downregulate stimulation amplitude during locomotion to deliver the amplitude that best alleviated gait impairments, and the direction of gait-related LFP modulations (synchronization or desynchronization). Figure 5d and Extended Data Table 1 summarize the control frequencies selected, and whether unilateral or bilateral hemispheres were monitored for DBS control.

Thresholds were calibrated using the tablet interface. To enable robust discrimination of locomotor activities using a threshold-based approach and minimize instabilities during amplitude transitions, LFP recordings for each activity were acquired at distinct stimulation amplitudes. Participants performed 30 s of continuous walking at their standard-of-care DBS amplitude optimized for their cardinal motor symptoms, and a 30-s rest period at the amplitude that best alleviated their gait deficits. In participants unable to perform repeated walking trials, walking was substituted with continuous seated leg movements. This pairing of task and stimulation amplitude was designed to enhance contrast in LFP power between states, thereby improving the reliability of threshold calibration. During these tasks, the DBS platform computed LFP band power in real time and automatically computed threshold values. These values were then manually refined based on analyses from session 1 and visual inspection of the consistency of LFP modulations relative to the threshold during online testing.

Stimulation contact configuration, frequency and pulse width remained constant across all experimental sessions and conditions. Extended Data Table 1 provides a detailed overview of the final aDBS parameters used to target each participant’s gait impairment.

#### Classifier of L-DOPA

The L-DOPA classifier in our decoding framework was implemented pseudo-online to detect L-DOPA states and identify the appropriate frequency bands and triggering thresholds for online use during each phase of the activity-dependent DBS experimental session.

To improve the stability of the pseudo-online L-DOPA classifier across varying DBS amplitudes during full-day recordings, we used longer temporal windows for training. This approach enhanced the robustness of detecting slow therapy-induced fluctuations. The window length was set to 60 s, which is well above the 20 s minimum identified during in-laboratory optimization using the genetic algorithm.

### In-laboratory assessment of acute effects

To evaluate the acute effects of activity-dependent DBS, we selected clinical tests and motor tasks tailored to each participant’s most disabling gait impairment. Tasks were performed under both activity-dependent DBS and standard-of-care DBS, with conditions randomly administered across repetitions:

1. Participant P1: performance was assessed using the clinically validated TUG test (including a 5-m walking bout and turning around a cone), to capture reduced leg agility, impaired right-leg flexion and decreased gait fluidity in the high-L-DOPA condition (Fig. 6c). The test was repeated three times per condition (six times overall). P1 could not be assessed in the low-L-DOPA condition due to severe fatigue and motor impairments that prevented completion of any clinical evaluation,
2. Participant P2: the main deficit was FOG in the low-L-DOPA condition, which occurred predominantly during turning, passing through doorways and obstacle avoidance. We assessed this using the 360° turn test (8 repetitions in each condition) and a customized FOG circuit comprising 3 freezing zones (Extended Data Fig. 8e). Each circuit was repeated twice per condition. P2 did not require DBS adaptations under high L-DOPA, making adaptive stimulation in this condition neither clinically indicated nor ethically justified,
3. Participant P3 reported both FOG and foot dystonia. Performance was evaluated using 19 repetitions of an 8-shaped FOG circuit^81^ (at least six per stimulation condition), together with three recording sessions distributed across the whole day, each comprising six 10-m back-and-forth walking bouts interleaved across stimulation conditions. Among these sessions, only one was conducted at peak medication, during which locomotor deficits were observed, and gait improvement was required,
4. Participant P4 primarily reported sensations of heavy legs and increased effort during prolonged walking, which limited endurance. Accordingly, we assessed gait performance during repeated series (*n* = 4 repetitions) of three continuous back-and-forth 8-m walking trials with turns around a cone, interleaved with 30-s sitting phases (Extended Data Fig. 9c,d). Over the course of session 2, the participant performed 36 back-and-forth bouts for each therapeutic condition at three time-points of L-DOPA cycles. Analyses were performed separately for the first and last two series across time-points to better capture fatigue-related effects and separately for each time-point to assess therapy-related changes across L-DOPA cycles.

LFP and biomechanical signals were recorded during all tasks using our multimodal monitoring platform. FOG episodes were identified from video recordings by a movement disorders specialist. FOG episode durations were quantified using EMG analysis, and turning sequences were identified by gyroscope signals, as described above. Gait quality was assessed both by a movement disorders neurologist and through quantitative kinematic and EMG parameters.

### Out-of-laboratory assessments

To evaluate the sustained efficacy and robustness of activity-dependent DBS in real-world conditions, participants performed out-of-laboratory tasks during session 3 under both activity-dependent DBS and standard-of-care DBS. Stimulation conditions were randomized and blinded to participants until session completion.

Assessments were designed to reflect naturalistic walking conditions. Participant P1 walked around the neighborhood with interleaved rest periods. Participant P2 walked along a path in his hotel environment to elicit FOG episodes. Participant P3 walked outdoors, navigating pedestrian traffic and environmental obstacles, and transitioned in and out of buildings (for example, entering a cafeteria). Participant P4 walked in hospital premises and around the neighborhood, and completed continuous ambulation around a 300-m track (six laps total, interleaving standard-of-care DBS and activity-dependent DBS every two laps) to better monitor fatigue-related effects (Extended Data Fig. 9g). Total durations of out-of-lab assessments were: P1, 32 min; P2, 41 min; P3, 49 min; and P4, 68 min. Participants were continuously accompanied by a member of the research team to ensure safety and allow for real-time monitoring.

LFP and kinematic data were acquired using NUSHU sensorized shoes (Magnes) and synchronized with STN LFP recordings. For participant P2, a portable EMG system (Delsys; operating at 1.3 kHz) was additionally used to record activity from two leg muscles (left vastus medialis and tibialis anterior) to quantify FOG duration, and two IMU sensors (sampling frequency: 370 Hz) were placed on each foot to identify turning sequence. Analyses of kinematic and neural data were conducted offline following the experiments to compare gait quality between activity-dependent DBS and standard-of-care DBS.

The reliability of activity-dependent DBS throughout real-world tasks was quantified using wF1-scores and by evaluating the proportion of time spent at each stimulation amplitude (cardinal and gait-related) for each movement state, calculated as the number of correctly predicted labels divided by the total number of labels for that state.

Participants’ subjective experience was assessed using custom-designed questionnaires rating the perceived impact of activity-dependent DBS on various Parkinsonian symptoms (gait, FOG, balance, dyskinesia, bradykinesia, rigidity, tremor and cognition) using a scale from −10 (much worse) to +10 (much better) relative to standard-of-care stimulation.

### Setup and iterative optimization of ‘beta-based’ adaptive DBS

Before enrollment in the activity-dependent DBS study, participant P4 had been treated with beta-based aDBS for three months (Extended Data Fig. 7). During this period, six in-clinic sessions were conducted, including an initial visit to identify participant-specific frequency bands and initiate BrainSense Timeline recordings, followed by five sessions for setup and iterative parameters optimization. Across these sessions, triggering thresholds and DBS amplitude limits were refined based on aDBS behavior and participant feedback.

#### aDBS setup

Procedures followed recently established protocols^63,64,82^, using a dual-threshold strategy calibrated with up to 15 days of longitudinal BrainSense Timeline sensing. The maximal beta peak was identified from BrainSense Survey recordings acquired in both OFF- and ON-medication conditions and retained only if its amplitude exceeded a predefined threshold to ensure robustness (1.2 μVp). In P4, low-beta activity (16.6 Hz) was selected bilaterally to drive pharmacokinetics-related adaptations.

#### Iterative adaptation of aDBS parameters

Every two weeks, or upon individual request, the participant attended in-clinic optimization sessions to adjust triggering thresholds and stimulation limits to prevent stimulation-related adverse effects and avoid saturation at minimum or maximum amplitudes. This process was supported by custom-made code that computed average LFP band power and DBS adaptations within and across days, and was guided by participant-reported quality-of-life measures. After 10 weeks, the participant reported overall satisfaction with beta-based aDBS, including improved sleep quality and mobility. Adaptive therapy was used >95% of the time, and improved MDS-UPDRS III scores. However, persistent gait-related difficulties remained and prompted discontinuation of adaptive therapy with manual increases in stimulation amplitude during prolonged walking.

### Data analysis

In-laboratory gait quality was quantified using EMG and kinematic parameters. EMG envelopes were extracted per gait cycle, time-normalized and compared across therapeutic conditions (activity-dependent DBS versus standard-of-care DBS) using unpaired two-tailed *t*-tests with Bonferroni correction (*n* = number of channels) implemented in Statistical Parametric Mapping (SPM1D; https://spm1d.org).

Kinematic parameters were extracted using camera-based motion tracking in Python^7,8^ and analyzed using principal component analysis. Whole-body kinematics (joint angles and velocities, trunk motion, gait-phase timing) were parameterized per gait cycle into 144 features, *z*-scored and reduced using minimum redundancy–maximum relevance (feature_engine Python library) with a 1% rejection threshold to limit instability due to high feature-to-sample ratios. Resulting subject-specific feature sets (P1, 84; P2, 80; P3, 90; P4, 107) were projected into PC space.

Six healthy controls were included as a normative reference. The effect of activity-dependent DBS was quantified using the Bhattacharyya distance between condition centroids and controls, compared using Wilcoxon signed-rank tests. To ensure stability, the number of PCs retained explained ≥50% of the variance (P1 and P4, six PCs; P2, four PCs; P3, five PCs). PCs were ranked by separability between the two therapeutic conditions using Fisher scores, and the two-dimensional subspace maximizing conditions separation was used for visualization (Extended Data Fig. 8c). Feature contributions were grouped into biomechanical domains (joint-specific, trunk, temporal and proportional gait-phase components, gait direction) to identify determinants of gait modulation.

Out-of-laboratory gait was quantified using nine parameters per gait cycle (such as stride velocity, stride length, swing–stance ratio, supination angle, strike angle, stride duration, lateral swing, heel clearance, double-support time) extracted from the NUSHU platform (Magnes) and compared using unpaired two-tailed *t*-tests.

### Statistical methods

All analyses were performed offline in MATLAB R2024a,b (MathWorks). Data are reported as mean or median ± s.d. or s.e.m., as indicated. Normality was assessed using the Kolmogorov–Smirnov test (95% confidence interval). Parametric or nonparametric tests were applied accordingly: two-tailed *t*-tests or Wilcoxon signed-rank tests for two-condition comparisons, and one-way ANOVA, repeated-measures ANOVA or Kruskal–Wallis tests for multigroup comparisons, followed by Bonferroni-corrected post-hoc tests. Statistical significance was set at α = 0.05. Boxplots display median (center line), interquartile range (box, 25th–75th percentiles), and most extreme values within 1.5 times the interquartile range (whiskers); values beyond this range are shown as outliers. Exact tests and *P* values are reported in figure captions.

### Reporting summary

Further information on research design is available in the Nature Portfolio Reporting Summary linked to this article.

## Online content

Any methods, additional references, Nature Portfolio reporting summaries, source data, extended data, supplementary information, acknowledgements, peer review information; details of author contributions and competing interests; and statements of data and code availability are available at 10.1038/s41591-026-04432-4.

## Supplementary information

Supplementary Information Supplementary Figs. 1–5 and Tables 1–7.

Reporting Summary

Supplementary Video 1 Physiological principles of activity-dependent STN dynamics across therapeutic conditions.

Supplementary Video 2 Modular decoding framework to predict locomotor activities across L-DOPA fluctuations.

Supplementary Video 3 Activity-dependent DBS therapies alleviate locomotor deficits and cardinal motor symptoms.

## Figures and Tables

**Extended Data Table 1:**
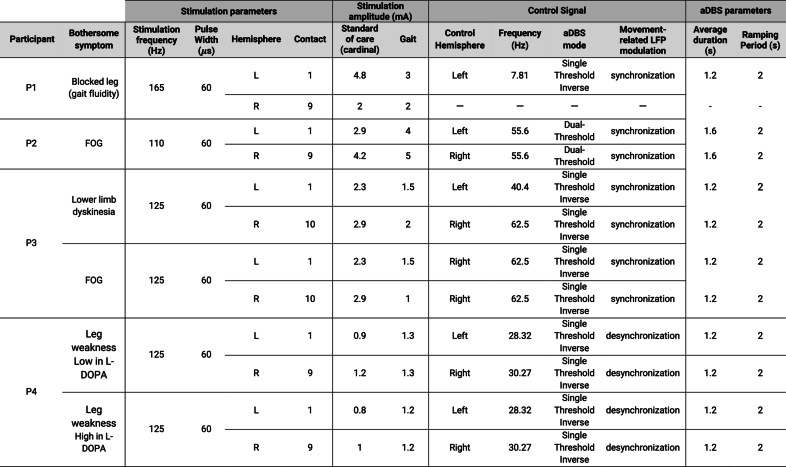
STN DBS parameters used for activity-dependent adaptive DBS

All participants were stimulated using monopolar configurations. The monitored frequency corresponds to the center of the 5-Hz band selected during participant-specific biomarker identification. In participant P1, activity-dependent adaptive DBS was delivered unilaterally in the left hemisphere to target the more affected right hemibody. In participant P3, bilateral stimulation was controlled via sensing from a single hemisphere, with sensing disabled contralaterally to allow this configuration. Participant P4 exhibited the most bothersome gait deficit in both dopaminergic conditions; stimulation amplitudes were optimized separately under each condition.

## Data Availability

All data associated with this study are present in the paper, as well as in Extended Data Figs. 1–9, Extended Data Table 1 or Supplementary Information. Anonymized datasets and scripts that support the findings of this study are available via Zenodo at 10.5281/zenodo.19371521 (ref. ^83^).

## Code availability

The custom code used in this study, including the implementation of the decoding framework, is available via Zenodo at 10.5281/zenodo.19371521 (ref. ^83^).

## References

[1] Mirelman, A. et al. Gait impairments in Parkinson’s disease. *Lancet Neurol.* **18**, 697–708 (2019). 30975519 10.1016/S1474-4422(19)30044-4

[2] Nonnekes, J. et al. Neurological disorders of gait, balance and posture: a sign-based approach. *Nat. Rev. Neurol.* **14**, 183–189 (2018). 29377011 10.1038/nrneurol.2017.178

[3] Fasano, A., Aquino, C. C., Krauss, J. K., Honey, C. R. & Bloem, B. R. Axial disability and deep brain stimulation in patients with Parkinson disease. *Nat. Rev. Neurol.* **11**, 98–110 (2015). 25582445 10.1038/nrneurol.2014.252

[4] Collomb-Clerc, A. & Welter, M.-L. Effects of deep brain stimulation on balance and gait in patients with Parkinson’s disease: a systematic neurophysiological review. *Neurophysiol. Clin.* **45**, 371–388 (2015). 26319759 10.1016/j.neucli.2015.07.001

[5] Bloem, B. R., Hausdorff, J. M., Visser, J. E. & Giladi, N. Falls and freezing of gait in Parkinson’s disease: a review of two interconnected, episodic phenomena. *Mov. Disord.* **19**, 871–884 (2004). 15300651 10.1002/mds.20115

[6] Lau, B. et al. Axial symptoms predict mortality in patients with Parkinson disease and subthalamic stimulation. *Neurology* **92**, e2559–e2570 (2019). 31043471 10.1212/WNL.0000000000007562 PMC6556086

[7] Maetzler, W., Nieuwhof, F., Hasmann, S. E. & Bloem, B. R. Emerging therapies for gait disability and balance impairment: promises and pitfalls. *Mov. Disord.* **28**, 1576–1586 (2013). 24132846 10.1002/mds.25682

[8] Picillo, M., Lozano, A. M., Kou, N., Munhoz, R. P. & Fasano, A. Programming deep brain stimulation for Parkinson’s disease: the Toronto Western Hospital algorithms. *Brain Stimul.* **9**, 425–437 (2016). 26968806 10.1016/j.brs.2016.02.004

[9] Koeglsperger, T., Palleis, C., Hell, F., Mehrkens, J. H. & Bötzel, K. Deep brain stimulation programming for movement disorders: current concepts and evidence-based strategies. *Front. Neurol.* **10**, 410 (2019). 31231293 10.3389/fneur.2019.00410 PMC6558426

[10] Daalen, J. M. J. et al. Gait and balance worsening after bilateral deep brain stimulation of the subthalamic nucleus (STN-DBS) for Parkinson’s disease: a systematic review. *BMJ Neurol. Open* **7**, e000898 (2025). 10.1136/bmjno-2024-000898 PMC11907047 40092840

[11] Cherif, S. et al. Directional subthalamic deep brain stimulation better improves gait and balance disorders in Parkinson’s disease patients: a randomized controlled study. *Ann. Neurol.* **97**, 149–162 (2024). 39475137 10.1002/ana.27099 PMC11683173

[12] Conway, Z. J., Silburn, P. A., Perera, T., O’Maley, K. & Cole, M. H. Low-frequency STN-DBS provides acute gait improvements in Parkinson’s disease: a double-blinded randomised cross-over feasibility trial. *J. Neuroeng. Rehabil.* **18**, 125 (2021). 34376190 10.1186/s12984-021-00921-4 PMC8353795

[13] Yogev-Seligmann, G., Hausdorff, J. M. & Giladi, N. The role of executive function and attention in gait. *Mov. Disord.* **23**, 329–342 (2008). 18058946 10.1002/mds.21720 PMC2535903

[14] Tosserams, A. et al. Management of freezing of gait—mechanism-based practical recommendations. *Nat. Rev. Neurol.* **21**, 327–344 (2025). 40169855 10.1038/s41582-025-01079-6

[15] Fekri Azgomi, H. et al. Modeling and optimizing deep brain stimulation to enhance gait in Parkinson’s disease: personalized treatment with neurophysiological insights. *npj Parkinsons Dis.* **11**, 173 (2025). 40533464 10.1038/s41531-025-00990-5 PMC12177069

[16] Obeso, J. A. et al. Functional organization of the basal ganglia: therapeutic implications for Parkinson’s disease. *Mov. Disord.* **23**, S548–S559 (2008). 18781672 10.1002/mds.22062

[17] Dudman, J. T. & Krakauer, J. W. The basal ganglia: from motor commands to the control of vigor. *Curr. Opin. Neurobiol.* **37**, 158–166 (2016). 27012960 10.1016/j.conb.2016.02.005

[18] Hammond, C., Bergman, H. & Brown, P. Pathological synchronization in Parkinson’s disease: networks, models and treatments. *Trends Neurosci.* **30**, 357–364 (2007). 17532060 10.1016/j.tins.2007.05.004

[19] Hell, F., Plate, A., Mehrkens, J. H. & Bötzel, K. Subthalamic oscillatory activity and connectivity during gait in Parkinson’s disease. *Neuroimage Clin.* **19**, 396–405 (2018). 30035024 10.1016/j.nicl.2018.05.001 PMC6051498

[20] Louie, K. H. et al. Cortico-subthalamic field potentials support classification of the natural gait cycle in Parkinson’s disease and reveal individualized spectral signatures. *eNeuro* **9**, ENEURO.0325-22.2022 (2022). 36270803 10.1523/ENEURO.0325-22.2022 PMC9663205

[21] Thenaisie, Y. et al. Principles of gait encoding in the subthalamic nucleus of people with Parkinson’s disease. *Sci. Transl. Med.* **14**, eabo1800 (2022). 36070366 10.1126/scitranslmed.abo1800

[22] Choi, J. W., Cui, C., Wilkins, K. B. & Bronte-Stewart, H. M. N2GNet tracks gait performance from subthalamic neural signals in Parkinson’s disease. *npj Digit. Med.* **8**, 7 (2025). 39755754 10.1038/s41746-024-01364-6 PMC11700158

[23] Georgiades, M. J. et al. Hitting the brakes: pathological subthalamic nucleus activity in Parkinson’s disease gait freezing. *Brain* **142**, 3906–3916 (2019). 31665229 10.1093/brain/awz325

[24] Fischer, P. et al. Alternating modulation of subthalamic nucleus beta oscillations during stepping. *J. Neurosci.* **38**, 5111–5121 (2018). 29760182 10.1523/JNEUROSCI.3596-17.2018 PMC5977446

[25] Fischer, P. et al. Entraining stepping movements of Parkinson’s patients to alternating subthalamic nucleus deep brain stimulation. *J. Neurosci.* **40**, 8964–8972 (2020). 33087473 10.1523/JNEUROSCI.1767-20.2020 PMC7659462

[26] Thenaisie, Y. et al. Towards adaptive deep brain stimulation: clinical and technical notes on a novel commercial device for chronic brain sensing. *J. Neural Eng.* **18**, 042002 (2021). 10.1088/1741-2552/ac1d5b 34388744

[27] Krauss, J. K. et al. Technology of deep brain stimulation: current status and future directions. *Nat. Rev. Neurol.* **17**, 75–87 (2021). 33244188 10.1038/s41582-020-00426-z PMC7116699

[28] Cagnan, H., Denison, T., McIntyre, C. & Brown, P. Emerging technologies for improved deep brain stimulation. *Nat. Biotechnol.* **37**, 1024–1033 (2019). 31477926 10.1038/s41587-019-0244-6 PMC6877347

[29] Neumann, W. J. et al. Subthalamic synchronized oscillatory activity correlates with motor impairment in patients with Parkinson’s disease. *Mov. Dis.* **31**, 1748–1751 (2016). 10.1002/mds.26759 PMC5120686 27548068

[30] Tinkhauser, G. et al. The modulatory effect of adaptive deep brain stimulation on beta bursts in Parkinson’s disease. *Brain* **140**, 1053–1067 (2017). 28334851 10.1093/brain/awx010 PMC5382944

[31] Mathiopoulou, V. et al. Gamma entrainment induced by deep brain stimulation as a biomarker for motor improvement with neuromodulation. *Nat. Commun.* **16**, 2956 (2025). 40140380 10.1038/s41467-025-58132-7 PMC11947250

[32] Little, S. et al. Adaptive deep brain stimulation in advanced Parkinson disease. *Ann. Neurol.* **74**, 449–457 (2013). 23852650 10.1002/ana.23951 PMC3886292

[33] Priori, A., Foffani, G., Rossi, L. & Marceglia, S. Adaptive deep brain stimulation (aDBS) controlled by local field potential oscillations. *Exp. Neurol.* **245**, 77–86 (2013). 23022916 10.1016/j.expneurol.2012.09.013

[34] Oehrn, C. R. et al. Chronic adaptive deep brain stimulation versus conventional stimulation in Parkinson’s disease: a blinded randomized feasibility trial. *Nat. Med.* **30**, 3345–3356 (2024). 39160351 10.1038/s41591-024-03196-z PMC11826929

[35] Dixon, T. C. et al. Movement-responsive deep brain stimulation for Parkinson’s disease using a remotely optimized neural decoder. *Nat. Biomed. Eng.* **10**, 110–124 (2026). 40579487 10.1038/s41551-025-01438-0

[36] Bronte-Stewart, H. M. et al. Long-term personalized adaptive deep brain stimulation in Parkinson disease: a nonrandomized clinical trial. *JAMA Neurol.* **82**, 1171–1180 (2025). 40982287 10.1001/jamaneurol.2025.2781 PMC12455485

[37] Isaias, I. U. et al. Chronic adaptive versus conventional deep brain stimulation in Parkinson’s disease: a blinded randomized pilot trial. Preprint at *medRxiv*10.1101/2025.02.20.25322374 (2025).

[38] Tan, H. Towards adaptive deep brain stimulation for freezing of gait. *Brain* **145**, 2236–2238 (2022). 35848860 10.1093/brain/awac172 PMC9337803

[39] Klocke, P., Loeffler, M. A., Lewis, S. J., Gharabaghi, A. & Weiss, D. Could adaptive deep brain stimulation treat freezing of gait in Parkinson’s disease? *J. Neurol.* **272**, 267 (2025). 40072634 10.1007/s00415-025-13000-8 PMC11903562

[40] Isaias, I. U. et al. Case report: Improvement of gait with adaptive deep brain stimulation in a patient with Parkinson’s disease. *Front. Bioeng. Biotechnol.* **12**, 1428189 (2024). 39323762 10.3389/fbioe.2024.1428189 PMC11423205

[41] Wilkins, K. B. et al. Beta burst-driven adaptive deep brain stimulation improves gait impairment and freezing of gait in Parkinson’s disease. *Brain Commun.* **7**, fcaf266 (2025). 40677701 10.1093/braincomms/fcaf266 PMC12268161

[42] Donoghue, T. et al. Parameterizing neural power spectra into periodic and aperiodic components. *Nat. Neurosci.* **23**, 1655–1665 (2020). 33230329 10.1038/s41593-020-00744-x PMC8106550

[43] Echeverria-Altuna, I. et al. Transient beta activity and cortico-muscular connectivity during sustained motor behaviour. *Prog. Neurobiol.* **214**, 102281 (2022). 35550908 10.1016/j.pneurobio.2022.102281 PMC9742854

[44] Simpson, T. G. et al. Cortical beta oscillations help synchronise muscles during static posture holding in healthy motor control. *Neuroimage* **298**, 120774 (2024). 39103065 10.1016/j.neuroimage.2024.120774 PMC7617462

[45] Klocke, P. et al. Supraspinal contributions to defective antagonistic inhibition and freezing of gait in Parkinson’s disease. *Brain* **147**, 4056–4071 (2024). 39470410 10.1093/brain/awae223

[46] Lubik, S. et al. Gait analysis in patients with advanced Parkinson disease: different or additive effects on gait induced by levodopa and chronic STN stimulation. *J. Neural Transm.* **113**, 163–173 (2006). 15959852 10.1007/s00702-005-0310-8

[47] Mathiopoulou, V. et al. Modulation of subthalamic beta oscillations by movement, dopamine, and deep brain stimulation in Parkinson’s disease. *npj Parkinsons Dis.* **10**, 77 (2024). 38580641 10.1038/s41531-024-00693-3 PMC10997749

[48] Binns, T. S. et al. Shared pathway-specific network mechanisms of dopamine and deep brain stimulation for the treatment of Parkinson’s disease. *Nat. Commun.* **16**, 3587 (2025). 40234441 10.1038/s41467-025-58825-z PMC12000430

[49] Lee, K. et al. In *Brain–Computer Interface Research: A State-of-the-Art Summary 11* (eds Guger, C. et al.) 83–92 (Springer, 2024).

[50] López, P. S. et al. A wearable neuromonitoring platform to map motor and neural biomarkers of gait dysfunction in people with Parkinson’s disease across daily life. In *2025 47th Annual International Conference of the IEEE Engineering in Medicine and Biology Society**(**EMBC**)* 1–5 (IEEE, 2025). 10.1109/EMBC58623.2025.11253696 41336994

[51] Swinnen, B. E. et al. Pitfalls and practical suggestions for using local field potential recordings in DBS clinical practice and research. *J. Neural Eng.* **22**, 014001 (2025). 10.1088/1741-2552/adaeee 39870045

[52] Tan, H. et al. Decoding gripping force based on local field potentials recorded from subthalamic nucleus in humans. *eLife* **5**, e19089 (2016). 27855780 10.7554/eLife.19089 PMC5148608

[53] Tan, H. et al. Subthalamic nucleus local field potential activity helps encode motor effort rather than force in parkinsonism. *J. Neurosci.* **35**, 5941–5949 (2015). 25878267 10.1523/JNEUROSCI.4609-14.2015 PMC4397595

[54] Köhler, R. M. et al. Dopamine and deep brain stimulation accelerate the neural dynamics of volitional action in Parkinson’s disease. *Brain* **147**, 3358–3369 (2024). 38954651 10.1093/brain/awae219 PMC11449126

[55] Tinkhauser, G. & Moraud, E. M. Controlling clinical states governed by different temporal dynamics with closed-loop deep brain stimulation: a principled framework. *Front. Neurosci.* **15**, 734186 (2021). 34858126 10.3389/fnins.2021.734186 PMC8632004

[56] Dold, M. et al. Dareplane: a modular open-source software platform for BCI research with application in closed-loop deep brain stimulation. *J. Neural Eng.* **22**, 026029 (2025). 10.1088/1741-2552/adbb20 40014925

[57] Merk, T. et al. Invasive neurophysiology and whole brain connectomics for neural decoding in patients with brain implants. *Nat. Biomed. Eng.*10.1038/s41551-025-01467-9 (2025). 10.1038/s41551-025-01467-9 PMC13190260 40993190

[58] Herron, J. et al. Challenges and opportunities of acquiring cortical recordings for chronic adaptive deep brain stimulation. *Nat. Biomed. Eng.* **9**, 606–617 (2025). 39730913 10.1038/s41551-024-01314-3 PMC12413772

[59] Rosin, B. et al. Closed-loop deep brain stimulation is superior in ameliorating parkinsonism. *Neuron* **72**, 370–384 (2011). 22017994 10.1016/j.neuron.2011.08.023

[60] Bronte-Stewart, H. M. et al. Perspective: Evolution of control variables and policies for closed-loop deep brain stimulation for Parkinson’s disease using bidirectional deep-brain–computer interfaces. *Front. Hum. Neurosci.* **14**, 353 (2020). 33061899 10.3389/fnhum.2020.00353 PMC7489234

[61] Bouthour, W. et al. Biomarkers for closed-loop deep brain stimulation in Parkinson disease and beyond. *Nat. Rev. Neurol.* **15**, 343–352 (2019). 30936569 10.1038/s41582-019-0166-4

[62] Feldmann, L. K. et al. Toward therapeutic electrophysiology: beta-band suppression as a biomarker in chronic local field potential recordings. *npj Parkinsons Dis.* **8**, 44 (2022). 35440571 10.1038/s41531-022-00301-2 PMC9018912

[63] Busch, J. L. et al. Chronic adaptive deep brain stimulation for Parkinson’s disease: clinical outcomes and programming strategies. *npj Parkinsons Dis.* **11**, 264 (2025). 40883328 10.1038/s41531-025-01124-7 PMC12397205

[64] Fasano, A. et al. ADAPT-PD Clinical Trial: programming adaptive deep brain stimulation in the clinic. *Neuromodulation* **29**, S167 (2026).

[65] Rajamani, N. et al. Deep brain stimulation of symptom-specific networks in Parkinson’s disease. *Nat. Commun.* **15**, 4662 (2024). 38821913 10.1038/s41467-024-48731-1 PMC11143329

[66] Horn, A. & Neumann, W.-J. From adaptive deep brain stimulation to adaptive circuit targeting. *Nat. Rev. Neurol.* **21**, 556–566 (2025). 40903613 10.1038/s41582-025-01131-5

[67] Lawrence, D. et al. Cortico-basal oscillations index naturalistic movements during deep brain stimulation. *Brain* awaf466 (2025). 10.1093/brain/awaf466 PMC13431667 41399243

[68] Guidetti, M. et al. Clinical perspectives of adaptive deep brain stimulation. *Brain Stimul.* **14**, 1238–1247 (2021). 34371211 10.1016/j.brs.2021.07.063

[69] Milekovic, T. et al. A spinal cord neuroprosthesis for locomotor deficits due to Parkinson’s disease. *Nat. Med.* **29**, 2854–2865 (2023). 37932548 10.1038/s41591-023-02584-1

[70] Guidetti, M. et al. Will adaptive deep brain stimulation for Parkinson’s disease become a real option soon? A Delphi consensus study. *npj Parkinsons Dis.* **11**, 110 (2025). 40325017 10.1038/s41531-025-00974-5 PMC12052990

[71] Horn, A. & Kühn, A. A. Lead-DBS: a toolbox for deep brain stimulation electrode localizations and visualizations. *Neuroimage* **107**, 127–135 (2015). 25498389 10.1016/j.neuroimage.2014.12.002

[72] Avants, B. B. et al. A reproducible evaluation of ANTs similarity metric performance in brain image registration. *Neuroimage* **54**, 2033–2044 (2011). 20851191 10.1016/j.neuroimage.2010.09.025 PMC3065962

[73] Fonov, V. et al. Unbiased average age-appropriate atlases for pediatric studies. *Neuroimage* **54**, 313–327 (2011). 20656036 10.1016/j.neuroimage.2010.07.033 PMC2962759

[74] Husch, A., Petersen, M. V., Gemmar, P., Goncalves, J. & Hertel, F. PaCER—a fully automated method for electrode trajectory and contact reconstruction in deep brain stimulation. *Neuroimage Clin.* **17**, 80–89 (2018). 29062684 10.1016/j.nicl.2017.10.004 PMC5645007

[75] Stam, M. et al. A comparison of methods to suppress electrocardiographic artifacts in local field potential recordings. *Clin. Neurophysiol.* **146**, 147–161 (2023). 36543611 10.1016/j.clinph.2022.11.011

[76] Nocilli, M. et al. GAIT: gait analysis interactive tool a pipeline for automatic detection of gait events across different motor impairments. *Signal Image Video Process.* **18**, 8499–8506 (2024).

[77] Stanslaski, S. et al. Sensing data and methodology from the Adaptive DBS Algorithm for Personalized Therapy in Parkinson’s Disease (ADAPT-PD) clinical trial. *npj Parkinsons Dis.* **10**, 174 (2024). 39289373 10.1038/s41531-024-00772-5 PMC11408616

[78] Jimenez-Shahed, J. Device profile of the percept PC deep brain stimulation system for the treatment of Parkinson’s disease and related disorders. *Expert Rev. Med. Devices* **18**, 319–332 (2021). 33765395 10.1080/17434440.2021.1909471

[79] Bradski, G. The OpenCV library. *Dr. Dobbs J. Softw. Tools* **25**, 120–123 (2000).

[80] Jiang, T. et al. Rtmpose: real-time multi-person pose estimation based on mmpose. Preprint at https://arxiv.org/abs/2303.07399 (2023).

[81] O’Day, J. et al. The turning and barrier course reveals gait parameters for detecting freezing of gait and measuring the efficacy of deep brain stimulation. *PLoS ONE* **15**, e0231984 (2020). 32348346 10.1371/journal.pone.0231984 PMC7190141

[82] Cascino, S. et al. Chronic adaptive deep brain stimulation in Parkinson’s disease: ADAPT-START findings and programming principles. *npj Parkinsons Dis.* **12**, 85 (2026). 41741462 10.1038/s41531-026-01269-z PMC13056985

[83] Scafa, S. et al. Activity-dependent adaptive deep brain stimulation improves gait in Parkinson’s disease. *Zenodo*10.5281/zenodo.19371521 (2026). 10.1038/s41591-026-04432-4 PMC13472876 42297979

