# Supplementary material for: Activity-dependent adaptive deep brain stimulation improves gait in Parkinson’s disease
Source: Nat Med. 2026 Jun 15;32(8):2815–30. doi: 10.1038/s41591-026-04432-4 (PMC13472876; doi:10.1038/s41591-026-04432-4)
Supplement: Supplementary file 1 [file 41591_2026_4432_MOESM1_ESM.pdf]

nature medicine

Supplementary information https://doi.org/10.1038/s41591-026-04432-4

Activity-dependent adaptive deep brain stimulation improves gait in Parkinson's disease

In the format provided by the authors and unedited

Supplementary materials

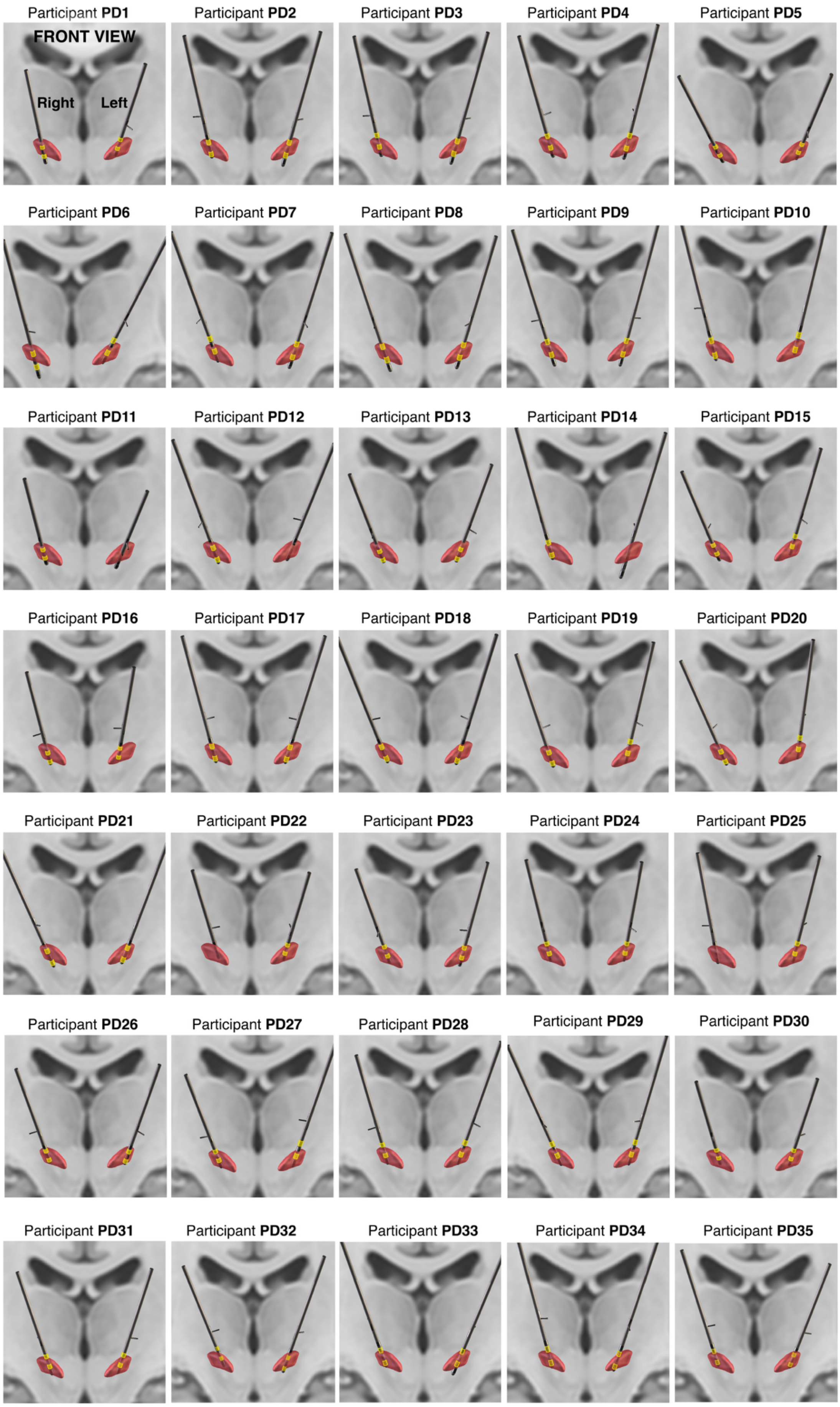

Supplementary Figure 1 | Anatomical reconstructions of deep brain stimulation lead placement across participants. Bipolar contacts used for STN LFP recordings are highlighted in yellow for each hemisphere.

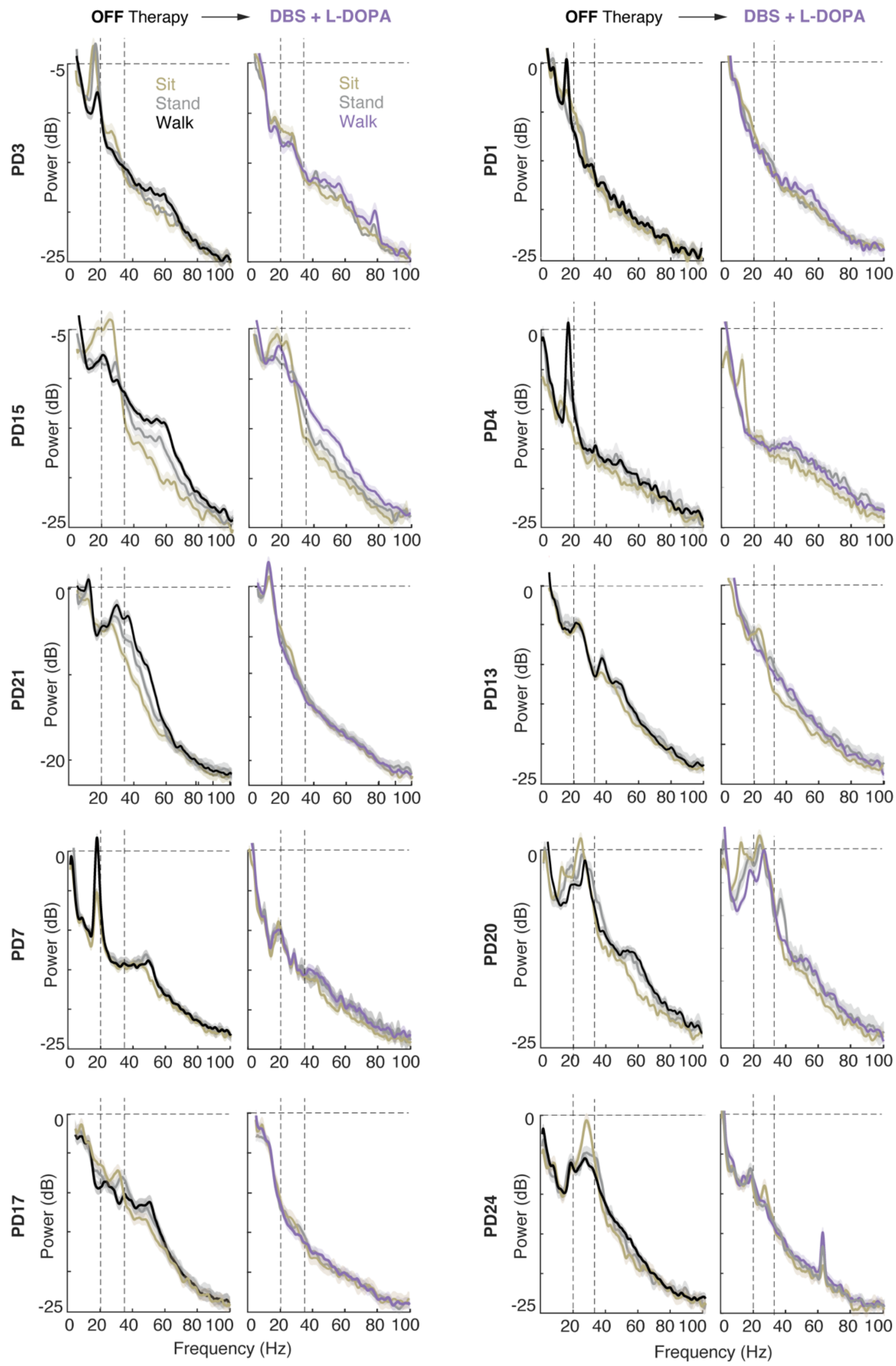

Supplementary Figure 2 | Electrophysiological characteristics of activity-dependent modulations within STN dynamics across participants. Ten illustrative participants are shown in their most parkinsonian state (OFF therapy) and under their standard-of-care combination of STN DBS and L-DOPA. For each participant, power spectral densities (PSD; Welch's method, mean ± 95% confidence interval) are displayed for each activity. Dashed lines indicate the boundaries of the canonical high-beta band (20-35 Hz). Several of these participants are featured in other figures throughout the manuscript to illustrate activity- and therapy-specific modulations. Comparisons under standardized conditions is intended to facilitate interpretation of the individualized adaptations presented throughout the manuscript.

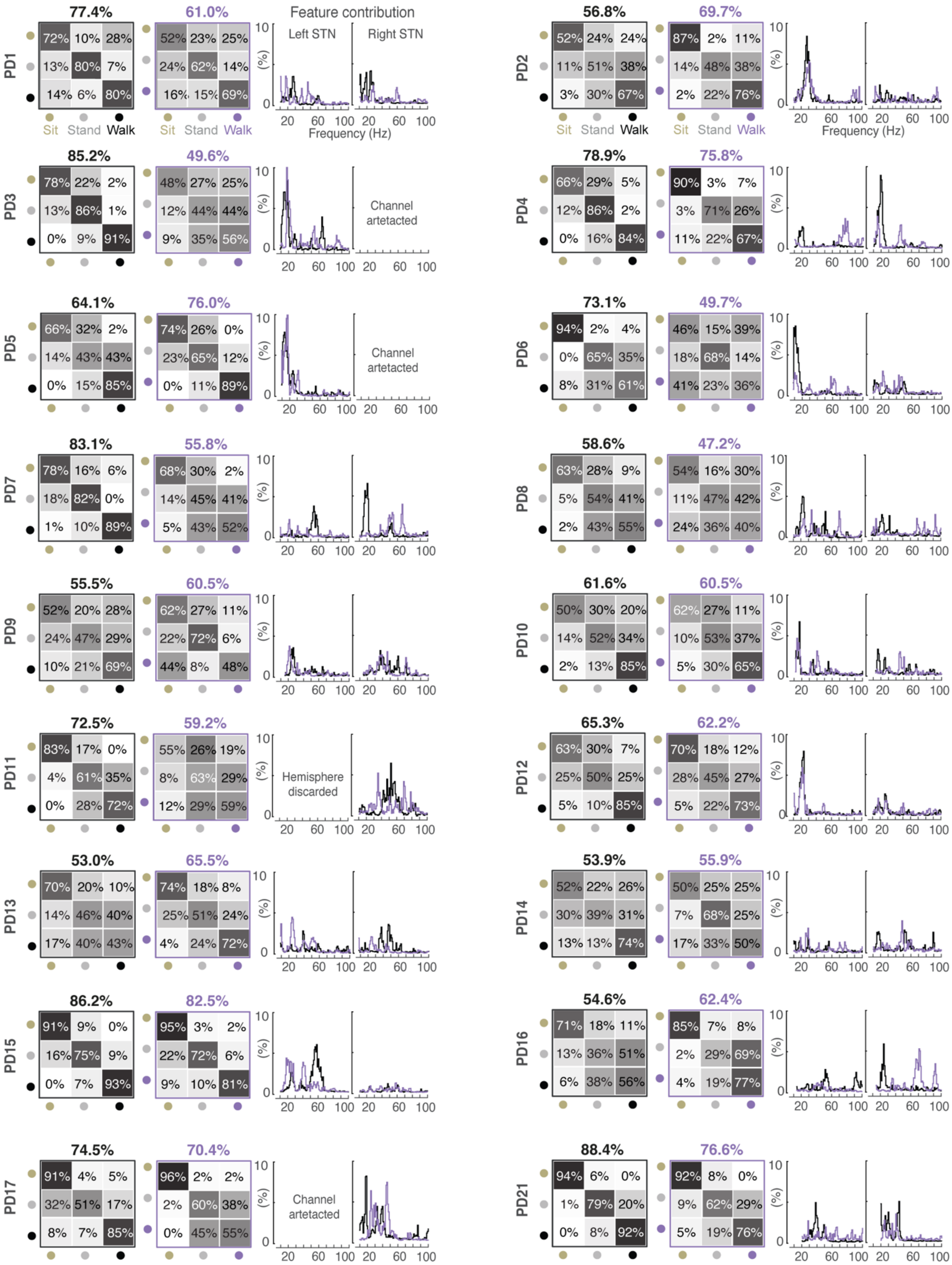

Supplementary Figure 3 | Participant-specific decoding performance of locomotor states under each therapeutic condition.

Confusion matrices showing cross-validation performance and spectral feature contributions for each decoder, shown by participant and therapeutic condition (OFF-therapy: black; Standard-of-care combination of STN-DBS + L-DOPA: purple). Average wF1 score OFF therapy: 69% across the three classes; ON therapy: 63.4%) Channels excluded due to artifacts or suboptimal electrode placement are indicated accordingly.

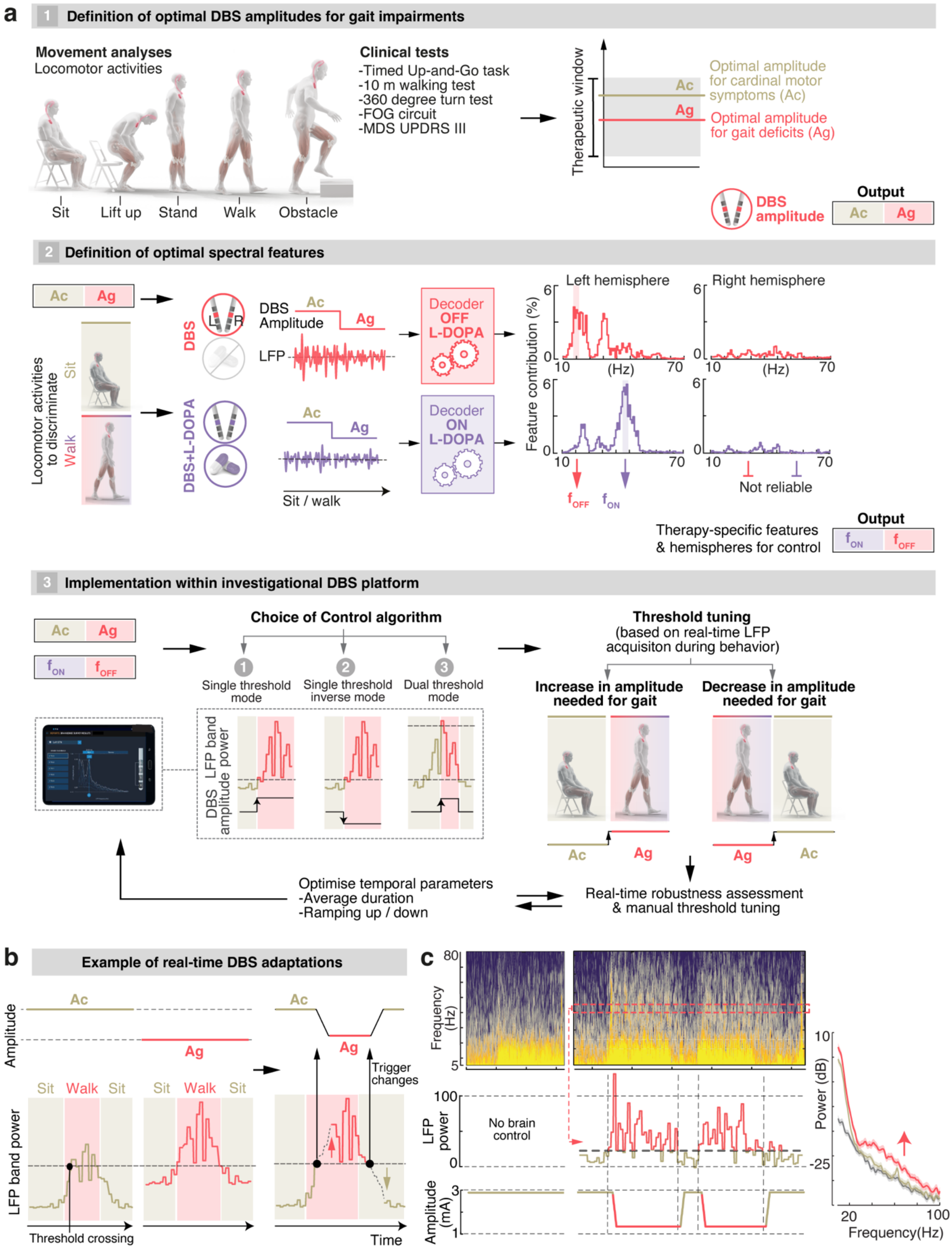

Supplementary Figure 4 | Implementation workflow for activity-dependent adaptive DBS.

(a) The workflow is organized into three steps. First, we identified patient-specific locomotor deficits under the standard-of-care DBS amplitude and determined the optimal DBS amplitude to address these deficits. The impact of the new DBS amplitude on cardinal motor symptoms was also evaluated. Second, we identified the optimal spectral features for discriminating locomotor activities. Thes features were computed for each hemisphere, separately under high and low L-DOPA conditions, but across varying DBS amplitudes to ensure stability during real-time modulation of stimulation amplitude. Third, we implemented the selected parameters into the investigational DBS platform. This involved selecting the control mode that enabled up- or down-regulation of stimulation amplitude based on the chosen neural biomarkers, and tuning the triggering threshold and temporal parameters to maximize robustness.

(b) Scheme illustrating how threshold tuning must capture movement-related modulations in STN LFPs across both therapeutic conditions (under standard-of-care DBS amplitude and at the optimal amplitude required to address gait deficits), and how real-time DBS adaptations based on this threshold influence band power.

(c) Representative example of changes in STN dynamics during movement (participant P3), shown under both standard-of-care therapy and activity-dependent DBS therapy. Activity-dependent adaptations in DBS amplitude are triggered using broadband gamma power as control signal, centered at 40Hz. PSD is computed using Welch's method (mean ± 95% confidence interval of the estimate).

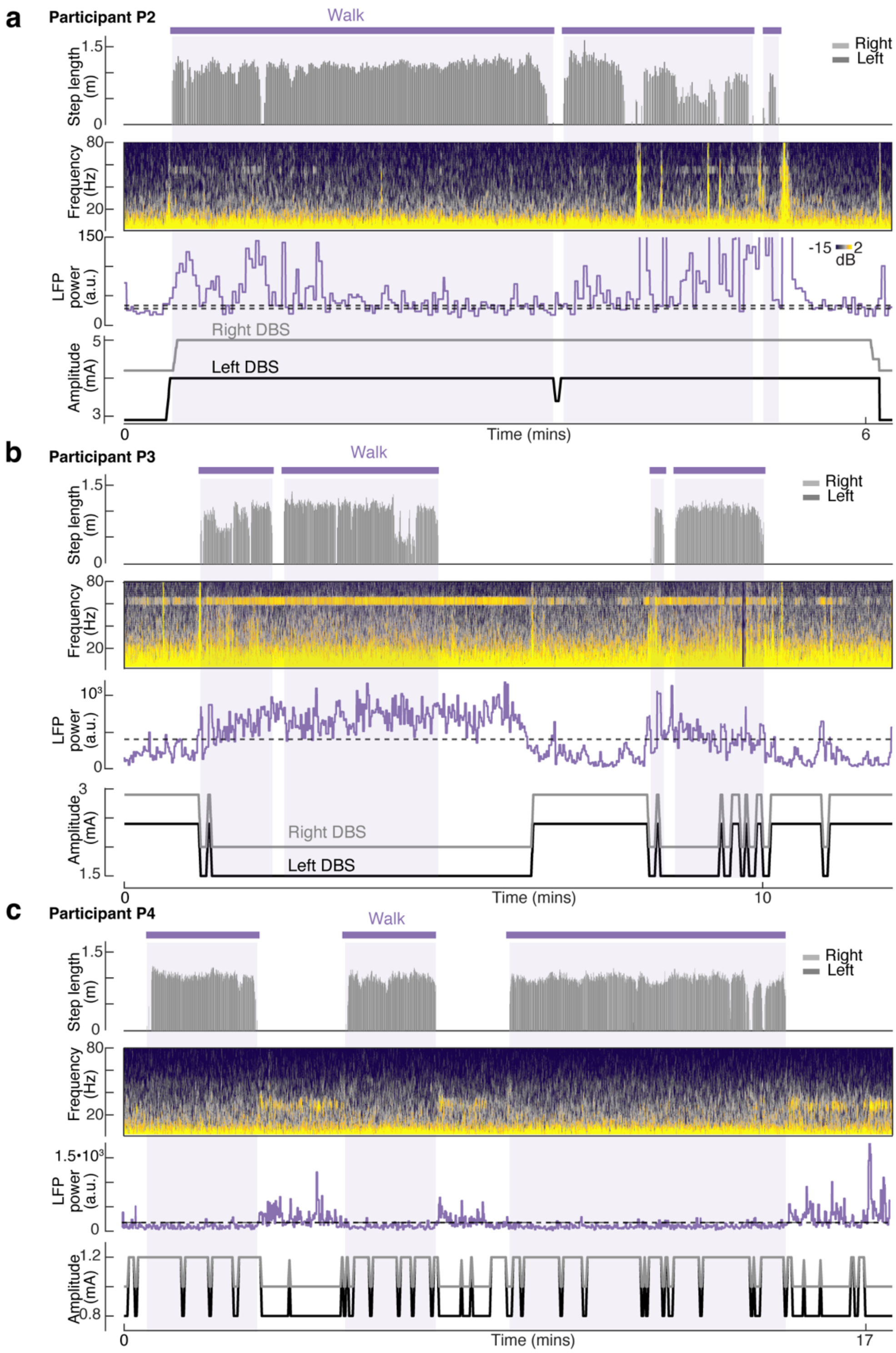

Supplementary Figure 5 | Sustained activity-dependent DBS during out-of-laboratory assessments across implemented control policies

(a) Illustrative example of aligned step-length trajectories (*top*), raw STN LFP spectrograms (5–80 Hz, *middle*), LFP band-power used to trigger DBS amplitude adaptations, and corresponding stimulation adjustments (*bottom*) in Participant P2. DBS amplitude was increased to mitigate locomotor deficits. P2 exhibited gait-related LFP power-synchronization in the narrow-gamma band (55.6 Hz). Accordingly, the dual-threshold mode was used to control DBS adaptations. Temporal parameters (average duration, blanking period, and onset duration) governing activity-dependent DBS responsiveness were tuned to compensate for fluctuations in the control-band stability, which varied with task complexity and physical effort (e.g., outdoor walking, stairs). The dual-threshold configuration further enhanced stability under variable conditions.

(b) Same representation for Participant P3. DBS amplitude was decreased to mitigate locomotor deficits. P3 exhibited gait-related LFP-power synchronization in the narrow-band gamma (62.5 Hz). Accordingly, the single threshold inverse mode was used to control DBS adaptations. Temporal parameters (average duration, blanking

period, and onset duration) governing activity-dependent DBS responsiveness were tuned to compensate for fluctuations in the control-band stability, which varied with task complexity and physical effort (e.g., outdoor walking, stairs).

(c) Same representation for Participant P4. DBS amplitude was increased to mitigate locomotor deficits. P4 exhibited gait-related LFP power-desynchronization in high-beta (28.32 Hz). Accordingly, the single threshold inverse mode was used to control DBS adaptations. Temporal parameters (average duration, blanking period, and onset duration) governing activity-dependent DBS responsiveness were tuned to compensate for fluctuations in the control-band stability, which varied with task complexity and physical effort (e.g., outdoor walking, stairs).

Supplementary Table 1 | Demographic and clinical characteristics of all 35 participants included in the characterization of physiological principles underlying therapy-specific encoding of locomotor activities in STN dynamics, and the development of neural decoders. (AR: Akinetic-rigid, T: Tremor, ND: No Dopamine). Sex was recorded based on sex assigned at birth and self-reported by participants. Sex- and gender-based analyses were not performed, as the study was not designed to assess sex- or gender-related differences.

Demographic information					Clinical profile				
**ID**	**Sex**	**Onset of disease**	**Age**	**Disease duration**	**Dominant symptoms (AR or T)**	**LEDD pre-op (mg eq.)**	**Total UPDRSIII OFF/ON pre-surgery**	**Most affected hemibody**	**Medication status at the start of the experimental session**
**PD1**	M	2015	73	6	AR	723	31/25	LEFT	OFF > 12h
**PD2**	F	2016	69	5	T	175	99/89	RIGHT	ND
**PD3**	M	2001	72	20	AR		40/18	LEFT	OFF > 12h
**PD4**	M	2008	64	13	AR+T	1490	32/8	RIGHT	OFF > 12h
**PD5**	M	2009	56	12	AR	1450	38/11	LEFT	Residual
**PD6**	F	2014	56	8	AR+T	700	35/11	RIGHT	OFF > 12h
**PD7**	F	2005	74	17	AR	491	47/22	RIGHT	Residual
**PD8**	M	2012	67	10	AR	1600	22/7	RIGHT	Residual
**PD9**	M	2018	64	4	AR+T	774	39/9	RIGHT	Residual
**PD10**	M	2013	59	9	AR	1400	17/4	RIGHT	OFF > 12h
**PD11**	M	2013	71	9	AR+T	1208	36/6	LEFT	OFF > 12h
**PD12**	M	2015	58	7	AR+T	0	20/ND	LEFT	ND
**PD13**	F	2010	45	12	AR	1698	40/17	RIGHT	OFF > 12h
**PD14**	M	2015	70	8	AR+T		30/14	RIGHT	OFF > 12h
**PD15**	M	2018	67	5	AR	625	34/20	LEFT	OFF > 12h
**PD16**	M	2013	66	10	AR	250	49/8	BOTH	OFF > 12h
**PD17**	M	2006	57	17	AR+T	2160	40/10	BOTH	OFF > 12h

**PD18**	M	2013	63	10	AR	1650	53/32	LEFT	OFF > 12h
**PD19**	F	2010	67	13	AR+T	1075	68/32	LEFT	Residual
**PD20**	M	2015	60	9	AR+T	1293	47/2	LEFT	OFF > 12h
**PD21**	M	2015	54	8	AR+T	1862	54/18	LEFT	OFF > 12h
**PD22**	F	2011	71	12	AR+T	1500	48/28	LEFT (AR)<br>RIGHT (T)	OFF > 12h
**PD23**	F	2018	61	5	AR+T	750	30/6	RIGHT	ND
**PD24**	M	2016	72	7	AR+T	875	34/10	RIGHT	OFF > 12h
**PD25**	M	2014	59	9	AR	1875	31/4	LEFT	Residual
**PD26**	F	2013	52	11	AR+T	1422	33/18	LEFT	Residual
**PD27**	F	2015	68	9	AR	1615	67/22	RIGHT	OFF > 12h
**PD28**	F	2015	70	9	AR+T	1313	48/16	BOTH	OFF > 12h
**PD29**	M	2016	68	8	AR+T	819	32/10	LEFT	OFF > 12h
**PD30**	M	2007	0	17	AR	1049	49/16	LEFT	OFF > 12h
**PD31**	M	2007	62	17	AR	1667	23.5/8	RIGHT	OFF > 12h
**PD32**	M	2013	61	11	AR	1300	10/9	RIGHT	OFF > 12h
**PD33**	F	2010	73	11	T	800	25/3	LEFT(sup)<br>RIGHT (inf)	OFF > 12h
**PD34**	F	2014	59	10	AR+T	941	47/11	LEFT	OFF > 12h
**PD35**	M	2016	73	8	AR	1737.5	37/19	LEFT	Residual

Supplementary Table 2 | Locomotor tasks and therapeutic conditions recorded in all 35 participants included in the characterization of the physiological principles underlying therapy-specific encoding of locomotor activities in STN dynamics, and leveraged for the development of neural decoders.

Cohort	ID	Hems recorded	Locomotor tasks	Conditions recorded	Additional conditions recorded	Comments	Group A	B	C	D	E	F
	**PD1**	L+R					x	x	x			
	**PD2**	L+R				Does not take L-DOPA			x			
	**PD3**	L+R					x	x	x			
	**PD4**	L+R					x	x	x			
	**PD5**	L+R	Sit<br>Stand<br>Walk	**OFF-therapy**<br>STN DBS alone<br>STN DBS + low / mid L-DOPA (15 mins, 30 mins)<br>**Standard-of-care STN DBS + L-DOPA**	Low Amplitude STN DBS		x	x	x		x	
**1a**	**PD6**	L+R					x	x	x			
	**PD7**	L+R					x	x	x			
	**PD8**	L+R					x	x	x			
	**PD9**	L+R					x	x	x			
	**PD10**	L+R					x	x	x			
	**PD11**	R					x					
	**PD12**	R			STN DBS alone	Does not take L-DOPA			x			
	**PD13**	L+R					x					
	**PD14**	R	Sit<br>Stand<br>walk<br>Obstacle	**OFF-therapy**<br>**Standard-of-care STN DBS + L-DOPA**			x					
**1b**	**PD15**	L+R					x					
	**PD16**	L+R					x					
	**PD17**	L+R					x					
	**PD18**	L+R					x					

	**PD19**	L+R					x					
	**PD20**	L+R					x					
	**PD21**	L+R			STN DBS alone<br>Alternating DBS		x		x			x
	**PD22**	L			STN DBS alone<br>Alternating DBS				x	x		x
	**PD23**	L+R			Alternating DBS	Does not take L-DOPA						x
	**PD24**	L+R			STN DBS alone<br>Alternating DBS<br>Low Amplitude STN DBS				x	x	x	x
	**PD25**	L								x	x	
	**PD26**	L+R								x		
**2a**	**PD27**	L+R	Sit<br>Stand<br>Walk	**OFF-therapy<br>L-DOPA alone**						x		
	**PD28**	L+R								x		
	**PD29**	L+R								x		
	**PD30**	L+R			Low Amplitude STN DBS<br>STN DBS alone				x	x	x	
	**PD31**	L+R								x		
	**PD32**	R								x	x	
	**PD33**				Low Amplitude STN DBS						x	
**2b**	**PD34**		Sit<br>Stand<br>Walk	**OFF-therapy<br>Standard-of-care STN DBS + L-DOPA**	Low Amplitude STN DBS						x	
	**PD35**				Low Amplitude STN DBS						x	

Supplementary Table 3 | Demographic and clinical characteristics of the 8 participants recruited for the proof-of-concept feasibility study assessing the safety and preliminary efficacy of activity-dependent adaptive DBS. This includes MDS-UPDRS Part III scores (Movement Disorder Society Unified Parkinson's Disease Rating Scale) assessed in the low dopaminergic state. Participants P1-P4 met the eligibility criteria after Session 1 and were enrolled in the interventional phase of the study. For these participants, the most bothersome gait deficits targeted with activity-dependent adaptive DBS are reported, along with the affected body side and the corresponding Freezing of Gait Questionnaire (FOG-Q) score (range: 0–24), when FOG was among the presenting symptoms. (cDBS: continuous DBS therapy).

Demographic and Clinical information										Gait deficit			
**Participant**	**Age**	**Sex**	**Disease duration (yrs)**	**Months from DBS implant**	**Type**	**MDS-UPDRS III Low MED**	**Standard DBS therapy**	**Eligibility matched**	**Reason for exclusion**	**First**	**Second**	**Body side**	**FOGQ**
**P1**	61	F	13	8	Akinetic-rigid and tremor	41	cDBS	YES	—	Blocked leg	—	Right	—
**P2**	61	M	11	19	Akinetic-rigid	29	cDBS	YES	—	FoG	—	Right (sup) Left (inf)	12
**P3**	63	M	12	11	Akinetic-rigid	14	cDBS	YES	—	FoG	Lower limb dystonia	Right	12
**P4**	66	M	17	4	Akinetic-rigid and tremor	15	adaptive DBS	YES	—	Leg weakness	—	Right	—
**P5**	51	F	13	4	Akinetic-rigid and tremor	25	cDBS	NO	Gait deficit not severe enough	—	—	—	—
**P6**	69	M	18	3	Akinetic-rigid and tremor	40	cDBS	NO	Gait deficit not severe enough	—	—	—	—
**P7**	72	M	12	30	Akinetic-rigid and tremor	37	cDBS	NO	Cognitive impairments	—	—	—	—
**P8**	60	M	16	41	Akinetic-rigid	26	cDBS	NO	Gait deficit not severe enough	—	—	—	—

Supplementary Table 4 | Two-way repeated-measures ANOVA results for therapeutic condition (OFF-therapy versus standard-of-care L-DOPA + STN DBS) and locomotor activity (sit, stand, walk) - Figure 2a

Frequency band	Effect	F (df_1, df_2)	p-value	η^2	Significant post-hoc results
**Gamma**	Therapeutic condition	F(1,27) = 13.56	0.001	0.33	Lower power under L-DOPA + STN-DBS for all activities (sit p = 0.002; stand p = 0.007; walk p = 0.0003)
	Locomotor activity	F(2,54) = 44.18	1.9×10^{-10}	0.62	OFF-therapy: sit < stand < walk (all p < 0.05)
	Interaction	F(2,54) = 5.00	0.010	0.16	Under L-DOPA + STN-DBS: no stand–walk difference (p = 1.0); overall attenuation of modulation
**High-beta**	Therapeutic condition	F(1,26) = 21.36	9.1×10^{-5}	0.45	Reduced power under L-DOPA + STN-DBS across all activities (sit p = 1.4×10^{-4}; stand p = 3.9×10^{-4}; walk p = 0.0016)
	Locomotor activity	F(2,52) = 25.14	9.3×10^{-7}	0.49	OFF-therapy: sit > stand > walk (all p < 0.005)
	Interaction	F(2,52) = 2.60	0.084	0.09	Under L-DOPA + STN-DBS: sit–stand difference abolished (p = 0.18); walk remained lower (p < 0.01)
**Low-beta**	Therapeutic condition	F(1,29) = 4.43	0.044	0.13	Only sitting decreased under therapy (p = 0.007)
	Locomotor activity	F(2,58) = 0.39	0.68	—	No significant effects (all p > 0.9)
	Interaction	F(2,58) = 0.10	0.91	—	No interaction observed

Supplementary Table 5 | Two-way repeated-measures ANOVA results for therapeutic condition (OFF therapy vs STN DBS alone, and OFF-therapy vs L-DOPA alone) and locomotor activity (sit, stand, walk) - Figure 2b

Frequency band	Therapy	Effect	F (df_1, df_2)	p-value	Significant post-hoc results
**Gamma**	**STN-DBS**	Condition	F(1,22) = 26.36	3.8×10^{-5}	Global reduction of gamma power across all activities (sit p = 1.1×10^{-5}; stand p = 3.4×10^{-4}; walk p = 4.9×10^{-5})
		Activity	F(2,44) = 78.83	2.2×10^{-11}	Progressive increase OFF-therapy (sit < stand < walk; all p < 0.02)
		Interaction	F(2,44) = 7.46	0.002	Flattened modulation under DBS (no stand–walk difference)
	**L-DOPA**	Condition	F(1,13) = 7.04	0.020	Global increase across activities (sit < stand < walk; all p < 0.02)
		Activity	F(2,26) = 51.08	9.1×10^{-7}	Progressive increase across locomotor states
		Interaction	F(2,26) = 7.91	0.002	Enhanced modulation with movement under L-DOPA
**High-beta**	**STN-DBS**	Condition	F(1,21) = 20.79	1.7×10^{-4}	Reduced power across all activities (sit p = 0.0003; stand p = 0.0008; walk p = 0.0001)
		Activity	F(2,42) = 13.64	6.9×10^{-5}	Sit > walk (p = 0.0049); stand > walk (p = 0.021)
		Interaction	—	—	No interaction observed
	**L-DOPA**	Condition	F(1,13) = 11.57	0.0047	Lower high-beta power across activities (sit p = 0.042; stand p = 0.004; walk p = 0.007)
		Activity	F(2,26) = 6.63	0.009	Sit > walk (p = 0.0027); stand > walk (p = 0.0097)
		Interaction	—	—	No interaction observed
**Low-beta**	**STN-DBS**	Condition	—	p > 0.7	No significant effects under DBS
		Activity	—	p > 0.7	Unmodulated across activities
		Interaction	—	—	—
	**L-DOPA**	Condition	F(1,10) = 20.94	0.001	Consistent suppression across activities (sit p = 0.0049; stand p = 0.0007; walk p = 0.0011)
		Activity	—	—	No main effect of activity
		Interaction	—	—	—

Supplementary Table 6 | Two-way repeated-measures ANOVA results across DBS amplitudes and locomotor activities - Figure 2f

Frequency band	Effect	F (df_1, df_2)	p-value	Significant post-hoc results
**Gamma**	Locomotor activity	$F(1,10) = 317.3$	6.6×10^{-9}	Band-power higher during movement than rest at amplitudes 1 ($p = 0.0013$) and 3 ($p = 0.039$)
	DBS amplitude	$F(1,10) = 6.09$	0.033	Gamma power decreased with increasing amplitude; significant between amplitudes 1 vs 3 at rest ($p = 0.012$)
	Activity × amplitude interaction	$F(1,10) = 4.97$	0.050	Decrease with amplitude stronger at rest; not significant during movement ($p > 0.2$)

Supplementary Table 7 | Bonferroni-corrected p-values from two-sided unpaired Welch's t-tests comparing gait parameters between activity-dependent DBS and standard-of-care DBS. Values >1 may occur after correction and are reported without upper bound. Significant p-values are highlighted in bold – Figure 6e

GAIT PARAMETER	P1	P2	P3	P4
**Velocity**	**8.08×10^{-5}**	3.23	**1.40×10^{-23}**	**2.92×10^{-20}**
**Stride length**	**3.01×10^{-5}**	2.47	**2.25×10^{-21}**	**2.43×10^{-9}**
**Swing-stance ratio**	1.42	**3.60×10^{-3}**	**4.17×10^{-3}**	**5.89×10^{-6}**
**Supination angle**	**8.48×10^{-4}**	1.49	8.84	0.41
**Strike angle**	**1.32×10^{-7}**	2.05	0.55	0.054
**Stride duration**	**0.0425**	6.42	**8.85×10^{-5}**	**1.42×10^{-22}**
**Lateral swing**	6.74	6.81	0.454	6.78
**Heel clearance**	**2.68×10^{-15}**	0.440	0.112	0.568
**Double support time**	**6.26×10^{-4}**	0.203	**0.0204**	**1.86×10^{-5}**
