# Supplementary material for: Activity-dependent adaptive deep brain stimulation improves gait in Parkinson’s disease
Source: Nat Med. 2026 Jun 15;32(8):2815–30. doi: 10.1038/s41591-026-04432-4 (PMC13472876; doi:10.1038/s41591-026-04432-4)
Supplement: Supplementary file 2 [file 41591_2026_4432_MOESM2_ESM.pdf]

nature portfolio

Corresponding author(s): Prof. Eduardo Martin Moraud

Last updated by author(s): Apr 12, 2026

Reporting Summary

Nature Portfolio wishes to improve the reproducibility of the work that we publish. This form provides structure for consistency and transparency in reporting. For further information on Nature Portfolio policies, see our Editorial Policies and the Editorial Policy Checklist.

Statistics

For all statistical analyses, confirm that the following items are present in the figure legend, table legend, main text, or Methods section.

n/a	Confirmed	
☐	☒	The exact sample size (*n*) for each experimental group/condition, given as a discrete number and unit of measurement
☐	☒	A statement on whether measurements were taken from distinct samples or whether the same sample was measured repeatedly
☐	☒	The statistical test(s) used AND whether they are one- or two-sided<br>*Only common tests should be described solely by name; describe more complex techniques in the Methods section.*
☐	☒	A description of all covariates tested
☐	☒	A description of any assumptions or corrections, such as tests of normality and adjustment for multiple comparisons
☐	☒	A full description of the statistical parameters including central tendency (e.g. means) or other basic estimates (e.g. regression coefficient) AND variation (e.g. standard deviation) or associated estimates of uncertainty (e.g. confidence intervals)
☐	☒	For null hypothesis testing, the test statistic (e.g. *F*, *t*, *r*) with confidence intervals, effect sizes, degrees of freedom and *P* value noted<br>*Give P values as exact values whenever suitable.*
☒	☐	For Bayesian analysis, information on the choice of priors and Markov chain Monte Carlo settings
☒	☐	For hierarchical and complex designs, identification of the appropriate level for tests and full reporting of outcomes
☐	☒	Estimates of effect sizes (e.g. Cohen's *d*, Pearson's *r*), indicating how they were calculated

Our web collection on statistics for biologists contains articles on many of the points above.

Software and code

Policy information about availability of computer code

Data collection

- LFP recordings: Medtronic DBS Clinician Programmer Application A610 v3.0 and v5.0
- EMG activity: Delsys USA EMG sensors
- kinematics: Delsys USA EMG sensors, Vicon optoeletronic motion capture system, sensorized shoes with embedded IMU (Nushu, Magnes, Switzerland), video-based motion capture system (OpenCV and RTMPose)

Data analysis

All software and corresponding versions used to analyze data:
- Matlab v2022b and later by Mathworks
- Lead-DBS Matlab toolbox
- http://github.com/matteonocilli/GAIT_tool
- https://spm1d.org
- Python version 3.8 and later
- https://github.com/dbdq/neurodecode

For manuscripts utilizing custom algorithms or software that are central to the research but not yet described in published literature, software must be made available to editors and reviewers. We strongly encourage code deposition in a community repository (e.g. GitHub). See the Nature Portfolio guidelines for submitting code & software for further information.

Data

Policy information about availability of data

All manuscripts must include a data availability statement. This statement should provide the following information, where applicable:

- Accession codes, unique identifiers, or web links for publicly available datasets
- A description of any restrictions on data availability
- For clinical datasets or third party data, please ensure that the statement adheres to our policy

Anonymised data supporting the findings of this study are available in Zenodo DOI: 10.5281/zenodo.19371520. Codes developed for data analysis of the shown results are as well available in Zenodo DOI: 10.5281/zenodo.19371520. Other clinical data are subject to ethical restrictions and are available from the corresponding author upon reasonable request to the corresponding author at Eduardo.Moraud@epfl.ch

Research involving human participants, their data, or biological material

Policy information about studies with human participants or human data. See also policy information about sex, gender (identity/presentation), and sexual orientation and race, ethnicity and racism.

Reporting on sex and gender	Sex (male/female) was recorded based on sex assigned at birth and self-reported by participants. Supplementary Table 1 (35 participants involved in Part 1 study) and Supplementary Table 3 (8 participants involved in Part 2 study). Gender information was not explicitly collected during the study. Sex- and gender-based analyses were not performed, as the study was not designed to assess sex- or gender-related differences.
Reporting on race, ethnicity, or other socially relevant groupings	Race, ethnicity, or other socially relevant grouping information was not explicitly collected or analyzed during this study.
Population characteristics	The study population consisted of individuals with Parkinson's disease who provided written informed consent prior to participation. Demographic information, including age, sex, and disease characteristics, was collected and is summarized in Supplementary Tables 1 and 3. All participants received bilateral STN DBS leads (SenSight™, Medtronic, USA) and a Percept™ neurostimulator (Medtronic, USA), enabling simultaneous neural recording and stimulation.<br>In addition, six healthy controls (3 male, 3 female) were recruited to serve as a reference group for physiological gait, enabling comparisons with gait quality in Parkinson's disease participants under both conventional and activity-dependent DBS.<br>Further information on the ADAPGAIT clinical trial (Part 2) is available at the following link: https://clinicaltrials.gov/study/NCT06791902.
Recruitment	Participants for Part 1 study were recruited within the cohort of patients hospitalized at CHUV for DBS implantation surgery. Recordings were performed within seven days following DBS implantation.<br>Participants for the ADAPGAIT clinical trial (Part 2 study) were recruited from the patient population routinely followed by the Neurology Department at CHUV, as well as through referrals from expert neurologists affiliated with regional primary care facilities and rehabilitation centers.<br>All recruited individuals were screened by the study investigators to assess eligibility for enrollment. Initial contact with eligible participants was established by the investigators, either via email or phone, to arrange an in-person or video consultation.<br>Expenses for participants recruited in the ADAPGAIT clinical trial were covered by the sponsor of the study (Centre Hospitalier Universitaire Vaudois - CHUV).<br>The sponsor covered (i) participant's transportation fees from participant's home to CHUV, for the participant and one caretaker traveling together. (ii) Meals for the participant and one caretaker during sessions that take place at CHUV (up to 20 CHF/ day/person). (iii) If needed, the sponsor also provided an accommodation hosting two persons in Lausanne, including the cost of dinner (up to 27CHF/person). Participants received a compensation of 120 CHF per day for their participation.
Ethics oversight	All experiments of the Part 1 study were approved by the Ethical Committee of the Canton de Vaud, Switzerland (ref: PB_2017-00064). Written informed consent was obtained from all participants prior to each recording, after a comprehensive explanation of the study's purpose and potential consequences.<br>The feasibility clinical trial evaluating activity-dependent adaptive DBS (Part 2 study) was approved by the Swiss Ethics Committee (Swissethics BASEC 2024-D0066; protocol no. 10001378, approved on 21 November 2024) and the Swiss regulatory authority (Swissmedic SNCTP 000006185; protocol no. 102731544, approved on 23 December 2024). An amendment , which allowed to expand the target population to participants with Medtronic Percept RC (Model 35300) neurostimulator, was approved by Swiss Ethics Committee on 22.08.2025 and by Swiss regulatory authority on 29.08.2025.<br>The clinical trial was conducted in accordance with the Declaration of Helsinki and is registered at ClinicalTrials.gov (NCT06791902).

Note that full information on the approval of the study protocol must also be provided in the manuscript.

Field-specific reporting

Please select the one below that is the best fit for your research. If you are not sure, read the appropriate sections before making your selection.

☒ Life sciences ☐ Behavioural & social sciences ☐ Ecological, evolutionary & environmental sciences

For a reference copy of the document with all sections, see nature.com/documents/nr-reporting-summary-flat.pdf

Life sciences study design

All studies must disclose on these points even when the disclosure is negative.

Sample size	- A total of 41 participants were enrolled in Part 1 to investigate STN encoding of locomotor activities.<br>- A total of 8 participants were enrolled in Part 2 to assess the safety and preliminary efficacy of activity-dependent adaptive DBS
Data exclusions	Of the 41 participants initially enrolled in Part 1, two were unable to complete the required set of motor tasks due to poor physical condition and were not recorded. Among the remaining 39 participants who underwent recordings, three were excluded due to severe ECG-related artifacts affecting all LFP channels, rendering the signals unusable. One additional participant was excluded because the neurostimulator was operating in “cycling” mode, which prevented consistent comparisons across therapeutic conditions.<br>Thus, 35 participants (70 hemispheres) were retained for the analyses. Of these, 7 hemispheres were further excluded based on post-operative anatomical reconstructions, which indicated that the selected contacts were likely located outside the motor subregion of the STN. This anatomical uncertainty was supported by the absence of detectable LFP modulations across the frequency spectrum and across therapeutic conditions.<br><br>For the feasibility clinical trial on activity-dependent DBS (Part 2), four participants were excluded during the eligibility assessment as they did not meet inclusion criteria: three due to insufficient locomotor deficits and one due to cognitive impairment precluding compliance with task instructions.
Replication	Part 1 study (Electrophysiology): Participants performed up to 20 consecutive repetitions of each motor task (for example, straight walking bouts and obstacle avoidance) to ensure repeatability of the observed neural modulations. Under some conditions (for example, OFF therapy), some participants were unable to complete all repetitions due to fatigue or symptom severity. A minimum of 12 repetitions was required for inclusion in the analyses.<br>Part 2 study (activity-depdent therapy): To evaluate the acute effects of activity-dependent DBS, we selected clinical tests and motor tasks tailored to each participant’s most disabling gait impairment. Tasks were performed under both activity-dependent DBS and standard-of-care DBS, with conditions randomized across repetitions.<br>Long-term reproducibility falls outside the scope of this feasibility study. All analyses were independently performed and routinely cross-checked by two or three investigators (S.S., V.d.S. and E.M.M., depending on the task and condition) to ensure accuracy and consistency.
Randomization	Investigators delivering the intervention were not blinded, as their expertise was required to optimize stimulation during evaluations. Neurology experts and clinical staff performing standardized clinical assessments (for example, MDS-UPDRS) were blinded to stimulation conditions. Similarly, participants were blinded to stimulation conditions throughout the assessments, although they may have perceived differences in stimulation effects. Stimulation conditions were randomized. Blinding to L-DOPA effects was not feasible.
Blinding	Investigators were not blinded, as their expertise was required to optimize and deliver the intervention during evaluations. Stimulation conditions were randomized and blinded to participants until session completion. However, we cannot exclude the possibility that participants perceived differences in stimulation effects. Blinding to the effects of L-DOPA was not feasible.

Reporting for specific materials, systems and methods

We require information from authors about some types of materials, experimental systems and methods used in many studies. Here, indicate whether each material, system or method listed is relevant to your study. If you are not sure if a list item applies to your research, read the appropriate section before selecting a response.

Materials & experimental systems

n/a	Involved in the study	
☒	☐	Antibodies
☒	☐	Eukaryotic cell lines
☒	☐	Palaeontology and archaeology
☒	☐	Animals and other organisms
☐	☒	Clinical data
☒	☐	Dual use research of concern
☒	☐	Plants

Methods

n/a	Involved in the study	
☒	☐	ChIP-seq
☒	☐	Flow cytometry
☐	☒	MRI-based neuroimaging

Clinical data

Policy information about clinical studies

All manuscripts should comply with the ICMJE guidelines for publication of clinical research and a completed CONSORT checklist must be included with all submissions.

Clinical trial registration	AdapGAIT Clinicaltrials.gov identifier: NCT06791902
Study protocol	https://clinicaltrials.gov/study/NCT06791902
Data collection	Data were collected in dedicated gait laboratories at the Centre Hospitalier Universitaire Vaudois (CHUV), equipped with specialized systems for monitoring whole-body kinematics, bilateral electromyographic activity, and synchronized brain signals. Participants were also evaluated in out-of-laboratory settings using sensorized shoes capable of computing objective gait quality metrics in real-world environments. Recruitment for the clinical trial began in February 2025. The eight participants included in this study were enrolled between February 2025 and Jan 2026.

Outcomes

ADPAGAIT is a clinical trial with Investigational Medical Device (an investigational version of the DBS Clinician Programmer enabled full access of the aDBS functionalities on the INS).

Primary outcome:
- Occurrence of adverse events (AEs) and serious adverse events (SAEs) that are deemed related or possibly related to the study procedures, or the use of the investigational system during the duration of the study.

Secondary outcomes:
- Comparison of gait performance under (i) standard (continuous) DBS and (ii) DBS protocols that automatically adapt stimulation based on gait biomarkers. Tasks: Timed up-and-go task (TUG), 10m walk test (10MWT). Measured variables: Time to perform tasks, kinematic and electromyographic analyses.
- Comparison of freezing of gait occurrences under (i) standard (continuous) DBS and (ii) DBS protocols that automatically adapt stimulation based on Freezing of Gait (FOG) biomarkers. Tasks: FOG circuit, 360° degree turn test. Measured variables: Time to perform tasks, kinematic and electromyographic analyses.
- Evaluation of changes in neural patterns in response to adaptive protocols that are aligned in time to biomarkers of locomotor states. Measured variables: Changes in neural signals recorded with the implantable system during the aforementioned locomotor tasks.
- Evaluation of the sustained efficacy of activity-dependent DBS in the context of everyday life activities: Maintain activity-dependent DBS for a half day in the hospital, as the participant performs activities of daily living. Monitor gait quality, freezing of gait and subjective feeling of the participant by recording with sensorized shoes that track stepping patterns, EMG sensors to record muscle activity, and doing a questionnaire.

Assessment of outcomes:

Clinical Assessments
- 10MWT: Gait speed assessed over a 10 m walkway. Performed three times at self?selected and fast speeds. Kinematic, EMG, and LFP recordings were collected.
- TUG: Time to stand, walk 5 m, turn, return, and sit. Three repetitions per condition. Kinematic, EMG, and LFP recordings were collected.
- 360° turn test: Time and number of steps to complete a full turn (four repetitions per direction). Kinematic, EMG, and LFP recordings were collected.
- FoG circuit: Custom circuit designed to assess the occurrence of freezing of gait. Participants were asked to perform tasks known to favour FOG occurrence. A cognitive or supplementary motor task may be added to trigger FOG. Up to 10 repetitions; stopped if >150 s. Kinematic, EMG, and LFP recordings were collected.
- MDS?UPDRS Part III: Standardized motor examination performed by a trained clinician.

Movement analysis
- Assessment of gait or lower-limb motor tasks and LFP activity during predefined movement tasks, with concurrent kinematic and EMG recordings.

Daily life performance Assessments
- FOG?Q: Six?item self?administered questionnaire assessing FOG severity.
- Evaluation of aDBS in activities of daily living questionnaire: self-administered questionnaire designed within the scope of this study. It evaluated the perceived gait quality, and overall motor performance under activity-dependent DBS with respect to standard-of-care DBS.
- Out?of?lab monitoring: To evaluate the evolution of the gait pattern in an ecological environment, participants wore CE-marked sensorized shoes. To monitor the muscular activity of the key muscles during walking, EMG sensors (Delsys, USA) could be used.

Adverse events

AEs were defined as any untoward medical occurrence, unintended disease or injury, or untoward clinical signs (including abnormal laboratory findings) in subjects, users or other persons, whether or not related to the investigational device or procedures. At each visit, participants were asked whether they encountered any difficulties or if anything happened since their last visit. If the answer was positive and the AE is identified, the following were documented: nature, onset and end dates, intensity (mild/moderate/severe), seriousness, causality (device/procedure: not related, possible, probable, causal), expectedness, response measures, resolution status, and investigator initials.

Plants

Seed stocks	n/a
Novel plant genotypes	n/a
Authentication	n/a

Magnetic resonance imaging

Experimental design

Design type	Structural Imaging only
Design specifications	Structural Imaging only
Behavioral performance measures	Structural Imaging only

Acquisition

Imaging type(s)	Structural MRI
Field strength	3 Tesla
Sequence & imaging parameters	MRI was performed on a 3T MAGNETOM MR scanner equipped with a 64-channel head-coil (Siemens Healthineers, VA60A-OCT2 Software version,Erlangen,Germany).<br>For each participant, anatomical data were acquired using injection 0.2ml/kg of Gadolinium (Dotarem® 0.5 mmol Gd/ml, Guerbet, Roissy, France).<br>The standard MRI protocol comprised the following 5 pulse sequences:<br>a) 3D sagittal SPACE (Sampling Perfection with Application optimized Contrast using different flip angle Evolution) T2-weighted FLAIR (Fluid-attenuated inversion recovery) (repetition time (TR), 4900 ms; echo time (TE), 386 ms; inversion time (TI), 1580ms ; voxel size, 0.5×0.5×9 mm3);<br>b) 3D sagittal SPACE T2-weighted (repetition time (TR), 2400 ms; echo time (TE), 221 ms; voxel size, 1.0×1.0×1.0 mm3);<br>c) Axial DTI (repetition time (TR), 10300 ms; echo time (TE), 84 ms; voxel size, 2.2×2.2×2.2 mm3); 64directions ; b0/b1000s/mm2<br>d) 3D sagittal T1-weighted MPRAGE(Magnetization Prepared Ramp Acquired Gradient Echo) (repetition time (TR), 1900 ms; echo time (TE), 2.43 ms; inversion time (TI), 900ms ; voxel size, 1.0×1.0×1.0 mm3);<br>e) 3D sagittal T1-weighted MP2RAGE (Magnetization Prepared 2 Rapidly Acquired Gradient Echo) with FLAWS (Fluid and white matter suppression) Reconstruction (repetition time (TR), 5000 ms; echo time (TE), 2.98 ms; inversion time (TI), 700 / 2500ms ; voxel size, 1.0×1.0×1.0 mm3);
Area of acquisition	whole brain

Diffusion MRI ☒ Used ☐ Not used

Parameters: *Specify # of directions, b-values, whether single shell or multi-shell, and if cardiac gating was used.*

Preprocessing

Preprocessing software	MRI image acquisition did not require a preprocessing software. No preprocessing software was used.
Normalization	MRI image acquisition did not require normalization. Normalization was not used.
Normalization template	MRI image acquisition did not require a normalization template. Normalization template was not used.
Noise and artifact removal	Shim boxes were applied to correct for magnetic field inhomogeneities
Volume censoring	MRI image acquisition did not require volume sensoring. Volume censoring was not used.

Statistical modeling & inference

Model type and settings	n/a
Effect(s) tested	n/a

Specify type of analysis: ☐ Whole brain ☒ ROI-based ☐ Both

Anatomical location(s)	*Describe how anatomical locations were determined (e.g. specify whether automated labeling algorithms or probabilistic atlases were used).*
Statistic type for inference (See Eklund et al. 2016)	Imaging was only used to document lead locations without any statistical hypothesis testing.
Correction	n/a

Models & analysis

n/a	Involved in the study
☒	☐ Functional and/or effective connectivity
☒	☐ Graph analysis
☒	☐ Multivariate modeling or predictive analysis
